# Type 2 diabetes burden, inequalities, and epidemiological patterns among postmenopausal women, 1990–2021: a global ecological modelling study

**DOI:** 10.1186/s12916-026-05107-0

**Published:** 2026-08-11

**Authors:** Laiang Yao, Yuelong Ji, Wenjun Fan, Andrew Odegaard

**Affiliations:** 1 Department of Epidemiology and Biostatistics, Joe C Wen School of Population & Public Health, University of California, Irvine, USA; 2 Center of Global Cardiometabolic Health and Nutrition, University of California, Irvine, USA; 3 Department of Maternal and Child Health, School of Public Health, Peking University, Beijing, China

**Keywords:** Type 2 diabetes mellitus, Postmenopausal women, Global health, Global burden of disease, Health equity and equality

## Abstract

**Background:**

Type 2 diabetes mellitus (T2DM) is a major global cause of mortality and morbidity globally. Postmenopausal women bear a disproportionately high burden due to the intertwined effects of aging, biological changes, and structural disadvantages. This study aims to assess the global burden, temporal trends, health inequalities, associated risks and divergent epidemiological patterns of T2DM among this population.

**Methods:**

Data regarding the incidence and mortality rate of T2DM in postmenopausal women were obtained from the Global Burden of Disease Study 2021. Women aged ≥ 55 years were used as an age-based operational proxy for postmenopausal status. Temporal trends were assessed using estimated annual percent changes (EAPC). We applied several machine learning methods, including K-means and hierarchical clustering, ensemble tree-based models (Random Forest and XG Boost) with SHAP analysis to assess the divergent burden and risk profiles and among postmenopausal women globally.

**Results:**

Globally, the incidence rate of T2DM among postmenopausal women increased by 1.40% per year (95% CI 1.35–1.44) since 1990, reaching nearly 600 cases per 100,000 population in 2021. Marked health inequalities persisted, with substantially higher mortality burdens observed in lower-SDI (Sociodemographic index) countries. Three distinct disease patterns were identified: 83 countries, primarily in the Middle East, Southeast Asia, the USA, and Russia, showed a high and rapidly increasing pattern, whereas 33 countries, mainly small island developing states, had the highest incidence rates but relatively stable trends. Three risk profiles were also observed, with Western dietary risks and high LDL cholesterol more prevalent in higher-SDI countries, while high sodium intake and low fruit consumption predominated in Asian and African countries. Trend-based projections suggested continued increases in T2DM incidence rates globally, with persistent inequalities.

**Conclusions:**

Postmenopausal women face a substantial and growing T2DM burden, marked by pronounced geographic and socioeconomic inequities and heterogeneous risk patterns. These findings underscore the need to move beyond one-size-fits-all strategies toward tailored prevention and healthcare strategies.

**Supplementary Information:**

The online version contains supplementary material available at https://doi.org/10.1186/s12916-026-05107-0.

## Background

Diabetes is among the fastest growing chronic diseases worldwide and imposes substantial burdens on health systems, economies, and population health [1–3]. According to previous Global Burden of Disease (GBD) studies, diabetes has consistently ranked among the top ten causes of death and disability-adjusted life years (DALYs) globally [4, 5]. The International Diabetes Federation (IDF) estimated that 537 million people were living with diabetes in 2021, with global health expenditures reaching US$966 billion [6]. Type 2 diabetes mellitus (T2DM) represents the predominant form, accounting for 96.0% of cases and 95.4% of DALYs in 2021, making it the primary driver of the global diabetes epidemic [2]. Within this global burden, women experience disproportionately higher adverse outcomes from diabetes, with postmenopausal women particularly vulnerable owing to the combined effects of aging and menopausal endocrine changes [7, 8].

Menopause is a pivotal turning point in women’s cardiometabolic risk. During the menopausal transition, adverse shifts in insulin sensitivity, central adiposity, dyslipidemia, and vascular function commonly occur, collectively contributing to heightened risks of T2D [9, 10]. In 2020, the American Heart Association (AHA) recognized the menopausal transition as a sex-specific life stage that significantly influences long-term cardiometabolic health [11]. Despite this recognition, current health systems continue to under-recognize and under-serve the needs of this population, and postmenopausal women with diabetes remain under-represented in clinical research, which limits the evidence base for targeted prevention and treatment [12]. The urgency of addressing this gap is underscored by demographic trends: roughly one quarter of women worldwide were aged 50 years or older, a proportion which projected to increase [13].

The GBD study has provided comprehensive data of the global landscape of diabetes and previous studies have offered systematic analysis of diabetes burden in different populations, including adolescents, young adults, and elderly people (aged 65+) [14–16]. These studies have documented higher diabetes burdens in lower-income settings and identified metabolic risks as leading contributors at the overall population level, yet the burden, temporal trajectories, geographic inequities, and risk-factor profiles specific to postmenopausal women remain poorly characterized. To address this critical gap, we present a comprehensive, comparative assessment of the global epidemiology of T2DM in this population. Our study aims to provide insights to guide targeted prevention, optimize screening protocols, and inform equitable policy and resource allocation for postmenopausal women worldwide, thereby addressing the imperative to prioritize this vulnerable and growing population in diabetes prevention and management.

## Methods

### Overview

GBD 2021 is a systemetic global health initiative to estimate the burden of 371 diseases and 88 attributable risk factors across 204 countries and territories. Detailed estimation methodologies and data sources were reported in previous publications by the GBD 2021 collaborators [4, 17]. In this study, we used an age-based proxy of ≥ 55 years to define postmenopausal status. This cut-off represents a conversative threshold by which the vast majority of women have completed menopausal transition, and remains consistent with broader literature [18, 19]. Our primary outcomes were the incidence rate (IR) and mortality rate (MR) of T2DM among postmenopausal women globally, and across different regions and countries/territories. Within the GBD framework, incident T2DM refers to newly occurring cases within a given year, and T2DM mortality refers to deaths for which diabetes was assigned as the underlying cause of death based on vital registration and verbal autopsy data, modeled to generate comparable cause-specific mortality estimates across locations and years. T2DM is defined as fasting plasma glucose equal or greater than 7 mmol/L, with ICD-10 code of E11 or ICD-9 code of 250. The summary exposure values (SEVs) represent a population’s weighted exposure to a given risk factor, accounting for both the prevalence and severity of exposure levels in relation to disease burden. The Socio-demographic Index (SDI) is a composite measure that reflects the socioeconomic development level of a location, with higher values indicating higher socioeconomic development levels. SDI is calculated based on the total fertility rate among women younger than 15 hears, educational attainment for individuals aged 15 years and above, and per capita income adjusted for time lag. To further characterize age-related heterogeneity within the study population, we compared T2DM incidence and mortality rates across 5-year age groups from 55 to 59 to 90–94 years and the ≥ 95 years age group, globally and across SDI regions in 2021. All data are publicly available through the online query tool Global Health Data Exchange (GHDx) (https://vizhub.healthdata.org/gbd-results/*).* Our study followed the Guidelines for Accurate and Transparent Health Estimates Reporting Guidelines for cross-sectional studies (GATHER) [20]. All data analysis, visualizations were performed with RStudio (v 4.4).

### Temporal trends and disease trajectory patterns analysis

We quantified temporal trends in country-level IRs and MRs for T2DM among postmenopausal women across 204 countries and territories, from 1990 to 2021, using the estimated annual percent change (EAPC). For each country and outcome, we fitted a log-linear model of the form $\mathrm{ln}\left(rate\right)=\alpha+{B}_{1}t+\epsilon$, assuming a constant proportional change over time. Trends were interpreted as increasing when the 95% CI was entirely > 0, decreasing when entirely < 0, and as stable otherwise. Statistical significance was evaluated at a two-sided α = 0.05.

To characterize cross-national patterns in the burden of T2DM among postmenopausal women and to group countries with similar levels and temporal trends, we applied K-means clustering to four indicators per country: IR and MR from the most recent year (2021), and the EAPCs for IR and MR from 1990 to 2021. All variables were standardized to zero mean and unit variance to ensure equal contribution to the clustering. K-means clustering was performed using Euclidean distance with multiple random starts to reduce sensitivity to local minima. The optimal number of clusters (K) was determined by jointly inspecting the elbow criterion (within-cluster sum of squares) and the average silhouette width, favoring the smallest K at the elbow that also maximized silhouette performance (Additional file 1: Fig. S1).

### Health disparity and health inequality analysis

We quantified cross-national inequality in T2DM burden using the concentration index (CI), ranking countries and territories by SDI [21]. For each location *i*, let ${y}_{i}$ denote the IR or MR among postmenopausal women, ${w}_{i}$ the share of the relevant female population, and ${R}_{i}$ the fractional rank of location *i* in the SDI distribution (from lowest to highest), computed with population weights. The CI was calculated with the grouped-data formula

$$CI=\frac{2}{\mu}\sum_{i}{w}_{i}{y}_{i}({R}_{i}-\frac{1}{2})$$

where $\mu={\sum}_{i}{w}_{i}{y}_{i}$ is the population-weighted mean rate. CI ranges from − 1 to + 1; values below 0 indicate a disproportionate concentration of the adverse outcome in lower-SDI settings, values above 0 indicate concentration in higher-SDI settings, and 0 denotes equality.

### Population-level correlates and exposure profiles with T2DM burden

We employed a data-driven risk assessment approach utilizing machine learning algorithms and regression analysis to identify significant population-level exposures with incident T2DM among postmenopausal women globally. This analysis was distinct from the comparative risk assessment framework used in GBD 2021 and was intended for hypothesis generation, exposure prioritization, and characterization of cross-country patterns [22, 23]. First, we excluded risk exposures not biologically or behaviorally plausible for T2DM in women aged ≥ 55 years (e.g., occupational ergonomic factors, unsafe sex, child growth failure indicators) and retained factors supported by prior evidence from systematic reviews and meta-analyses as being associated with T2D risk. This yielded 32 candidate factors spanning metabolic, dietary, behavioral, and environmental domains (Additional file 1: Table 1). All candidate risks were entered into two supervised machine-learning models, Random Forest and XG Boost, with the country-level IR of T2DM among postmenopausal women as the outcome. Hyperparameters were tuned via 5-fold cross-validation repeated 10 times, selecting the combination that minimized out-of-sample root mean square error (RMSE). Model performance was evaluated using out-of-sample RMSE, mean absolute error (MAE), and R². Feature importance was quantified using Shapley Additive Explanations (SHAP) values, which decompose each prediction into additive contributions from individual features. Global importance was summarized as the mean absolute SHAP value across countries.

Risk exposures with consistent, non-negligible SHAP contributions between the two models were entered into a negative binomial regression model of incident case counts to accommodate overdispersion:

$$\begin{array}{l}{C}_{i}\sim{NB}\left({\mu}_{i},\theta\right),\\{\mathrm{log}}\left({\mu}_{i}\right)=\alpha+\mathop{\sum}\limits_k {\beta}_{k}{X}_{ik}+\mathrm{l}\mathrm{o}\mathrm{g}\left(P{Y}_{i}\right)\end{array}$$

where ${C}_{i}$ is the number of incident cases in country *i*, ${X}_{ik}$ are standardized exposures, $P{Y}_{i}$ denotes female person-years (offset), and $\mathrm{e}\mathrm{x}\mathrm{p}\left({\beta}_{k}\right)$ yields the relative risk (RR) per 1-SD increase in exposure. We report RRs with 95% confidence intervals using cluster-robust standard errors by country. Multicollinearity was assessed (variance inflation factors), and highly collinear predictors were not entered simultaneously. Statistical significance was defined as two-sided *P* < 0.05 with Benjamini–Hochberg false discovery rate control across tested predictors.

To identify countries with similar population-level exposure profiles, we constructed a country × factor exposure matrix using the final set of T2DM-related exposures retained from the preceding analyses. For each factor, we used the corresponding SEV, applied log transformation where distributions were right-skewed, and standardized values within each factor to z-scores to ensure comparability across units. We then performed hierarchical agglomerative clustering on countries using Euclidean distance and Ward’s linkage, which minimizes within-cluster variance while allowing heterogeneous cluster sizes (Additional File 1: Fig. S2). The number of country clusters was chosen by inspecting the dendrogram together with the average silhouette width and the gap statistic. We selected the smallest cut height that maximized these criteria and yielded interpretable groups. Internal validity was assessed using the cophenetic correlation and bootstrap resampling.

To quantify the overall risk exposure, a composite risk index was constructed with the included risk factors. For each factor, country-specific exposure levels were ranked and categorized into quartiles, with scores assigned from 1 (lowest quartile) to 4 (highest quartile). The cumulative exposure score for each country was then calculated by summing the quartile scores across all risk factors, with higher values indicating greater overall exposure.

We additionally conducted a post-hoc consistency analysis for the primary descriptive outcomes. This analysis was limited to T2DM incidence and mortality rates and EAPCs and was not used to update the main GBD 2021-based modelling framework. Specifically, we compared country-level incidence and mortality rates in 2021 with corresponding estimates in 2022 and 2023, and compared EAPCs calculated for 1990–2021 with EAPCs extended through 2022 and 2023. Concordance was assessed using Spearman rank correlations and scatter plots with identity lines. 

### Projections and forecasts to 2040

We generated trend-based projections of T2DM incidence and mortality rates among women aged ≥ 55 years through 2040 using exponential smoothing state-space models. These models were used to extend historical 1990–2021 trajectories under the assumption that past temporal patterns continue, rather than to predict future burden under changing policy, treatment, diagnostic, or prevention conditions. Model selection was conducted using likelihood-based information criteria, and the final model for each series (global, SDI region) was chosen as the best-fitting parsimonious specification with the lowest Akaike information criterion (AIC) and Bayesian information criterion (BIC). Model adequacy was assessed using residual diagnostics, including visual inspection of residual plots and the Ljung–Box test to evaluate residual autocorrelation. Detailed results regarding our model selection and diagnostics are summarized in Additional file 1, Table 2.

## Results

**Fig. 1:**
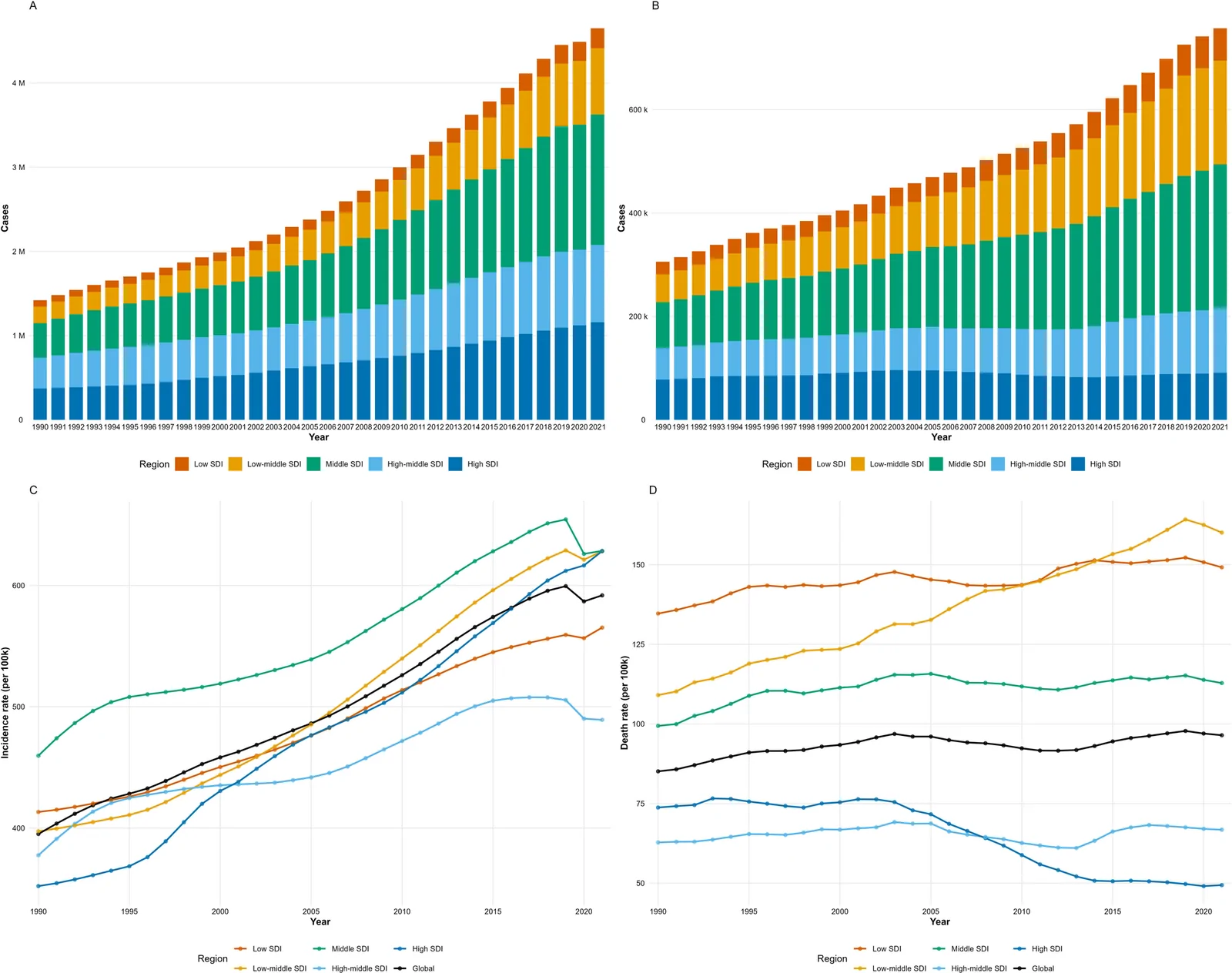
(**A**) Incident cases and (**B**) Death cases of T2DM among postmenopausal women across five SDI regions globally, 1990–2021; (**C**) Incidence rate and (**D**) Mortality rate of T2DM among postmenopausal women globally and across five SDI regions, 1990–2021

In 2021, an estimated 758,000 (95% UI 691,000–813,000) deaths and 4.65 (95% UI 4.00–5.38) million incident cases of T2DM occurred among postmenopausal women worldwide (Fig. 1; Table 1). From 1990 to 2021, the IR rose consistently across all SDI regions, reaching 591.88 (95% UI 508.71–684.00) per 100,000 women globally in 2021 (EAPC: 1.40%, 1.35%-1.44%). By contrast, the MR showed divergent trends across SDI levels, with a global MR of 96.44 (95% UI 87.86–103.35) deaths per 100,000 women in 2021 (EAPC: 0.28%, 0.19–0.37). Across global and SDI regions, T2DM incidence rates were highest in the youngest age groups within the study population, generally peaking at ages 55–59 or 60–64 years before declining at older ages. In contrast, T2DM mortality rates increased progressively with age and reached their highest levels in the ≥ 95 years age group across global and SDI regions (Additional file 1: Fig. S3-S4).

**Table 1:** Incidence rate, mortality rate of type 2 diabetes mellitus among postmenopausal women in 2021 and estimated annual percentage changes (EAPC) from 1990–2021 across global regions

	Incident		Mortality	
	Incidence rate (per 100,000 population), 2021	EAPC (%) in incidence rates, 1990–2021	Mortality rate (per 100,000 population), 2021	EAPC (%) in mortality rates, 1990–2021
Global	591.88 (508.71 to 684.00)	1.40 (1.35 to 1.44)	96.44 (87.86 to 103.35)	0.28 (0.19 to 0.37)
SDI regions				
High SDI	628.33 (551.41 to 711.39)	2.01 (1.94 to 2.07)	49.38 (40.96 to 53.73)	-1.73 (-1.99 to -1.48)
High-middle SDI	489.22 (415.25 to 571.03)	0.84 (0.75 to 0.93)	66.77 (58.43 to 73.59)	0.08 (-0.06 to 0.21)
Middle SDI	628.56 (535.29 to 740.28)	1.07 (0.99 to 1.15)	112.83 (103.03 to 121.13)	0.31 (0.2 to 0.41)
Low-middle SDI	628.21 (537.17 to 727.55)	1.72 (1.65 to 1.79)	160.07 (145.51 to 175.31)	1.33 (1.29 to 1.37)
Low SDI	565.30 (489.36 to 654.69)	1.14 (1.10 to 1.18)	149.17 (133.06 to 168.49)	0.31 (0.25 to 0.36)
GBD regions				
Andean Latin America	774.32 (675.40 to 873.23)	1.85 (1.71 to 1.99)	131.58 (110.82 to 158.10)	0.42 (0.23 to 0.61)
Australasia	439.60 (375.81 to 507.68)	1.58 (1.53 to 1.63)	45.25 (36.25 to 51.10)	-1.14 (-1.50 to -0.77)
Caribbean	833.05 (701.56 to 973.58)	0.49 (0.44 to 0.54)	205.45 (177.45 to 233.84)	-0.97 (-1.05 to -0.89)
Central Asia	576.69 (450.92 to 661.20)	3.02 (2.92 to 3.12)	80.87 (71.90 to 90.21)	1.43 (0.93 to 1.93)
Central Europe	621.53 (539.32 to 712.90)	0.92 (0.80 to 1.03)	81.70 (72.91 to 88.56)	0.70 (0.54 to 0.85)
Central Latin America	774.07 (666.28 to 905.27)	-0.22 (-0.34 to -0.10)	231.10 (203.56 to 256.40)	-0.59 (-0.79 to -0.38)
Central Sub-Saharan Africa	554.48 (460.27 to 651.23)	1.15 (1.10 to 1.19)	179.08 (132.10 to 228.15)	0.33 (0.21 to 0.46)
East Asia	390.03 (302.76 to 484.92)	0.26 (0.03 to 0.49)	45.02 (36.41 to 54.79)	-0.58 (-0.92 to -0.24)
Eastern Europe	404.38 (338.03 to 483.79)	1.74 (1.69 to 1.79)	77.53 (70.00 to 83.73)	3.03 (1.65 to 4.42)
Eastern Sub-Saharan Africa	506.98 (453.58 to 563.82)	0.44 (0.38 to 0.51)	148.52 (129.20 to 171.76)	-0.62 (-0.70 to -0.53)
High-income Asia Pacific	604.12 (521.84 to 703.39)	0.92 (0.76 to 1.07)	25.92 (19.50 to 30.03)	-1.71 (-1.94 to -1.49)
High-income North America	897.82 (790.90 to 1009.28)	3.84 (3.56 to 4.11)	52.08 (44.79 to 56.41)	-2.33 (-2.90 to -1.76)
North Africa and Middle East	965.14 (814.26 to 1140.66)	2.12 (2.07 to 2.16)	140.93 (125.01 to 158.56)	1.13 (0.96 to 1.31)
Oceania	863.44 (717.06 to 1030.32)	0.95 (0.87 to 1.04)	416.14 (355.50 to 486.80)	0.67 (0.48 to 0.85)
South Asia	481.04 (401.63 to 563.92)	1.27 (1.18 to 1.36)	148.49 (130.35 to 168.33)	1.52 (1.38 to 1.66)
Southeast Asia	1034.35 (910.73 to 1171.96)	2.18 (2.06 to 2.29)	154.87 (137.26 to 175.07)	0.32 (0.18 to 0.46)
Southern Latin America	763.50 (631.98 to 900.10)	1.44 (1.38 to 1.50)	72.53 (64.92 to 78.72)	-1.25 (-1.52 to -0.98)
Southern Sub-Saharan Africa	1232.08 (1093.51 to 1390.57)	2.33 (2.23 to 2.43)	382.84 (354.81 to 412.03)	2.23 (1.69 to 2.77)
Tropical Latin America	699.20 (580.62 to 841.59)	0.45 (0.33 to 0.56)	136.66 (120.12 to 146.58)	-0.62 (-0.79 to -0.45)
Western Europe	432.96 (377.17 to 501.47)	0.46 (0.41 to 0.50)	56.81 (44.77 to 63.47)	-1.57 (-1.67 to -1.47)
Western Sub-Saharan Africa	718.08 (626.26 to 819.69)	1.53 (1.48 to 1.59)	150.19 (127.08 to 178.83)	0.35 (0.19 to 0.50)

**Fig. 2:**
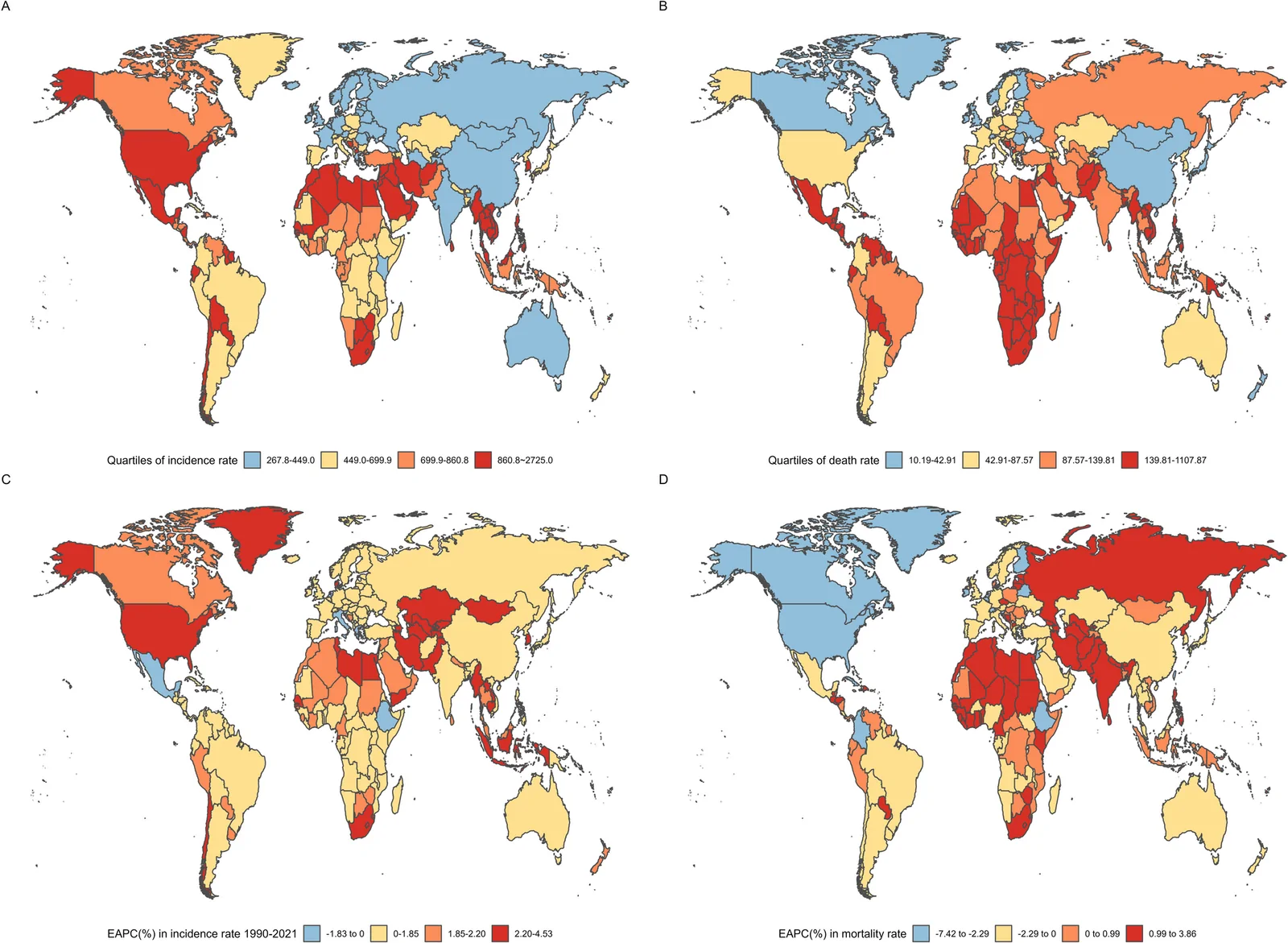
Geographic distribution and temporal trends of type 2 diabetes mellitus among postmenopausal women across 204 countries and territories. **A** Incidence rates in 2021; (**B**) mortality rates in 2021; (**C**) EAPCs s in incidence rates from 1990 to 2021; and (**D**) EAPCs in mortality rates from 1990 to 2021

Significant geographical disparities were observed in the disease burden of T2DM among postmenopausal women globally (Fig. 2; Table 1). In 2021, Sub-Saharan Africa, North Africa and the Middle East, Southeast Asia, and North America recorded the highest IRs, with Qatar, Bahrain, and Sri Lanka among the highest at the country level. Mortality rates were greatest in low-SDI settings, led by Fiji, the Marshall Islands, and Kiribati. Regarding temporal trends, several countries in Central Asia, Southeast Asia, and Oceania experienced the steepest IR increases. Uzbekistan, the United States, and Egypt showed the fastest rises, with estimated annual percent changes (EAPCs) of approximately 4%. For MR, the largest increases were concentrated in many African countries and in South and Central Asia; Russia, Cabo Verde, and Lesotho reported the greatest EAPCs, exceeding 3.5%. Region-specific and country-specific estimates are provided in Table 1 (Additional file 1, Table 3).

**Fig. 3:**
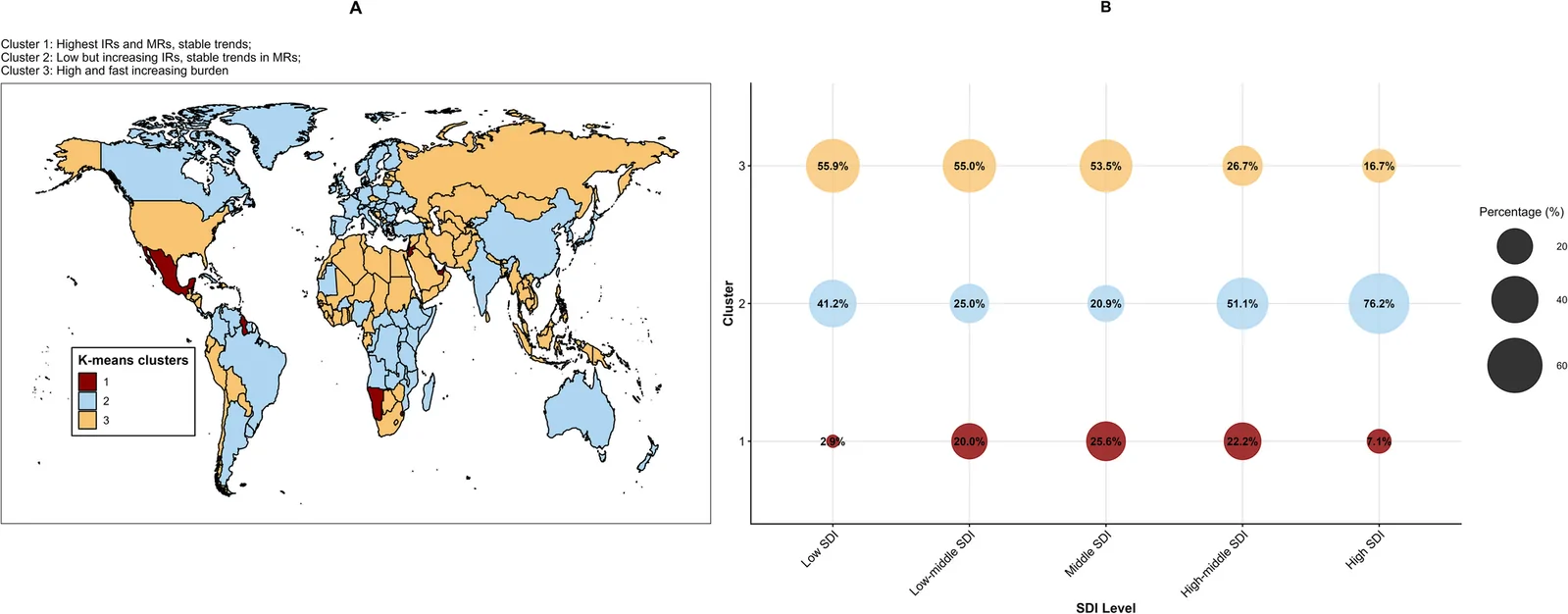
Disease-burden trajectory clusters of type 2 diabetes mellitus among women aged ≥ 55 years. **A** K-means clustering of countries based on 2021 incidence and mortality rates and estimated annual percentage changes from 1990 to 2021; (**B**) distribution of trajectory clusters across SDI regions

Across 204 countries and territories, K-means clustering of burden profiles and trajectories identified three distinct patterns (Fig. 3). **Cluster 1** (*n* = 33; 16.2%) comprises settings with the highest incidence and mortality in 2021 but largely stable temporal trends. Most are small island developing states (SIDS) in the Pacific and Caribbean, such as the Cook Islands, Tonga, Barbados, Haiti, and Trinidad and Tobago, where small populations and entrenched cardiometabolic risks may sustain high baseline rates. **Cluster 2** (*n* = 88; 43.1%) includes settings with moderate rates in 2021 but steadily rising incidence; this pattern is most common in high- and high-middle SDI settings, particularly across Europe. **Cluster 3** (*n* = 83; 40.7%) reflects a dual burden of high baseline rates coupled with the fastest increases over time. It is concentrated in North Africa, Central Asia, Southeast Asia, and Oceania regions, also the United States (US) and Russia.

**Fig. 4:**
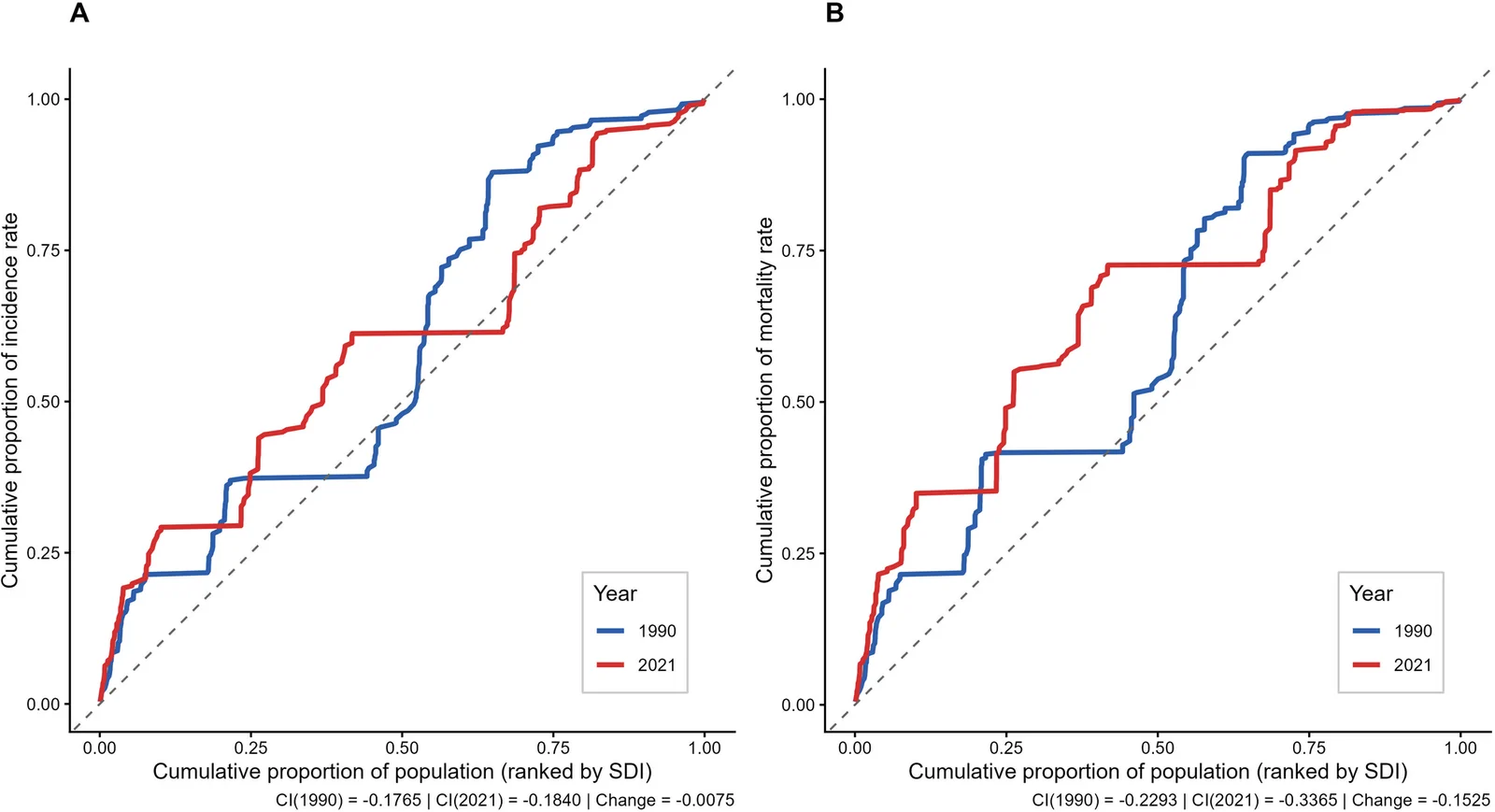
Cross-national inequalities in type 2 diabetes mellitus burden among women aged ≥ 55 years. Concentration indices for (**A**) incidence rates and (**B**) mortality rates according to Socio-demographic Index in 1990 and 2021

Health inequality analyses demonstrated a disproportionate burden borne by lower-SDI countries (Fig. 4). The concentration index for incidence became more negative from, −0.177 in 1990 to −0.184 in 2021, while the CI for mortality changed from −0.229 to −0.337 over the same period, indicating that both incidence and, more markedly, mortality have increasingly concentrated among lower-SDI settings.

**Fig. 5:**
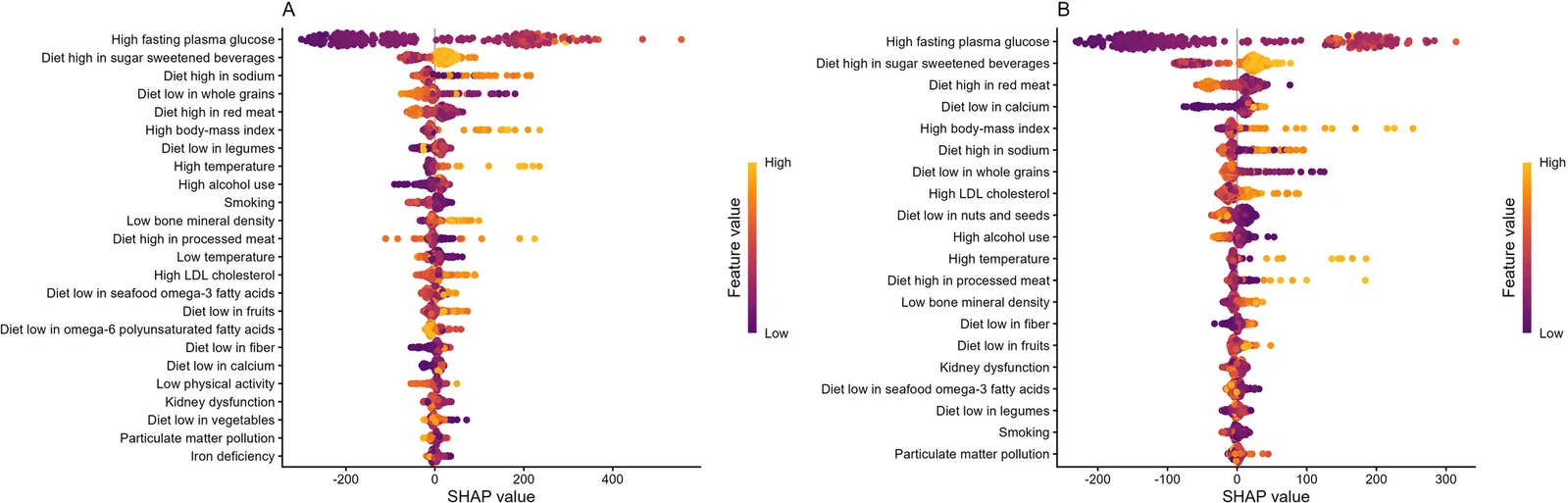
SHAP summary plot of risk factors with T2DM among postmenopausal women with (**A**) XG Boost and (**B**) Random Forest methods

We prioritized country-level exposure indicators correlated with T2DM incidence using SHAP values from Random Forest and XGBoost models trained on 32 candidate exposure indicators (Fig. 5). Both models showed good predictive performance and produced broadly concordant exposure rankings (Additional file 1: Fig. S5). Because marginal gains in model contribution plateaued beyond the top 20 ranked indicators, these indicators were carried forward into multivariable negative binomial regression. Nine population-level exposures showed positive ecological associations with T2DM incidence after multivariable adjustment, including high fasting plasma glucose (FPG), high body-mass index (BMI), low bone mineral density (BMD), high LDL-cholesterol (LDL-C), diet high in sodium, diet high in sugar-sweetened beverages, diet low in fruits, diet high in processed meat and higher ambient temperature (Table 2). Metabolic factors had the highest RRs, led by high FPG (RR 1.29; 95% CI: 1.21–1.37), high BMI (1.14, 95% CI: 1.11–1.18) and low BMD (1.13, 95% CI: 1.03–1.24). Dietary exposures were associated with modest but significant elevations, with RR ranging from 1.05 in diet low in fruits to 1.08 from diet high in sodium. High ambient temperature was likewise associated with increased incidence (RR 1.03; 95% CI 1.01–1.05).

**Table 2:** Relative risks (RR) of risk exposures associated with T2DM incidence among postmenopausal women across 204 countries and territories

Risk Factor	RR	95% Confidence Interval	*P*-value
High fasting plasma glucose	1.29	(1.21, 1.37)	<0.001
High body-mass index	1.14	(1.11, 1.18)	<0.001
Low bone mineral density	1.13	(1.03, 1.24)	<0.001
High LDL cholesterol	1.09	(1.03, 1.16)	<0.001
Diet high in sodium	1.08	(1.04, 1.13)	<0.001
Diet high in sugar-sweetened beverages	1.08	(1.03. 1.12)	<0.001
Diet high in processed meat	1.07	(1.03, 1.11)	<0.001
Diet low in fruits	1.05	(1.01, 1.10)	<0.001
High temperature	1.03	(1.01, 1.05)	<0.001

**Fig. 6:**
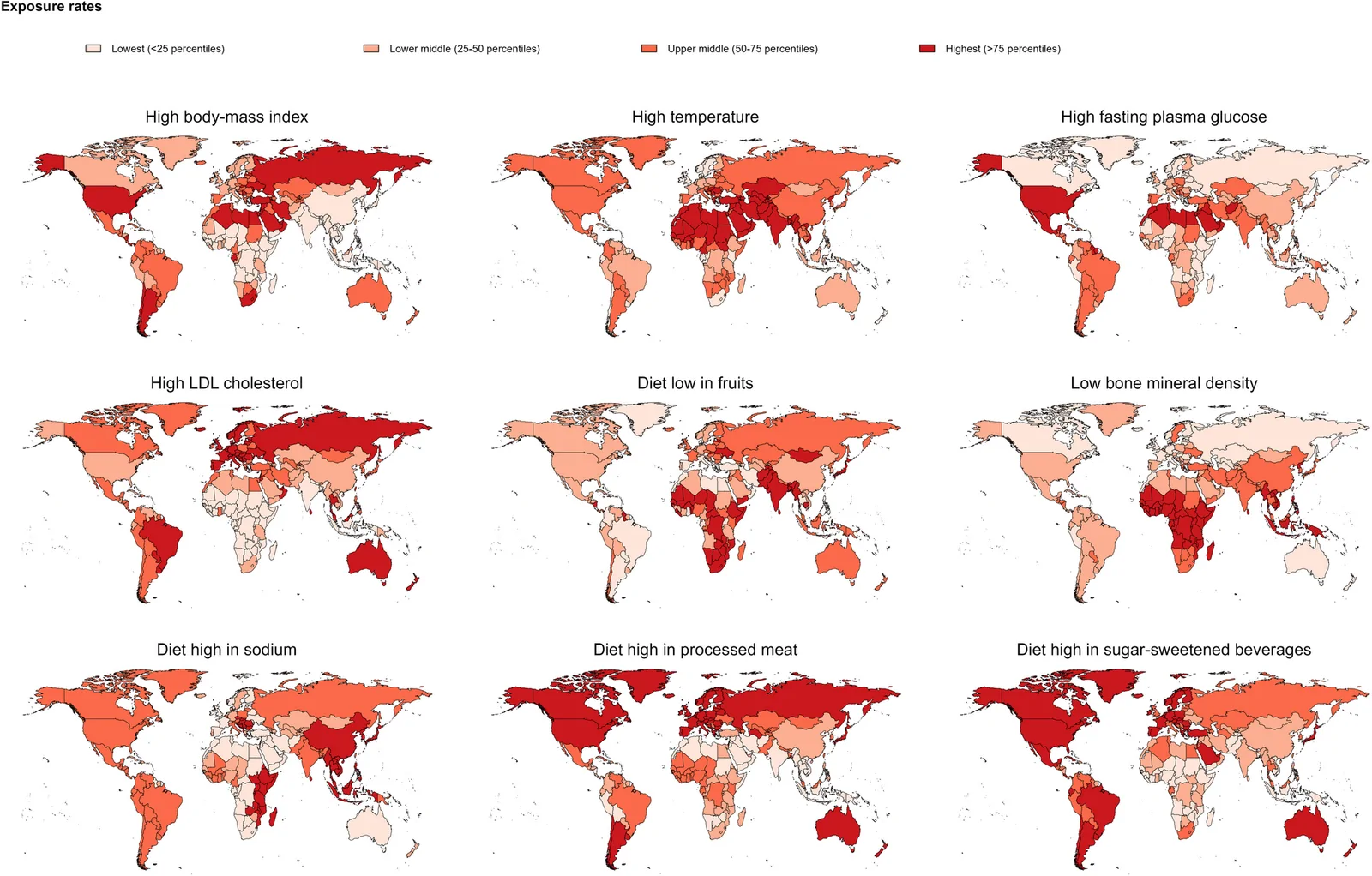
Global distribution of country-level exposure indicators correlated with type 2 diabetes mellitus incidence among women aged ≥ 55 years, categorized by quartiles

The geographic distribution of these population-level exposure indicators varied substantially (Fig. 6). The United States and several countries in the Middle East, North Africa, and Southern Latin America showed the highest exposures to both high BMI, high FPG while Southern America and Europe had the greatest exposure to high LDL-C. Europe, North America, and Australasia also had particularly elevated exposures to diets high in processed meat, and sugar-sweetened beverages, reflecting a predominantly Western dietary pattern. In contrast, many countries in Asia and Sub-Saharan Africa had comparatively lower exposures to these metabolic and Western dietary risks but experienced higher exposures of low BMD and diets low in fruits. High sodium intake was especially pronounced in East and South Asia, parts of the Middle East, and Eastern Europe. Exposure to high ambient temperature was greatest in North Africa, the Middle East and South Asia, overlapping with several of the regions already facing substantial metabolic risks.

**Fig. 7:**
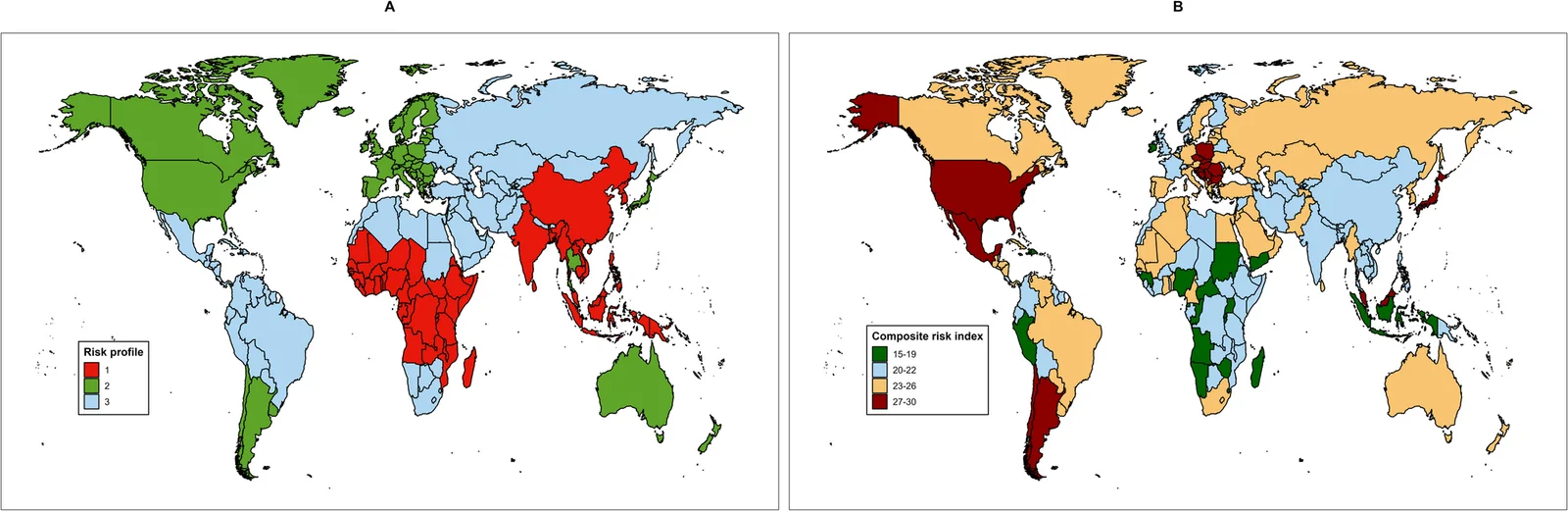
**A** Population-level exposure profiles of T2DM burden among postmenopausal women with hierarchical clustering. **B** Composite risk exposures of population-level exposures associated with T2DM incidence among postmenopausal women globally

Hierarchical clustering of exposures to the nine associated risk exposures identified three distinct, geographically coherent profiles (Fig. 7). First, countries across North Africa, the Middle East, Central Asia, Eastern Europe, and southern Latin America clustered together, showing the highest exposures to metabolic risks, including high FPG, high BMI, and high LDL-C. Second, high-income settings such as North America, Australasia, and much of Europe exhibited a profile characterized by Western dietary patterns and high LDL-C. Third, many Sub-Saharan African and Asian countries displayed a different profile marked by low BMD, high sodium intake, and low fruit consumption, alongside comparatively lower metabolic risk exposures. Overall, composite risk exposure levels were higher in high-SDI countries, whereas many African and Asian countries tended to fall into the lower-exposure profile.

**Fig. 8:**
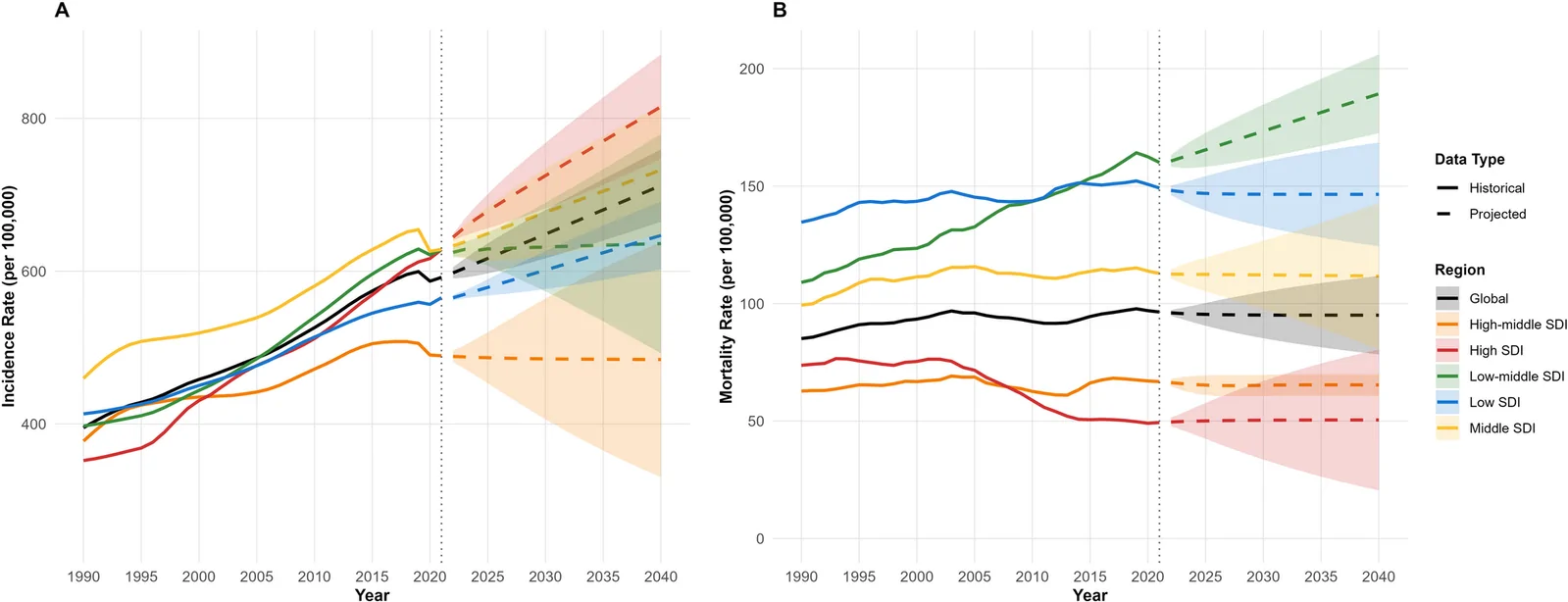
Trend-based projected trajectories of the (**A**) incidence rate and (**B**) mortality rates of T2DM burden among postmenopausal women through 2040

Our forecasting models suggested that the incidence rate of T2DM among women aged ≥ 55 years would continue to increase through 2040, whereas mortality rates would remain comparatively stable at the global level (Fig. 8). By 2040, the projected global incidence and mortality rates were 712.08 (95% CI: 664.74–759.42) and 95.07 (95% CI: 78.36–111.78) per 100,000 women, respectively. Projected incidence trajectories increased across most SDI regions, with high-SDI regions showing the highest projected incidence rates. In contrast, low-middle SDI regions showed increasing projected mortality trajectories, whereas high-SDI regions retained the lowest projected mortality rates through 2040.

In the post-hoc analysis, the primary descriptive patterns were highly stable when compared with updated estimates extending through 2022 and 2023. Country-level incidence and mortality rates in 2022 and 2023 were strongly correlated with 2021 rates (spearman r>0.95). EAPCs extended through 2022 and 2023 were also highly concordant with the main 1990–2021 EAPCs. These secondary results support the validity of the primary findings and modelling in our study. Detailed estimates and comparison results are reported in Additional file, Fig. S6–S7, Tables 4, and 5.

## Discussion

This study provides a comprehensive assessment of the burden and risk profiles of T2DM among postmenopausal women globally. Overall, we found a rising, yet persistent inequal burden of T2DM among postmenopausal women globally. The incidence rate of T2DM showed substantially increase, yet the mortality burden was disproportionately concentrated in low and low-middle SDI settings. Epidemiologic and risk profiles also varied substantially: the fastest increases occurred in North Africa, the Middle East, and parts of Central and Southeast Asia, whereas high-income countries shared a risk profile dominated by Western dietary and metabolic factors but displayed divergent national trends. Sub-Saharan African and several Asian countries showed more modest increases yet were characterized by risk patterns driven by low BMD and suboptimal dietary quality. Overall, our findings highlight substantial heterogeneity in the epidemiology and risk landscape of T2DM in postmenopausal women and underscore the need for prevention and policy strategies tailored to local contexts.

Our findings on the global burden of T2DM are consistent with the marked health disparities observed in other non-communicable diseases, such as cardiovascular diseases, among postmenopausal women [18, 24]. The burden is disproportionately high in lower SDI regions, with Sub-Saharan Africa, Central Asia, South Asia, Southeast Asia, and Oceania experiencing the highest T2DM-attributable mortality. Furthermore, we identified a unique pattern across 33 countries in the Caribbean, Oceania, and the Middle East. These nations exhibited the highest global incidence and mortality rates, yet with stable trends, which highlights persistent gaps in healthcare systems. Women in these settings confront a compromised healthcare infrastructure compounded by profound structural barriers [25, 26]. System-level failures are evident in data from 55 nationally representative surveys in low- and middle-income countries (LMICs), which indicate that only 4.6% of individuals with diabetes receive all recommended treatments [27]. Similarly, 77% of people in LMICs have unmet diabetes care needs, with health systems experiencing significant patient loss at the diagnosis and control stages [28]. These systemic challenges are exacerbated by structural barriers such as restrictive gender norms, mobility constraints, low health literacy, and financial hardship, which collectively delay care and impede self-management [29, 30]. Collectively, these multifaceted factors directly contribute to the heightened health disparities, particularly the increased health inequalities of mortality from T2DM, underscoring an urgent need for global health initiatives that prioritize equity and target the specific constraints of low-resource settings.

Although high-burden and fast-rising patterns of T2DM among postmenopausal women were most common in low- and lower-middle-SDI countries, similar trajectories were also observed in two higher-SDI settings, the US and Russia. In the US, postmenopausal women ranked in the top quartile both incidence rate and annual increase, whereas Russia showed one of the fastest increases in T2DM-related mortality. The likely drivers in these settings differ from those in LMICs and reflect the confluence of intense metabolic risk accumulation, epidemiological shifts in aging and multimorbidity, and fragmentation of healthcare delivery. U.S. National Center for Health Statistics data indicate severe obesity in 14.7% of women aged 40–59 years and 8.4% of women over 60 years, roughly twice the prevalence in men [31]. Similarly, in Russia, more than 80% of women aged 55–64 are overweight or obese [32]. These burdens are socially patterned, with higher obesity among women of lower socioeconomic status [33, 34]. A previous systematic review reported approximately 40% prevalence of multi-morbidities among aging populations in North America and Europe, while projection studies also indicated a nearly doubled increase with the population over 50 years by 2050 in the U.S [35, 36]. The persistent obesogenic environment, compounded by the increasing burden of multimorbidity, necessitates effective and integrative healthcare systems, yet current healthcare services and delivery remain fragmented [37]. In the U.S., fragmentation in health policy and governance, payers and reimbursement design, and service delivery contributes to low accountability and coordination [38]. Similarly, the Russian health system faces critical gaps, as highlighted by the NATION study, which found that over 50% of Russian adults with diabetes were undiagnosed, alongside issues of insufficient access to essential supplies and stark urban-rural disparities in care quality [39, 40]. These findings suggest that the burden of T2DM among postmenopausal women is highly context-specific, extending beyond traditionally low-resource settings, and should be recognized as an important global public health priority.

The population-level exposure profiles provide ecological context for the heterogeneous burden of T2DM among women of postmenopausal age. Countries with higher T2DM incidence tended to have greater exposure to metabolic risks. Dysglycemia and adiposity are established drivers of T2DM, and their effects may be amplified after menopause as declining estrogen levels promote central fat redistribution, insulin resistance, dyslipidemia, and vascular dysfunction [41, 42]. Population-based studies have indicated indicates that BMI, and other adiposity indices carry stronger relative risks for T2DM in women than in men [43, 44]. Similarly, recent estimates also attribute a large share of incident diabetes to obesity in women (~50%) compared to men (~40%) [45]. Importantly, these risks are unevenly distributed globally, contributing to content-specific patterns of T2DM burden among postmenopausal women. Other metabolic risks, including low BMD, high LDL-cholesterol also emerged as country-level correlates of T2DM burden, but these indicators may reflect broader demographic, metabolic, nutritional, environmental, or healthcare contexts rather than direct causal pathways. High temperature also emerged as a relevant environmental exposure with T2DM burden, that is disproportionately concentrated in the North Africa and Middle East region. Overall, these findings delineate divergent population-level patterns of T2DM burden among women of postmenopausal age and support the need for context-specific prevention and health-system strategies.

Diet and nutrition are central to the global T2DM burden among postmenopausal women, but the dominant dietary risks vary substantially across regions. In our analysis, Western-style dietary risks, including higher exposure to processed meat and sugar-sweetened beverages, were more prominent in high-income settings, whereas low fruit intake and high sodium consumption were more common in many Asian and African countries. These differences suggest that nutrition strategies for postmenopausal women should be adapted to local food systems, cultural dietary practices, and ongoing nutrition transitions. Improving overall dietary quality remains particularly important for ageing female populations. Evidence from the Women’s Health Initiative suggests that greater adherence to Portfolio and Mediterranean dietary patterns is associated with lower incident T2DM risk, whereas hyperinsulinemic and proinflammatory dietary patterns are associated with substantially higher risk [46, 47]. Among women with established T2DM, better diet quality also relates to lower cardiovascular risk, underscoring the dual role of diet in primary and secondary prevention [48]. However, much of this evidence originates from Western populations, leaving a critical knowledge gap regarding how dietary risks and protective patterns manifest within the unique food environments and cultural contexts of Asia and Africa. Future studies should therefore evaluate region-specific dietary patterns among postmenopausal women and inform culturally tailored, equity-oriented nutrition policies. More broadly, biological, reproductive, and psychosocial factors that are particularly relevant to women, including polycystic ovary syndrome, gestational diabetes, shorter reproductive lifespan, depression, anxiety, and chronic stress, should be integrated into global T2DM prevention and care frameworks. Expanding research on these women-specific and life-course risk factors across diverse geographic and socioeconomic settings is essential for developing more effective and equitable strategies for postmenopausal women worldwide.

Despite the heightened burden of T2DM among postmenopausal women, significant gaps in diabetes care persist. Women, particularly those in older age groups and lower socioeconomic status encounter lower screening and treatment rates, poorer glycemic control, and greater barriers to accessing healthcare than men [49, 50]. Concurrently, current guidelines often lack sex-specific recommendations tailored to women’s unique risk profiles across the life course, including the menopausal transition [8, 51]. Therefore, policy and research agendas should prioritize equitable access to high-quality diabetes prevention and care for midlife and older women, strengthen professional training on age-specific and sex-specific management, and invest in early-life and reproductive-age prevention strategies focused on lifestyle modification and cardiometabolic risk optimization. Finally, more target-specific research, including intervention trials and implementation studies in diverse settings, is urgently needed to generate evidence-based, women-centered guidance for T2DM prevention and management.

Several limitations should be acknowledged. First, the primary analytic framework was developed using GBD 2021 estimates and therefore extended through 2021. To address the availability of updated global estimates, we conducted a post-hoc consistency analysis for the primary descriptive outcomes using estimates extending through 2022 and 2023. Country-level incidence rates, mortality rates, and EAPCs were highly concordant across releases, supporting the stability of the main descriptive findings, including rising incidence and persistent mortality inequalities. Second, we used women aged ≥ 55 years as an age-based operational proxy for postmenopausal status. Although this conservative threshold likely captures most women after the menopausal transition, menopausal status was not directly measured, and age at natural menopause varies across populations and individuals. Third, given the ecological study design, the associations of risk exposures should be interpreted as population-level associations and not as individual-level associations. The application of machine learning methods were used for exploratory exposure prioritization and pattern recognition. Similarly, the population-level exposure profiles related with T2DM burden should be considered hypothesis-generating and useful for contextualizing regional prevention priorities, rather than confirmatory evidence of causal associations. Finally, our projections results were derived from historical 1990–2021 trajectories and should be interpreted as plausible scenarios under continuation of past temporal patterns rather than as predictions of future disease burden. Future incidence and mortality may differ from these projected trajectories because of changes in prevention, diagnosis, treatment access, population ageing, health-system performance, and broader socioeconomic conditions.

## Conclusions

In conclusion, this study demonstrates a substantial and rising global burden of T2DM among postmenopausal women, accompanied by persistent geographic and socioeconomic disparities. The divergent burden and risk profiles we identified emphasize that these inequities are shaped by heterogeneous epidemiological and social contexts. These findings underscore the need to move beyond uniform strategies toward tailored, evidence-based prevention and management. By guiding context-specific policies and risk-reduction efforts, our work provides a foundation for reducing global inequities and improving outcomes for postmenopausal women worldwide.

## Supplementary Information

Below is the link to the electronic supplementary material.

Supplementary Material 1: Additional File: Supplementary Tables 1–5, Figs. S1–S7.

## Data Availability

All data used in this study was publicly available through the Global Health Data Exchange (https://ghdx.healthdata.org/).

## Abbreviations

GBD: Global Burden of Disease
DALYs: Disability-adjusted life years
T2DM: Type 2 diabetes mellitus
IR: Incidence rate
MR: Mortality rate
EAPC: Estimated annual percent changes
SEV: Summary exposure value
FPG: Fasting plasma glucose
BMI: Body mass index
BMD: Bone mineral density
