# Supplementary material for: Type 2 diabetes burden, inequalities, and epidemiological patterns among postmenopausal women, 1990–2021: a global ecological modelling study
Source: BMC Med. 2026 Aug 11;24:626. doi: 10.1186/s12916-026-05107-0 (PMC13746595; doi:10.1186/s12916-026-05107-0)
Supplement: Supplementary file 1 [file 12916_2026_5107_MOESM1_ESM.docx]

**Supplementary Table 1. Candidate risk exposures screened for inclusion in the machine learning and regression analyses, with definitions and supporting evidence for their association with type 2 diabetes risk.**

| **Risk Exposure** | **Definition** | **Literature Support and Evidence** |
| --- | --- | --- |
| **High fasting plasma glucose** | Fasting plasma glucose (FPG) ≥5.6 mmol/L (100 mg/dL) per ADA criteria, progressing to ≥7.0 mmol/L for T2DM diagnosis (WHO/ADA). Elevated FPG is a direct marker of impaired insulin secretion and peripheral insulin resistance, reflecting β-cell failure to compensate for ongoing metabolic dysregulation. | Individuals with impaired fasting glucose face a 3–6-fold higher T2DM risk vs. normoglycaemic individuals [1]. The Diabetes Prevention Program demonstrated lifestyle and pharmacological interventions targeting FPG significantly delay T2DM onset [1]. |
| **High body-mass index (BMI)** | BMI ≥25 kg/m² (overweight) or ≥30 kg/m² (obese) per WHO criteria. Excess adiposity, particularly visceral fat, promotes insulin resistance through pro-inflammatory adipokine secretion, ectopic lipid deposition, and free fatty acid–mediated impairment of insulin signalling in skeletal muscle and liver. | A meta-analysis of >250 cohort studies confirmed a dose–response association between BMI and T2DM risk; a 5-unit increase in BMI is associated with ~65% greater T2DM risk [2]. Obesity accounts for an estimated 50% of incident T2DM in women [3]. |
| **Kidney dysfunction** | Reduced estimated glomerular filtration rate (eGFR <60 mL/min/1.73 m²) or markers of kidney damage (albuminuria), as modelled in the GBD framework. Impaired renal function promotes systemic insulin resistance through accumulation of uraemic toxins, chronic inflammation, and hormonal dysregulation, while altering renal glucose handling. | Prospective studies document that CKD-related insulin resistance independently predicts incident T2DM; individuals with moderate–severe CKD show significantly impaired peripheral glucose disposal [4]. Bidirectional relationships between CKD and T2DM are well established in longitudinal data. |
| **Low bone mineral density** | Bone mineral density (BMD) below age- and sex-expected norms, typically defined as T-score between −1.0 and −2.5 SD (osteopenia) or <−2.5 SD (osteoporosis) by dual-energy X-ray absorptiometry (DXA). In GBD, low BMD is captured within the osteoporosis risk factor module. | Evidence is bidirectional and complex: some prospective cohorts report higher baseline BMD predicting subsequent T2DM via adiposity confounding, while established T2DM is consistently associated with impaired bone quality and fracture risk independent of BMD [5]. Low BMD may reflect shared metabolic dysregulation relevant to diabetes pathophysiology. |
| **High LDL-cholesterol** | Serum low-density lipoprotein cholesterol (LDL-C) above recommended thresholds (typically >3.4 mmol/L or 130 mg/dL). LDL-C may promote T2DM through cholesterol-mediated impairment of pancreatic β-cell function and systemic inflammation, though its role is partly confounded by statin use and broader dyslipidaemia patterns. | Prospective studies report positive associations between elevated LDL-C and incident T2DM, with a ~10% increased risk per 1 mmol/L increment in some cohorts [6, 7]. |
| **High systolic blood pressure** | Systolic blood pressure (SBP) ≥130 mmHg per ACC/AHA 2017 guidelines. Hypertension and T2DM share pathophysiological mechanisms including endothelial dysfunction, renin–angiotensin–aldosterone system (RAAS) activation, and chronic low-grade inflammation, which collectively impair insulin sensitivity and β-cell function. | A meta-analysis of >4 million adults found each 20 mmHg increment in usual SBP was associated with a 58% higher T2DM risk, independent of BMI and other confounders [8]. RAAS activation directly reduces pancreatic β-cell function and peripheral glucose uptake. |
| **Diet high in red meat** | High habitual consumption of unprocessed red meat (beef, pork, lamb), typically defined as >100 g/day. Heme iron, saturated fatty acids, advanced glycation end-products (AGEs), and branched-chain amino acids in red meat promote insulin resistance, oxidative stress, and pancreatic β-cell damage. | Meta-analyses show ~15–20% increased T2DM risk per 100 g/day increment in unprocessed red meat [9, 10]. Heme iron from red meat is particularly implicated in hepatic oxidative stress and insulin resistance. |
| **Diet high in processed meat** | Regular consumption of cured, smoked, salted, or chemically preserved meat products (e.g., bacon, sausage, hot dogs, deli meats). High sodium, nitrate, and heme iron content, combined with AGEs from processing, contribute to oxidative stress and insulin resistance beyond what is attributable to unprocessed meat. | Meta-analyses consistently show ~30–40% increased T2DM risk per 50 g/day increment in processed meat [9, 10]. This association is stronger than for unprocessed red meat, implicating preservation chemicals, sodium, and nitrosamines rather than meat per se. |
| **Diet high in sugar-sweetened beverages** | Frequent consumption of beverages with added caloric sweeteners, including soft drinks, fruit drinks, sports drinks, and sweetened teas. High fructose content promotes hepatic de novo lipogenesis, visceral adiposity, and hepatic insulin resistance; liquid calories also induce poor satiety response, increasing overall energy intake. | A systematic review and meta-analysis found a 26% higher T2DM risk per additional serving/day of sugar-sweetened beverages [11]. Associations persisted after adjustment for BMI and total energy intake, suggesting direct glycaemic and metabolic effects beyond caloric excess alone. |
| **Diet low in fruits** | Low habitual intake of fresh or minimally processed whole fruits (typically <200–300 g/day). Fruits provide dietary fibre, polyphenols (flavonoids, anthocyanins), vitamin C, and potassium, which improve insulin sensitivity, reduce systemic inflammation, and modulate gut microbiota composition. | Prospective analyses indicate that higher whole fruit intake is inversely associated with T2DM risk; berries, grapes, and apples show the strongest associations, with each 2 additional servings/week reducing T2DM risk by approximately 3–8% [12, 13]. |
| **Diet low in vegetables** | Inadequate intake of non-starchy vegetables (typically <300–400 g/day per WHO recommendations). Vegetables are rich in dietary fibre, antioxidants, magnesium, and bioactive phytochemicals that modulate glucose metabolism, reduce inflammatory markers, and support gut microbiota diversity. | Systematic reviews of prospective studies show inverse associations between vegetable intake and T2DM risk; green leafy vegetable intake is most consistently associated with reduced T2DM incidence, with an estimated 14% risk reduction per 1.35 servings/day increment [13]. |
| **Diet low in whole grains** | Low consumption of intact whole grain cereals and products (whole wheat, oats, brown rice, rye, barley). Whole grains improve postprandial glucose response through fibre-mediated slowing of digestion, contain magnesium and bioactive compounds that improve insulin sensitivity, and reduce systemic inflammation. | A dose–response meta-analysis found 20–30% lower T2DM risk with high vs. low whole grain intake, with a significant inverse trend per 30 g/day increment [14, 13]. |
| **Diet low in milk** | Low consumption of dairy milk and milk-based products. Dairy provides calcium, vitamin D, phosphorus, and bioactive peptides (e.g., insulinotropic casein-derived peptides) that may improve insulin secretion and sensitivity. Full-fat dairy also contains trans-palmitoleate, which has been independently associated with reduced T2DM risk. | A meta-analysis of 22 cohort studies found a 5% lower T2DM risk per 200 g/day increment in total dairy intake, with stronger associations for low-fat dairy and fermented products [15]. |
| **Diet low in fibre** | Total dietary fibre intake below recommended levels (typically <25 g/day for women per WHO/EFSA guidelines). Dietary fibre slows gastric emptying, blunts postprandial glucose and insulin excursions, reduces energy absorption, feeds beneficial gut microbiota, and promotes short-chain fatty acid production that improves metabolic health. | Prospective studies and meta-analyses show 15–20% lower T2DM risk with high vs. low total fibre intake, with a consistent dose–response relationship [16]. Both soluble and insoluble fibre independently contribute to improved glycaemic outcomes. |
| **Diet high in sodium** | High habitual dietary sodium intake, typically >2 g/day (>5 g NaCl/day per WHO upper limit). Excess sodium activates the sympathetic nervous system and RAAS, promotes endothelial dysfunction, and may induce compensatory hyperinsulinaemia through aldosterone-mediated effects on pancreatic insulin signalling. | A systematic review and meta-analysis of 11 observational studies found a 43% higher T2DM risk in the highest vs. lowest sodium intake categories, with a significant linear dose–response trend [17]. |
| **Diet low in calcium** | Low dietary calcium intake below recommended amounts (<700–1000 mg/day). Calcium plays essential roles in insulin secretion from pancreatic β-cells, intracellular insulin signalling, and glucose uptake by skeletal muscle. Calcium also influences vitamin D metabolism, which independently modulates glucose homeostasis. | Prospective cohort studies and pooled analyses report 13–20% lower T2DM risk with adequate vs. insufficient calcium intake, with synergistic effects when combined with vitamin D [18]. Evidence from randomised trials is more heterogeneous. |
| **Diet low in seafood omega-3 fatty acids** | Low intake of long-chain n-3 polyunsaturated fatty acids — eicosapentaenoic acid (EPA) and docosahexaenoic acid (DHA) — from seafood. Marine omega-3s modulate cell membrane phospholipid composition, reduce pro-inflammatory eicosanoid production, and may improve peripheral insulin sensitivity and β-cell function. | A systematic review and meta-analysis found modest inverse associations between seafood omega-3 intake and T2DM risk, particularly in Asian populations [19]. Biomarker-based pooled analyses suggest greater benefit at higher circulating EPA+DHA concentrations. |
| **Diet low in omega-6 polyunsaturated fatty acids** | Low habitual intake of linoleic acid (LA, 18:2n-6), the predominant dietary omega-6 PUFA found in vegetable oils, nuts, and seeds. Replacing dietary saturated fats with linoleic acid improves insulin receptor sensitivity and reduces hepatic lipid accumulation through effects on membrane fluidity and lipid metabolism. | A pooled analysis of biomarker data from >39,000 adults across 20 prospective cohorts found that higher circulating LA was associated with significantly lower T2DM risk (RR 0.65 for highest vs. lowest quintile), suggesting dietary omega-6 adequacy is protective [20]. |
| **Diet high in trans fatty acids** | High intake of industrially produced trans fatty acids (iTFAs), primarily from partially hydrogenated vegetable oils found in processed foods, margarines, and fried products. iTFAs promote systemic inflammation, dyslipidaemia (elevated LDL-C, reduced HDL-C), and impair insulin receptor signalling and β-cell function. | Meta-analyses of cohort studies show 10–30% higher T2DM risk with high vs. low iTFA intake, with the strongest associations for total trans fat and elaidic acid [21]. Unlike ruminant-derived trans fats, industrially produced iTFAs are consistently associated with pro-diabetogenic effects. |
| **Diet low in legumes** | Low consumption of legumes including pulses (beans, lentils, chickpeas, peas) and soybean products. Legumes have a low glycaemic index and are rich in dietary fibre, plant protein, magnesium, and polyphenols that improve postprandial glycaemia and insulin sensitivity. | A prospective analysis from the PREDIMED cohort found a 35% lower T2DM risk in the highest vs. lowest quintile of legume consumption [22]. Systematic reviews and meta-analyses confirm consistent inverse associations between legume intake and T2DM incidence [13]. |
| **Diet low in nuts and seeds** | Low habitual intake of tree nuts (almonds, walnuts, cashews, pistachios), peanuts, and seeds (chia, flax, sunflower). Nuts are rich in unsaturated fatty acids, fibre, magnesium, arginine, and antioxidants (vitamin E, polyphenols) that improve insulin sensitivity, reduce postprandial glycaemia, and lower systemic inflammation. | Meta-analyses report 15–25% lower T2DM risk with frequent nut consumption (≥4 servings/week vs. rare consumption) [23]. Walnuts show particularly consistent associations, potentially due to high alpha-linolenic acid and polyphenol content. |
| **Low physical activity** | Insufficient aerobic and muscle-strengthening activity below WHO-recommended levels (<150 min/week of moderate-intensity or <75 min/week of vigorous-intensity aerobic activity). Physical inactivity reduces GLUT4 expression and glucose transporter translocation in skeletal muscle, impairs mitochondrial biogenesis, and promotes visceral adiposity and insulin resistance. | Walking ≥2.5 hrs/week was associated with a 30% lower T2DM risk in women in the Nurses’ Health Study [24]. Dose–response meta-analyses confirm 25–30% risk reduction with high vs. low physical activity, with benefits apparent even at modest activity levels [3]. |
| **Intimate partner violence** | Physical, sexual, or psychological abuse perpetrated by a current or former intimate partner, as estimated within the GBD violence risk factor module. IPV exposure induces chronic psychological stress, hypothalamic–pituitary–adrenal (HPA) axis dysregulation, elevated cortisol, disrupted sleep, and adverse health behaviours (poor diet, physical inactivity), collectively impairing metabolic health. | A prospective analysis of >65,000 women in the Nurses’ Health Study II found IPV exposure was independently associated with a 42% higher T2DM incidence [25]. Psychological trauma and associated depression may mediate this association through neuroendocrine and behavioural pathways. |
| **High ambient temperature** | Elevated mean environmental temperature exposure, defined in GBD using high-resolution climate data linked to country- and subnational-level health outcomes. Chronic heat exposure is proposed to impair thermoregulatory insulin sensitivity, alter brown adipose tissue capacity, and change lifestyle behaviours (reduced physical activity, altered dietary patterns) that increase metabolic risk. | An ecological analysis across 50 countries found each 1°C rise in mean country temperature was associated with a 0.314 percentage-point higher T2DM prevalence, equating to a 6.4% increase in T2DM incidence per 1°C [26]. Physiological studies confirm heat-induced impairment of insulin-stimulated glucose disposal. |
| **Low ambient temperature** | Prolonged exposure to cold environmental temperatures, estimated using climate variables in the GBD framework. Cold exposure activates sympathetic nervous system activity and may alter dietary behaviour and physical activity patterns. Brown adipose tissue is acutely activated by cold but chronic sympathetic activation may have adverse metabolic sequelae in some populations. | Evidence linking cold ambient temperature to T2DM risk is limited and less consistent than for heat. Short-term cold acclimation studies demonstrate improvements in insulin sensitivity through brown adipose tissue activation, but population-level epidemiological evidence remains sparse and context-dependent [27]. |
| **Particulate matter pollution (PM2.5)** | Ambient concentration of fine particulate matter with aerodynamic diameter ≤2.5 μm (PM2.5), estimated using satellite data and land-use regression models in the GBD framework. PM2.5 exposure activates pulmonary and systemic oxidative stress, triggers pro-inflammatory pathways (NF-κB, IL-6, TNF-α), impairs endothelial insulin signalling, and disrupts pancreatic β-cell function. | A meta-analysis of 13 studies found a 10% higher T2DM risk per 10 μg/m³ increase in long-term PM2.5 exposure [28]. Associations are particularly strong in low- and middle-income countries where PM2.5 levels frequently exceed WHO guideline values. |
| **Ambient ozone pollution** | Ground-level ozone (O₃) concentration, a secondary air pollutant formed by photochemical reactions between NOx and volatile organic compounds, estimated in the GBD framework using atmospheric chemistry models. Ozone is a potent pulmonary and systemic oxidant that triggers inflammatory cascades, reduces insulin sensitivity, and has been associated with adverse glycaemic outcomes. | Epidemiological studies show positive associations between long-term ozone exposure and T2DM risk; a systematic review found approximately 10–14% higher T2DM incidence per 10 ppb increase in 8-hour ozone concentrations [29]. Evidence remains more limited and heterogeneous than for PM2.5. |
| **Nitrogen dioxide pollution (NO₂)** | Ambient nitrogen dioxide (NO₂) concentration, primarily from traffic-related combustion and industrial emissions, used as a proxy for traffic-related air pollution (TRAP) in the GBD framework. NO₂ exposure induces systemic inflammation, autonomic nervous system dysregulation, and endothelial dysfunction, all of which impair glucose metabolism and insulin sensitivity. | A large European cohort found a 14% higher T2DM incidence per 10 μg/m³ increase in residential NO₂ exposure [30]. Meta-analyses of >100,000 participants confirm consistent positive associations between long-term NO₂ exposure and T2DM risk. |
| **Iron deficiency** | Inadequate body iron stores defined by depleted serum ferritin, low transferrin saturation, or anaemia attributable to iron deficiency, as estimated in the GBD nutritional deficiency framework. Iron deficiency impairs mitochondrial electron transport chain function, reduces aerobic metabolic capacity of skeletal muscle, and may affect haemoglobin–A1c measurement accuracy. | The relationship between iron status and T2DM is complex: iron deficiency can impair β-cell function and cellular energy metabolism, while iron overload (haemochromatosis) is a well-established T2DM risk factor [31]. Population-level evidence for iron deficiency per se as a T2DM risk factor remains limited and context-dependent. |
| **Zinc deficiency** | Inadequate zinc status defined by low serum or plasma zinc concentrations (typically <10.7 μmol/L), reflecting insufficient dietary intake or absorption. Zinc is an essential cofactor for insulin biosynthesis and crystallisation within pancreatic β-cells and is required for downstream insulin receptor signalling and glucose transporter function. | Meta-analyses of observational studies and randomised trials report inverse associations between zinc status and T2DM risk; zinc supplementation improved fasting glucose, HbA1c, and insulin resistance biomarkers in deficient populations [32]. |
| **Alcohol use** | Habitual consumption of ethanol-containing beverages, modelled across a dose–response spectrum in the GBD framework from abstinence to heavy drinking. Moderate alcohol intake may acutely improve insulin sensitivity through adiponectin elevation and reduced hepatic glucose output; heavy drinking impairs β-cell function, promotes alcoholic liver disease, and increases overall caloric intake. | A meta-analysis of >35 cohort studies found a J-shaped dose–response: moderate alcohol intake (5–25 g/day) was associated with 20–30% lower T2DM risk, while heavy consumption (>50 g/day) was associated with modestly increased risk [33]. The protective association is attenuated after adjustment for related health behaviours. |
| **Second-hand smoke** | Involuntary exposure to tobacco smoke exhaled by smokers or released from burning tobacco products (also termed passive smoking or environmental tobacco smoke exposure), as estimated in the GBD tobacco risk factor module. Passive smoke contains >4,000 toxic compounds including reactive oxygen species, particulate matter, heavy metals, and nicotine metabolites that cause systemic inflammation and endothelial dysfunction. | A meta-analysis of >300,000 never-smokers from prospective cohort studies found a 26% higher T2DM risk with regular second-hand smoke exposure vs. no exposure [34]. Proposed mechanisms include oxidative stress–mediated peripheral insulin resistance and pro-inflammatory cytokine activation. |

**Abbreviations:** ADA, American Diabetes Association; AGEs, advanced glycation end-products; BMD, bone mineral density; BMI, body-mass index; CKD, chronic kidney disease; DHA, docosahexaenoic acid; DXA, dual-energy X-ray absorptiometry; EPA, eicosapentaenoic acid; eGFR, estimated glomerular filtration rate; FPG, fasting plasma glucose; GBD, Global Burden of Disease; GLUT4, glucose transporter type 4; HbA1c, glycated haemoglobin; HDL-C, high-density lipoprotein cholesterol; HPA, hypothalamic–pituitary–adrenal; IL-6, interleukin-6; IPV, intimate partner violence; iTFA, industrially produced trans fatty acid; LA, linoleic acid; LDL-C, low-density lipoprotein cholesterol; NF-κB, nuclear factor kappa-light-chain-enhancer of activated B cells; NO₂, nitrogen dioxide; O₃, ozone; PM2.5, particulate matter ≤2.5 μm; RAAS, renin–angiotensin–aldosterone system; TRAP, traffic-related air pollution; WHO, World Health Organization.

**Supplementary Table 2. Model selection criteria and diagnostic statistics for exponential smoothing state-space (ETS) models used in projecting incidence and mortality rates of type 2 diabetes mellitus among postmenopausal women globally and across SDI regions, 2022–2040.**

| **Region** | **ETS Model** | **AIC** | **BIC** | **Ljung–Box**  **p-value** | **RMSE** | **R²** |
| --- | --- | --- | --- | --- | --- | --- |
| **Panel A: Incidence rate of T2DM among postmenopausal women** | | | | | | |
| **Global** | **ETS (M,A,N)** | 193.92 | 201.25 | 0.99 | 0.006 | 0.986 |
| High SDI | **ETS (A,A,N)** | 177.71 | 185.04 | 0.68 | 2.43 | 0.978 |
| High-middle SDI | **ETS (M,A,N)** | 194.27 | 201.60 | 0.96 | 0.007 | 0.992 |
| Middle SDI | **ETS (M,A,N)** | 232.36 | 239.69 | 0.97 | 0.01 | 0.989 |
| Low-middle SDI | **ETS (M,A,N)** | 179.79 | 187.12 | 0.98 | 0.005 | 0.976 |
| Low SDI | **ETS (M,A,N)** | 155.33 | 162.65 | 0.99 | 0.004 | 0.991 |
| **Panel B: Mortality rate of T2DM among postmenopausal women** | | | | | | |
| **Global** | **ETS (M,Ad,N)** | 89.58 | 98.37 | 0.65 | 0.006 | 0.995 |
| High SDI | **ETS (M,Ad,N)** | 109.33 | 118.12 | 0.49 | 0.012 | 0.984 |
| High-middle SDI | **ETS (A,Ad,N)** | 114.63 | 123.62 | 0.76 | 0.88 | 0.946 |
| Middle SDI | **ETS (M,A,N)** | 120.70 | 128.03 | 0.58 | 0.009 | 0.965 |
| Low-middle SDI | **ETS (M,A,N)** | 139.96 | 144.29 | 0.64 | 0.010 | 0.979 |
| Low SDI | **ETS (A,Ad,N)** | 125.29 | 134.09 | 0.78 | 0.381 | 0.949 |

**Abbreviations:** AIC, Akaike Information Criterion; BIC, Bayesian Information Criterion; ETS, Error–Trend–Seasonality exponential smoothing state-space model; RMSE, root mean square error; SDI, Socio-demographic Index; T2DM, type 2 diabetes mellitus.

**ETS model notation:** ETS (Error, Trend, Seasonality) where Error = A (additive) or M (multiplicative); Trend = N (none), A (additive), or Ad (additive damped); Seasonality = N (none). Model specifications were selected automatically using the ets() function in the R forecast package, with the optimal model for each series chosen based on minimisation of corrected AIC (AICc) and BIC across all candidate ETS specifications.

**Ljung–Box test:** p-values from the Ljung–Box portmanteau test applied to model residuals (lag = 10). All p-values >0.05 indicate no significant autocorrelation in residuals, confirming adequate model fit.

**Supplementary Table 3. Incidence rate, mortality rate of T2DM among postmenopausal women across 204 countries and territories in 2021, and the estimated annual percent changes from 1990-2021.**

|  | Incidence | | Mortality | |
| --- | --- | --- | --- | --- |
| Location | Incidence rate (per 100,000 population), 2021 | EAPC (%) in incidence rates, 1990-2021 | Mortality rate (per 100,000 population), 2021 | EAPC (%) in mortality rates, 1990-2021 |
| Afghanistan | 1181·53 (883·35 to 1519·52) | 1·59 (1·47 to 1·71) | 272·81 (204·25 to 372·56) | 2·21 (2·10 to 2·32) |
| Albania | 461·43 (380·63 to 557·94) | 0·93 (0·83 to 1·04) | 25·20 (19·48 to 32·71) | 0·49 (0·18 to 0·80) |
| Algeria | 1068·71 (858·91 to 1296·89) | 1·90 (1·85 to 1·95) | 90·86 (72·35 to 111·59) | 2·32 (2·18 to 2·47) |
| American Samoa | 815·08 (612·76 to 1058·18) | -0·28 (-0·41 to -0·16) | 517·14 (418·31 to 622·40) | 1·24 (0·81 to 1·67) |
| Andorra | 394·09 (322·37 to 481·41) | 1·74 (1·62 to 1·86) | 45·72 (32·34 to 60·99) | 0·64 (0·33 to 0·96) |
| Angola | 601·78 (500·94 to 731·57) | 1·12 (1·06 to 1·19) | 140·69 (105·82 to 186·25) | -0·11 (-0·21 to -0·02) |
| Antigua and Barbuda | 1158·56 (937·06 to 1402·73) | 1·20 (1·09 to 1·31) | 289·23 (270·61 to 309·46) | -1·77 (-2·05 to -1·49) |
| Argentina | 699·86 (571·52 to 830·06) | 0·87 (0·79 to 0·96) | 77·89 (69·38 to 85·09) | -1·34 (-1·62 to -1·07) |
| Armenia | 543·02 (416·36 to 697·37) | 0·28 (0·15 to 0·42) | 77·46 (66·90 to 89·38) | -1·16 (-2·51 to 0·20) |
| Australia | 430·44 (357·56 to 506·70) | 1·47 (1·42 to 1·52) | 47·05 (37·23 to 53·19) | -0·98 (-1·36 to -0·60) |
| Austria | 317·59 (271·33 to 368·26) | 0·61 (0·53 to 0·69) | 61·94 (49·00 to 69·87) | -0·57 (-1·09 to -0·05) |
| Azerbaijan | 648·40 (484·59 to 837·03) | 3·24 (3·10 to 3·39) | 80·83 (57·11 to 106·02) | 1·03 (0·26 to 1·81) |
| Bahamas | 911·65 (732·03 to 1124·30) | 0·86 (0·83 to 0·90) | 153·91 (126·61 to 184·58) | -2·29 (-2·58 to -1·99) |
| Bahrain | 2384·00 (2019·23 to 2660·62) | 2·12 (1·88 to 2·37) | 405·29 (333·22 to 471·50) | -0·43 (-1·00 to 0·13) |
| Bangladesh | 575·47 (493·11 to 667·91) | 1·35 (1·23 to 1·48) | 164·22 (126·79 to 206·96) | 0·85 (0·60 to 1·10) |
| Barbados | 1064·89 (891·77 to 1229·53) | 0·15 (0·07 to 0·22) | 345·28 (282·42 to 413·37) | -1·5 (-1·82 to -1·18) |
| Belarus | 267·77 (214·65 to 326·63) | 1·01 (0·89 to 1·12) | 19·07 (15·86 to 22·84) | -2·41 (-3·37 to -1·44) |
| Belgium | 403·77 (328·41 to 491·53) | 0·55 (0·40 to 0·70) | 36·02 (27·22 to 41·68) | -2·57 (-2·80 to -2·33) |
| Belize | 893·93 (724·04 to 1091·29) | 0·59 (0·56 to 0·62) | 293·21 (260·32 to 330·42) | -0·35 (-0·95 to 0·24) |
| Benin | 795·46 (660·00 to 943·21) | 1·71 (1·67 to 1·75) | 153·77 (124·26 to 186·07) | 1·14 (0·95 to 1·33) |
| Bermuda | 598·23 (492·26 to 711·15) | 0·43 (0·36 to 0·49) | 91·34 (72·96 to 114·98) | -2·76 (-3·00 to -2·52) |
| Bhutan | 466·25 (386·84 to 563·93) | 1·83 (1·7 to 1·97) | 158·18 (112·47 to 204·55) | 1·67 (1·62 to 1·71) |
| Bolivia (Plurinational State of) | 973·81 (801·89 to 1128·48) | 1·63 (1·59 to 1·68) | 239·87 (189·01 to 312·86) | 0·00 (-0·07 to 0·07) |
| Bosnia and Herzegovina | 1060·70 (860·95 to 1244·93) | 1·95 (1·81 to 2·09) | 187·50 (147·97 to 230·58) | 3·64 (3·19 to 4·08) |
| Botswana | 1089·29 (961·97 to 1210·41) | 1·91 (1·85 to 1·97) | 284·71 (223·85 to 358·18) | 0·89 (0·37 to 1·42) |
| Brazil | 694·67 (573·37 to 837·99) | 0·41 (0·29 to 0·53) | 133·13 (116·98 to 142·83) | -0·73 (-0·89 to -0·56) |
| Brunei Darussalam | 1624·36 (1288·09 to 1969·46) | 0·10 (-0·05 to 0·25) | 185·78 (156·13 to 220·39) | -2·58 (-2·81 to -2·35) |
| Bulgaria | 601·59 (484·85 to 717·67) | 0·56 (0·31 to 0·8) | 89·84 (78·40 to 102·42) | -0·40 (-0·78 to -0·02) |
| Burkina Faso | 612·70 (508·86 to 721·79) | 1·34 (1·26 to 1·42) | 107·63 (87·20 to 133·75) | -0·38 (-0·50 to -0·26) |
| Burundi | 497·61 (430·46 to 572·97) | 0·11 (0·08 to 0·13) | 164·77 (112·22 to 224·65) | -1·35 (-1·58 to -1·12) |
| Cabo Verde | 986·99 (825·34 to 1161·74) | 2·25 (2·16 to 2·35) | 181·14 (145·00 to 221·95) | 3·73 (2·90 to 4·57) |
| Cambodia | 1059·02 (899·37 to 1226·59) | 2·99 (2·94 to 3·03) | 144·27 (102·98 to 204·65) | 0·21 (0·05 to 0·36) |
| Cameroon | 774·73 (671·11 to 893·00) | 2·02 (1·90 to 2·13) | 189·01 (134·08 to 259·89) | 1·04 (0·74 to 1·33) |
| Canada | 728·25 (590·37 to 878·12) | 2·11 (2·03 to 2·18) | 39·68 (33·09 to 44·23) | -2·63 (-3·30 to -1·96) |
| Central African Republic | 698·41 (556·88 to 869·92) | 1·02 (0·94 to 1·11) | 199·08 (143·33 to 261·44) | 0·04 (-0·13 to 0·21) |
| Chad | 720·72 (605·67 to 845·88) | 1·80 (1·65 to 1·95) | 170·08 (131·05 to 220·86) | 1·57 (1·25 to 1·90) |
| Chile | 938·73 (745·79 to 1156·70) | 2·51 (2·34 to 2·68) | 56·47 (49·00 to 61·79) | -1·00 (-1·43 to -0·56) |
| China | 381·68 (293·34 to 478·39) | 0·22 (-0·01 to 0·46) | 42·91 (34·05 to 52·94) | -0·48 (-0·81 to -0·15) |
| Colombia | 631·30 (514·34 to 757·55) | 0·27 (0·16 to 0·38) | 66·01 (54·86 to 76·72) | -2·66 (-3·04 to -2·29) |
| Comoros | 646·31 (555·78 to 753·98) | 0·60 (0·52 to 0·68) | 171·57 (117·58 to 241·61) | 0·04 (-0·02 to 0·09) |
| Congo | 713·03 (599·15 to 846·10) | 1·22 (1·16 to 1·28) | 228·15 (174·38 to 291·90) | -0·07 (-0·31 to 0·16) |
| Cook Islands | 865·79 (663·35 to 1110·30) | -0·72 (-0·80 to -0·64) | 533·03 (416·92 to 672·44) | -0·83 (-0·91 to -0·75) |
| Costa Rica | 709·35 (557·33 to 854·92) | 0·94 (0·86 to 1·02) | 101·25 (86·34 to 113·68) | -0·74 (-1·54 to 0·08) |
| Côte d'Ivoire | 830·40 (695·19 to 986·04) | 2·02 (1·93 to 2·10) | 159·25 (120·74 to 209·72) | 2·22 (1·85 to 2·59) |
| Croatia | 656·89 (532·89 to 772·60) | 0·35 (0·27 to 0·43) | 103·00 (88·02 to 114·95) | 0·78 (0·29 to 1·28) |
| Cuba | 621·01 (501·75 to 753·39) | 0·59 (0·50 to 0·67) | 59·17 (50·85 to 67·57) | -2·99 (-3·66 to -2·32) |
| Cyprus | 864·54 (761·33 to 957·81) | -1·83 (-1·88 to -1·77) | 142·89 (116·14 to 170·36) | -3·33 (-3·55 to -3·12) |
| Czechia | 691·43 (551·80 to 814·82) | 1·52 (1·39 to 1·66) | 115·49 (98·62 to 131·50) | 3·27 (2·29 to 4·27) |
| Democratic People's Republic of Korea | 451·18 (355·28 to 558·78) | 1·22 (1·12 to 1·31) | 77·61 (58·43 to 104·56) | 1·04 (0·77 to 1·31) |
| Democratic Republic of the Congo | 509·35 (414·91 to 605·06) | 1·10 (1·06 to 1·15) | 185·88 (125·96 to 248·19) | 0·59 (0·47 to 0·72) |
| Denmark | 343·28 (279·14 to 408·02) | 2·24 (2·17 to 2·32) | 57·41 (47·02 to 64·05) | 0·05 (-0·44 to 0·55) |
| Djibouti | 558·88 (486·43 to 632·62) | 1·45 (1·34 to 1·57) | 155·51 (105·31 to 223·57) | 0·66 (0·57 to 0·75) |
| Dominica | 1199·47 (972·62 to 1436·09) | 0·69 (0·6 to 0·78) | 422·86 (369·54 to 488·34) | -0·20 (-0·38 to -0·03) |
| Dominican Republic | 804·55 (657·69 to 976·47) | 1·39 (1·30 to 1·47) | 150·98 (122·28 to 187·52) | 1·09 (0·86 to 1·31) |
| Ecuador | 1002·06 (841·39 to 1203·25) | 1·80 (1·63 to 1·97) | 148·23 (121·79 to 180·81) | 0·43 (-0·03 to 0·91) |
| Egypt | 1094·04 (867·86 to 1362·22) | 3·86 (3·77 to 3·94) | 246·13 (201·21 to 299·05) | 2·75 (2·36 to 3·15) |
| El Salvador | 592·92 (480·92 to 703·21) | 1·60 (1·51 to 1·70) | 242·12 (194·78 to 305·35) | 2·39 (2·20 to 2·58) |
| Equa torial Guinea | 819·29 (695·10 to 953·30) | 2·46 (2·30 to 2·61) | 188·61 (120·55 to 278·35) | 0·31 (0·19 to 0·44) |
| Eritrea | 645·43 (562·74 to 737·07) | 1·33 (1·22 to 1·43) | 204·96 (148·28 to 264·59) | 0·74 (0·66 to 0·83) |
| Es tonia | 354·04 (291·15 to 439·45) | 1·27 (1·19 to 1·35) | 60·73 (51·11 to 69·03) | 2·60 (1·69 to 3·52) |
| Eswatini | 1555·57 (1395·48 to 1719·98) | 2·41 (2·25 to 2·56) | 520·91 (360·51 to 711·84) | 1·52 (0·91 to 2·14) |
| Ethiopia | 546·16 (471·80 to 635·60) | -0·24 (-0·29 to -0·19) | 138·83 (114·61 to 167·21) | -2·43 (-2·70 to -2·16) |
| Fiji | 1419·69 (1048·93 to 1753·37) | 0·27 (-0·05 to 0·58) | 1107·87 (871·36 to 1391·85) | 1·21 (0·96 to 1·47) |
| Finland | 425·89 (355·35 to 510·08) | 0·67 (0·59 to 0·75) | 23·22 (18·35 to 26·56) | -2·44 (-2·99 to -1·88) |
| France | 271·77 (221·04 to 327·07) | 0·16 (0·12 to 0·20) | 53·46 (42·51 to 60·16) | -0·44 (-0·89 to 0·02) |
| Gabon | 793·08 (686·62 to 925·52) | 1·46 (1·33 to 1·6) | 217·38 (149·93 to 285·51) | -0·01 (-0·38 to 0·36) |
| Gambia | 749·48 (629·40 to 891·87) | 1·93 (1·87 to 1·99) | 205·05 (152·89 to 272·34) | 2·04 (1·84 to 2·24) |
| Georgia | 413·28 (322·93 to 507·96) | 1·73 (1·63 to 1·83) | 87·57 (75·93 to 99·15) | 3·10 (2·28 to 3·93) |
| Germany | 423·14 (357·52 to 516·60) | 0·74 (0·57 to 0·92) | 60·92 (47·16 to 69·15) | -2·03 (-2·25 to -1·81) |
| Ghana | 735·61 (621·83 to 855·36) | 1·68 (1·46 to 1·90) | 161·27 (123·16 to 214·19) | 1·27 (1·10 to 1·43) |
| Greece | 328·66 (255·11 to 398·01) | -0·50 (-0·61 to -0·38) | 44·09 (36·65 to 49·48) | -0·30 (-0·80 to 0·21) |
| Greenland | 465·90 (394·55 to 552·00) | 2·22 (1·92 to 2·53) | 25·47 (20·71 to 31·63) | -3·32 (-3·54 to -3·10) |
| Grenada | 1107·44 (881·37 to 1332·60) | 1·14 (1·02 to 1·25) | 407·25 (361·37 to 448·28) | -0·90 (-1·19 to -0·61) |
| Guam | 536·68 (433·25 to 645·67) | -0·47 (-0·70 to -0·25) | 86·34 (69·90 to 102·81) | -2·83 (-3·07 to -2·58) |
| Guatemala | 820·92 (676·42 to 973·09) | 1·74 (1·39 to 2·10) | 330·53 (288·70 to 375·83) | 3·29 (2·71 to 3·88) |
| Guinea | 693·15 (606·77 to 801·59) | 1·26 (1·07 to 1·45) | 206·50 (158·36 to 262·02) | 1·54 (1·23 to 1·85) |
| Guinea-Bissau | 899·55 (762·55 to 1064·96) | 2·02 (1·95 to 2·10) | 213·91 (161·15 to 278·29) | 1·43 (1·18 to 1·68) |
| Guyana | 1059·24 (789·74 to 1359·30) | 0·05 (-0·06 to 0·16) | 386·90 (307·46 to 482·42) | -0·55 (-1·08 to -0·03) |
| Haiti | 1130·71 (853·27 to 1436·53) | 0·37 (0·29 to 0·45) | 466·81 (348·47 to 626·48) | -0·17 (-0·28 to -0·07) |
| Honduras | 710·24 (567·07 to 875·97) | 1·62 (1·48 to 1·76) | 160·85 (116·06 to 219·32) | 2·64 (2·29 to 2·99) |
| Hungary | 583·98 (483·90 to 688·10) | 0·57 (0·32 to 0·83) | 76·89 (66·12 to 85·51) | 0·36 (0·00 to 0·72) |
| Iceland | 336·29 (268·19 to 398·56) | 1·67 (1·62 to 1·72) | 20·07 (15·60 to 22·96) | -1·54 (-1·77 to -1·32) |
| India | 434·98 (358·55 to 519·45) | 0·94 (0·85 to 1·03) | 139·81 (121·07 to 160·84) | 1·67 (1·44 to 1·90) |
| Indonesia | 771·93 (640·63 to 905·01) | 3·19 (2·80 to 3·58) | 125·19 (97·87 to 158·77) | 0·80 (0·58 to 1·01) |
| Iran (Islamic Republic of) | 870·27 (703·15 to 1068·03) | 2·74 (2·59 to 2·88) | 107·70 (93·61 to 120·05) | 2·41 (2·24 to 2·59) |
| Iraq | 1109·78 (840·13 to 1387·90) | 1·78 (1·70 to 1·86) | 192·35 (149·20 to 238·60) | -0·06 (-0·20 to 0·08) |
| Ireland | 355·86 (291·34 to 426·28) | 1·21 (1·14 to 1·27) | 24·92 (19·69 to 28·86) | -2·60 (-2·82 to -2·38) |
| Israel | 697·85 (613·80 to 798·53) | 0·78 (0·50 to 1·07) | 115·60 (92·32 to 130·52) | -0·73 (-1·64 to 0·19) |
| Italy | 452·50 (364·00 to 551·56) | -0·32 (-0·42 to -0·21) | 79·64 (60·93 to 89·94) | -1·25 (-1·38 to -1·11) |
| Jamaica | 1150·49 (1008·18 to 1301·60) | 0·84 (0·78 to 0·9) | 452·85 (366·80 to 557·58) | -0·34 (-0·61 to -0·07) |
| Japan | 488·43 (408·48 to 580·68) | 0·23 (0·02 to 0·45) | 16·66 (11·79 to 19·53) | -2·16 (-2·95 to -1·36) |
| Jordan | 1520·89 (1198·23 to 1817·38) | 0·15 (0·01 to 0·28) | 164·54 (133·59 to 200·20) | -2·57 (-3·24 to -1·91) |
| Kazakhstan | 654·37 (498·41 to 782·83) | 2·98 (2·89 to 3·07) | 43·85 (35·80 to 52·21) | -0·82 (-1·3 to -0·34) |
| Kenya | 408·70 (355·58 to 466·57) | 0·32 (0·21 to 0·44) | 124·77 (93·63 to 159·13) | 1·06 (0·86 to 1·26) |
| Kiribati | 908·38 (718·60 to 1150·68) | -0·03 (-0·11 to 0·06) | 795·13 (625·87 to 993·91) | 0·86 (0·69 to 1·03) |
| Kuwait | 1601·36 (1204·20 to 2057·43) | 2·21 (2·06 to 2·35) | 83·88 (68·14 to 97·52) | -1·27 (-1·85 to -0·68) |
| Kyrgyzstan | 368·23 (285·31 to 479·76) | 2·38 (2·28 to 2·47) | 37·13 (31·18 to 43·84) | -0·19 (-0·68 to 0·30) |
| Lao People's Democratic Republic | 1329·34 (1094·97 to 1553·30) | 2·56 (2·54 to 2·59) | 188·86 (144·36 to 237·58) | -0·16 (-0·31 to -0·01) |
| Latvia | 369·32 (287·82 to 464·47) | 1·85 (1·75 to 1·94) | 81·17 (70·77 to 90·74) | 3·23 (2·71 to 3·75) |
| Lebanon | 846·43 (674·48 to 1020·56) | 1·07 (1·02 to 1·12) | 125·29 (99·87 to 153·21) | -0·13 (-0·23 to -0·03) |
| Lesotho | 1216·11 (1094·81 to 1349·76) | 3·18 (3·01 to 3·35) | 473·78 (336·10 to 632·44) | 3·72 (3·08 to 4·37) |
| Liberia | 728·65 (614·49 to 858·62) | 1·61 (1·56 to 1·66) | 195·18 (144·84 to 257·92) | 1·30 (0·99 to 1·60) |
| Libya | 963·26 (715·47 to 1230·62) | 2·58 (2·49 to 2·66) | 119·61 (89·10 to 161·87) | 2·77 (2·39 to 3·16) |
| Lithuania | 296·29 (239·41 to 374·36) | 1·11 (1·00 to 1·21) | 49·15 (42·84 to 55·35) | 2·49 (1·57 to 3·42) |
| Luxembourg | 403·19 (332·99 to 483·35) | 0·86 (0·75 to 0·98) | 37·20 (30·53 to 42·76) | -1·67 (-1·92 to -1·42) |
| Madagascar | 489·80 (423·28 to 563·82) | 0·77 (0·72 to 0·82) | 131·77 (96·19 to 176·58) | -0·35 (-0·42 to -0·28) |
| Malawi | 457·15 (403·16 to 512·42) | 0·24 (0·19 to 0·29) | 159·30 (123·36 to 203·54) | -0·30 (-0·58 to -0·02) |
| Malaysia | 1382·97 (1183·07 to 1656·79) | 1·87 (1·56 to 2·19) | 94·16 (80·28 to 110·61) | -1·91 (-2·20 to -1·62) |
| Maldives | 1063·75 (890·73 to 1256·03) | 1·45 (1·35 to 1·55) | 83·67 (66·83 to 102·96) | -2·55 (-2·74 to -2·35) |
| Mali | 1195·74 (962·23 to 1429·86) | 2·00 (1·96 to 2·04) | 202·48 (154·93 to 258·97) | 1·26 (1·14 to 1·38) |
| Malta | 570·50 (466·72 to 671·41) | 0·76 (0·45 to 1·07) | 100·16 (80·70 to 115·13) | -1·72 (-2·14 to -1·30) |
| Marshall Islands | 818·48 (606·05 to 1085·03) | -0·11 (-0·19 to -0·04) | 894·97 (563·50 to 1235·91) | 1·12 (0·78 to 1·46) |
| Mauritania | 591·19 (522·27 to 669·59) | 1·02 (0·87 to 1·17) | 171·96 (123·83 to 233·83) | 0·39 (0·20 to 0·58) |
| Mauritius | 1950·71 (1702·04 to 2211·02) | 2·72 (2·57 to 2·86) | 454·35 (414·37 to 483·67) | 3·48 (2·52 to 4·46) |
| Mexico | 862·63 (705·01 to 1024·94) | -0·82 (-1·01 to -0·63) | 319·24 (275·27 to 362·68) | -0·75 (-1·00 to -0·49) |
| Micronesia (Federated States of) | 1099·46 (876·64 to 1355·66) | 0·79 (0·73 to 0·85) | 515·43 (395·42 to 668·86) | 0·58 (0·21 to 0·94) |
| Monaco | 285·91 (232·85 to 348·19) | 1·84 (1·79 to 1·89) | 22·08 (15·43 to 28·77) | -0·34 (-0·43 to -0·25) |
| Mongolia | 291·84 (231·90 to 358·53) | 2·25 (2·10 to 2·40) | 32·01 (26·11 to 38·26) | 0·99 (0·80 to 1·19) |
| Montenegro | 827·77 (678·94 to 973·50) | 0·87 (0·80 to 0·95) | 92·74 (78·01 to 110·53) | 1·66 (1·41 to 1·92) |
| Morocco | 1065·96 (804·49 to 1366·61) | 2·09 (2·03 to 2·16) | 127·57 (91·12 to 161·07) | 2·69 (2·48 to 2·91) |
| Mozambique | 520·10 (451·08 to 601·19) | 1·06 (0·99 to 1·14) | 150·40 (106·54 to 197·46) | 0·85 (0·69 to 1·00) |
| Myanmar | 1476·37 (1250·11 to 1732·72) | 2·23 (2·16 to 2·3) | 246·87 (197·80 to 311·54) | -0·45 (-0·63 to -0·28) |
| Namibia | 855·91 (732·71 to 978·35) | 0·87 (0·80 to 0·94) | 333·48 (238·36 to 437·84) | -0·01 (-0·35 to 0·34) |
| Nauru | 1061·86 (813·98 to 1365·18) | 0·36 (0·26 to 0·46) | 644·28 (489·68 to 849·33) | 0·82 (0·61 to 1·02) |
| Nepal | 605·49 (475·72 to 732·92) | 2·11 (2·00 to 2·21) | 133·90 (99·38 to 177·57) | 1·40 (1·20 to 1·60) |
| Netherlands | 348·71 (292·20 to 416·24) | -0·65 (-0·74 to -0·56) | 50·99 (41·19 to 57·69) | -2·95 (-3·29 to -2·60) |
| New Zealand | 487·18 (432·33 to 546·83) | 2·11 (2·00 to 2·22) | 35·94 (29·68 to 40·38) | -2·07 (-2·59 to -1·54) |
| Nicaragua | 860·82 (678·51 to 1047·15) | 1·16 (1·11 to 1·20) | 155·54 (127·06 to 189·07) | 0·63 (0·35 to 0·91) |
| Niger | 746·38 (614·47 to 926·17) | 1·92 (1·84 to 1·99) | 110·73 (76·25 to 156·61) | 1·08 (1·00 to 1·17) |
| Nigeria | 629·64 (536·51 to 727·06) | 1·16 (1·09 to 1·23) | 133·47 (99·85 to 179·20) | -0·60 (-0·73 to -0·47) |
| Niue | 822·69 (622·27 to 1072·41) | -0·21 (-0·34 to -0·09) | 663·48 (476·09 to 836·72) | 1·13 (1·01 to 1·25) |
| North Macedonia | 1147·78 (929·68 to 1366·83) | 1·50 (1·35 to 1·65) | 170·18 (136·21 to 208·65) | 1·56 (1·17 to 1·96) |
| Northern Mariana Islands | 733·78 (588·55 to 889·33) | 0·91 (0·82 to 1·00) | 219·72 (185·94 to 249·10) | -1·06 (-1·34 to -0·77) |
| Norway | 348·79 (285·01 to 420·41) | 0·09 (-0·14 to 0·32) | 33·81 (27·10 to 37·52) | -0·99 (-1·64 to -0·35) |
| Oman | 976·91 (790·24 to 1190·06) | 2·20 (1·97 to 2·44) | 171·04 (136·71 to 209·97) | -0·45 (-0·63 to -0·27) |
| Pakistan | 852·99 (709·81 to 1005·87) | 3·51 (3·30 to 3·71) | 229·46 (169·90 to 300·81) | 1·31 (1·01 to 1·61) |
| Palau | 1051·59 (808·33 to 1355·79) | 0·13 (0·01 to 0·25) | 459·47 (352·28 to 574·52) | 0·58 (0·39 to 0·77) |
| Palestine | 1353·64 (1097·27 to 1598·41) | 1·76 (1·69 to 1·83) | 234·90 (196·45 to 277·29) | 0·28 (0·01 to 0·56) |
| Panama | 721·94 (579·80 to 900·75) | 0·93 (0·85 to 1·00) | 157·13 (123·58 to 187·61) | 0·47 (0·18 to 0·77) |
| Papua New Guinea | 765·21 (613·95 to 935·63) | 1·49 (1·41 to 1·57) | 279·98 (217·20 to 351·28) | 0·35 (0·23 to 0·47) |
| Paraguay | 907·84 (726·00 to 1079·55) | 1·87 (1·73 to 2·00) | 299·31 (228·88 to 378·53) | 2·49 (2·06 to 2·93) |
| Peru | 604·56 (524·77 to 697·89) | 1·88 (1·72 to 2·05) | 92·88 (70·92 to 122·24) | 0·59 (0·30 to 0·89) |
| Philippines | 1003·73 (863·35 to 1162·80) | 0·57 (0·51 to 0·63) | 175·36 (142·60 to 213·29) | 1·28 (1·20 to 1·35) |
| Poland | 654·12 (548·75 to 785·10) | 1·10 (0·93 to 1·28) | 75·08 (64·33 to 83·19) | 0·23 (-0·17 to 0·62) |
| Portugal | 684·51 (564·24 to 838·20) | 1·21 (1·13 to 1·28) | 103·00 (80·84 to 117·31) | -1·05 (-1·34 to -0·75) |
| Puer to Rico | 867·36 (681·20 to 1071·63) | -0·33 (-0·41 to -0·25) | 227·15 (185·76 to 267·99) | -1·23 (-1·44 to -1·01) |
| Qatar | 2725·04 (2097·64 to 3129·25) | 1·23 (0·77 to 1·69) | 238·24 (191·96 to 298·16) | -0·26 (-1·21 to 0·69) |
| Republic of Korea | 962·70 (807·76 to 1108·92) | 2·22 (2·13 to 2·31) | 56·32 (42·69 to 67·39) | -1·82 (-2·52 to -1·13) |
| Republic of Moldova | 446·85 (346·51 to 551·32) | 1·26 (1·07 to 1·44) | 43·41 (37·91 to 49·11) | -0·36 (-1·15 to 0·43) |
| Romania | 367·64 (292·36 to 450·27) | 0·72 (0·62 to 0·82) | 40·29 (35·66 to 45·72) | 0·40 (0·05 to 0·75) |
| Russian Federation | 448·99 (373·25 to 539·23) | 1·85 (1·78 to 1·93) | 103·01 (92·52 to 112·20) | 3·86 (2·32 to 5·42) |
| Rwanda | 470·39 (415·18 to 535·65) | 0·15 (0·10 to 0·20) | 138·20 (81·90 to 212·53) | -2·18 (-2·55 to -1·81) |
| Saint Kitts and Nevis | 1060·35 (850·46 to 1298·47) | 1·05 (0·80 to 1·30) | 202·07 (171·01 to 234·79) | -2·41 (-2·95 to -1·86) |
| Saint Lucia | 1115·21 (917·48 to 1327·99) | -0·64 (-0·72 to -0·55) | 321·35 (269·22 to 376·89) | -3·01 (-3·42 to -2·61) |
| Saint Vincent and the Grenadines | 1154·01 (918·75 to 1427·89) | -0·35 (-0·42 to -0·27) | 363·56 (322·13 to 410·20) | -2·15 (-2·55 to -1·76) |
| Samoa | 813·36 (630·02 to 1039·00) | 0·19 (0·07 to 0·31) | 485·11 (370·18 to 627·07) | 1·53 (1·38 to 1·69) |
| San Marino | 366·86 (301·84 to 449·37) | 1·41 (1·32 to 1·51) | 30·07 (19·14 to 41·80) | -1·41 (-1·75 to -1·08) |
| Sao tome and Principe | 723·67 (586·72 to 871·86) | 1·75 (1·71 to 1·79) | 61·29 (47·68 to 75·42) | 0·55 (0·21 to 0·89) |
| Saudi Arabia | 1067·51 (842·63 to 1321·29) | 1·87 (1·83 to 1·9) | 101·73 (80·58 to 127·97) | -0·64 (-0·77 to -0·51) |
| Senegal | 1144·51 (954·50 to 1323·14) | 2·28 (2·17 to 2·4) | 220·84 (163·14 to 288·89) | 1·75 (1·51 to 1·99) |
| Serbia | 844·23 (698·77 to 999·01) | 0·58 (0·47 to 0·68) | 132·20 (110·14 to 158·08) | 0·89 (0·63 to 1·15) |
| Seychelles | 1801·09 (1546·28 to 2071·68) | 3·15 (3·06 to 3·25) | 129·25 (109·57 to 150·01) | 1·60 (1·25 to 1·94) |
| Sierra Leone | 744·43 (619·09 to 895·40) | 1·95 (1·88 to 2·01) | 161·37 (123·99 to 208·83) | 1·85 (1·48 to 2·21) |
| Singapore | 692·99 (540·95 to 833·68) | 0·89 (0·76 to 1·03) | 10·19 (8·30 to 11·54) | -7·42 (-8·53 to -6·29) |
| Slovakia | 516·71 (422·16 to 614·64) | 0·73 (0·64 to 0·83) | 47·73 (37·52 to 58·18) | -1·09 (-1·20 to -0·97) |
| Slovenia | 502·25 (407·39 to 591·63) | -0·70 (-0·84 to -0·55) | 50·18 (41·03 to 58·20) | -2·56 (-3·27 to -1·85) |
| Solomon Islands | 971·97 (841·10 to 1112·92) | 2·20 (2·00 to 2·40) | 446·74 (340·81 to 572·12) | 1·18 (1·04 to 1·32) |
| Somalia | 577·13 (496·98 to 649·00) | 1·14 (1·05 to 1·23) | 199·41 (144·15 to 260·55) | 0·41 (0·37 to 0·45) |
| South Africa | 1273·38 (1120·06 to 1443·51) | 2·34 (2·23 to 2·45) | 385·28 (353·38 to 415·05) | 2·22 (1·67 to 2·78) |
| South Sudan | 485·05 (424·70 to 555·24) | 0·44 (0·33 to 0·54) | 183·80 (130·21 to 239·08) | -0·06 (-0·18 to 0·06) |
| Spain | 674·20 (572·11 to 770·38) | 0·60 (0·47 to 0·73) | 60·08 (45·50 to 69·67) | -2·16 (-2·32 to -2·00) |
| Sri Lanka | 1953·55 (1676·36 to 2256·52) | 1·90 (1·80 to 1·99) | 235·31 (163·00 to 311·93) | 2·00 (1·76 to 2·24) |
| Sudan | 819·88 (643·96 to 998·77) | 2·0 (2·00 to 2·11) | 95·59 (72·45 to 129·74) | 1·34 (1·23 to 1·46) |
| Suriname | 900·97 (698·13 to 1130·12) | 0·87 (0·82 to 0·92) | 201·09 (161·00 to 247·75) | 0·32 (0·12 to 0·52) |
| Sweden | 395·00 (315·77 to 482·96) | 0·94 (0·89 to 1·00) | 53·16 (41·90 to 61·82) | -0·23 (-0·52 to 0·05) |
| Switzerland | 577·44 (477·22 to 674·79) | 0·80 (0·70 to 0·90) | 36·83 (27·85 to 42·71) | -3·54 (-3·81 to -3·27) |
| Syrian Arab Republic | 1044·30 (830·15 to 1278·35) | 1·92 (1·84 to 2·01) | 84·75 (63·85 to 110·52) | -0·84 (-1·11 to -0·57) |
| Taiwan (Province of China) | 748·13 (605·03 to 906·12) | 0·41 (0·27 to 0·54) | 121·54 (100·45 to 136·31) | -2·81 (-3·41 to -2·21) |
| Tajikistan | 443·56 (344·24 to 548·39) | 3·31 (3·16 to 3·46) | 66·05 (49·19 to 83·56) | 0·34 (-0·19 to 0·88) |
| Thailand | 991·27 (816·13 to 1184·74) | 1·90 (1·86 to 1·94) | 122·11 (92·67 to 156·27) | -0·81 (-1·12 to -0·51) |
| Timor-Leste | 1212·78 (1016·20 to 1427·38) | 3·63 (3·55 to 3·7) | 131·26 (101·93 to 175·07) | 1·02 (0·79 to 1·24) |
| togo | 608·43 (522·72 to 702·90) | 1·83 (1·76 to 1·90) | 131·75 (97·34 to 175·51) | 1·16 (1·07 to 1·26) |
| tokelau | 768·41 (608·82 to 956·00) | -0·33 (-0·47 to -0·18) | 506·72 (380·06 to 667·50) | 0·55 (0·38 to 0·72) |
| tonga | 815·84 (637·44 to 1030·52) | -0·43 (-0·54 to -0·31) | 637·80 (483·25 to 812·24) | 1·16 (1·07 to 1·26) |
| Trinidad and tobago | 1171·05 (898·09 to 1443·97) | 0·45 (0·31 to 0·58) | 451·26 (354·26 to 556·87) | -1·60 (-1·79 to -1·41) |
| Tunisia | 965·65 (768·19 to 1187·44) | 2·34 (2·22 to 2·47) | 77·11 (56·83 to 101·91) | 1·91 (1·73 to 2·08) |
| Türkiye | 786·70 (649·09 to 948·58) | 1·02 (0·96 to 1·08) | 136·75 (108·07 to 168·12) | -0·09 (-0·53 to 0·35) |
| Turkmenistan | 321·71 (232·93 to 398·86) | 2·25 (2·16 to 2·34) | 98·97 (76·60 to 128·13) | 1·66 (1·26 to 2·07) |
| Tuvalu | 1000·51 (815·57 to 1184·40) | 0·83 (0·68 to 0·97) | 524·03 (406·67 to 681·45) | 0·64 (0·52 to 0·77) |
| Uganda | 472·25 (416·76 to 530·91) | 0·53 (0·45 to 0·62) | 162·10 (108·01 to 225·83) | 0·05 (-0·23 to 0·33) |
| Ukraine | 301·73 (239·16 to 374·98) | 1·31 (1·16 to 1·45) | 15·23 (10·48 to 21·09) | -1·98 (-2·53 to -1·44) |
| United Arab Emirates | 1720·26 (1384·68 to 2081·87) | 0·57 (0·28 to 0·85) | 141·32 (112·06 to 176·81) | -0·34 (-1·12 to 0·45) |
| United Kingdom | 406·31 (334·83 to 484·36) | 0·76 (0·67 to 0·85) | 27·35 (22·59 to 29·73) | -2·00 (-2·26 to -1·75) |
| United Republic of Tanzania | 512·53 (456·11 to 581·48) | 1·27 (1·15 to 1·38) | 154·77 (122·39 to 200·83) | 0·48 (0·39 to 0·58) |
| United States of America | 918·27 (806·58 to 1036·77) | 4·02 (3·71 to 4·34) | 53·58 (45·96 to 57·95) | -2·29 (-2·85 to -1·73) |
| United States Virgin Islands | 1017·18 (807·62 to 1238·69) | 0·06 (-0·11 to 0·22) | 136·62 (106·78 to 171·12) | -1·90 (-2·18 to -1·62) |
| Uruguay | 570·38 (461·88 to 686·29) | 1·88 (1·73 to 2·02) | 94·99 (80·82 to 104·79) | -0·05 (-0·13 to 0·03) |
| Uzbekistan | 668·93 (482·56 to 805·87) | 4·53 (4·34 to 4·71) | 120·08 (100·55 to 140·57) | 3·16 (2·62 to 3·71) |
| Vanuatu | 854·11 (694·53 to 1049·17) | 1·49 (1·43 to 1·55) | 407·03 (320·43 to 494·39) | 0·71 (0·62 to 0·8) |
| Venezuela (Bolivarian Republic of) | 717·91 (579·49 to 882·11) | 0·94 (0·86 to 1·02) | 201·41 (160·85 to 250·77) | 0·12 (-0·26 to 0·51) |
| Viet Nam | 1089·47 (963·54 to 1247·55) | 1·85 (1·76 to 1·95) | 182·55 (144·99 to 226·39) | 0·40 (0·24 to 0·55) |
| Yemen | 677·25 (550·55 to 833·88) | 3·15 (2·74 to 3·56) | 75·68 (52·84 to 114·85) | 0·82 (0·73 to 0·91) |
| Zambia | 653·39 (552·97 to 762·37) | 0·63 (0·54 to 0·72) | 150·61 (111·12 to 201·00) | -0·51 (-0·67 to -0·35) |
| Zimbabwe | 1024·85 (902·84 to 1169·52) | 2·18 (2·04 to 2·33) | 369·11 (276·35 to 482·56) | 2·76 (2·00 to 3·53) |

**Supplementary Table 4. Comparison of incidence and mortality rates of diabetes in postmenopausal women globally in 2021, 2022 and 2023**

| **Location** | **Incidence rate 2021** | **Incidence rate 2022** | **Incidence rate 2023** | **Death rate 2021** | **Death rate 2022** | **Death rate 2023** |
| --- | --- | --- | --- | --- | --- | --- |
| Afghanistan | 1181.53 (883.35, 1519.52) | 1200.20 (897.31, 1543.53) | 1219.16 (911.48, 1567.92) | 272.81 (204.25, 372.56) | 279.01 (208.88, 381.02) | 285.34 (213.62, 389.67) |
| Albania | 461.43 (380.63, 557.94) | 465.86 (384.29, 563.30) | 470.33 (387.98, 568.71) | 25.20 (19.48, 32.71) | 25.32 (19.57, 32.86) | 25.44 (19.66, 33.02) |
| Algeria | 1068.71 (858.91, 1296.89) | 1089.12 (875.31, 1321.66) | 1109.92 (892.03, 1346.90) | 90.86 (72.35, 111.59) | 93.02 (74.07, 114.25) | 95.24 (75.83, 116.97) |
| American Samoa | 815.08 (612.76, 1058.18) | 812.63 (610.92, 1055.01) | 810.19 (609.09, 1051.84) | 517.14 (418.31, 622.40) | 524.02 (423.87, 630.68) | 530.99 (429.51, 639.06) |
| Andorra | 394.09 (322.37, 481.41) | 400.79 (327.85, 489.59) | 407.60 (333.42, 497.92) | 45.72 (32.34, 60.99) | 46.08 (32.60, 61.48) | 46.45 (32.86, 61.97) |
| Angola | 601.78 (500.94, 731.57) | 608.58 (506.61, 739.83) | 615.45 (512.33, 748.19) | 140.69 (105.82, 186.25) | 140.51 (105.68, 186.01) | 140.33 (105.55, 185.76) |
| Antigua and Barbuda | 1158.56 (937.06, 1402.73) | 1172.69 (948.50, 1419.85) | 1187.00 (960.07, 1437.17) | 289.23 (270.61, 309.46) | 284.20 (265.90, 304.07) | 279.26 (261.28, 298.78) |
| Argentina | 699.86 (571.52, 830.06) | 705.60 (576.21, 836.86) | 711.39 (580.93, 843.72) | 77.89 (69.38, 85.09) | 76.89 (68.49, 84.00) | 75.91 (67.61, 82.93) |
| Armenia | 543.02 (416.36, 697.37) | 544.70 (417.65, 699.54) | 546.39 (418.94, 701.70) | 77.46 (66.90, 89.38) | 76.85 (66.37, 88.68) | 76.24 (65.85, 87.98) |
| Australia | 430.44 (357.56, 506.70) | 436.76 (362.81, 514.15) | 443.18 (368.15, 521.71) | 47.05 (37.23, 53.19) | 46.61 (36.89, 52.69) | 46.17 (36.55, 52.20) |
| Austria | 317.59 (271.33, 368.26) | 319.56 (273.01, 370.54) | 321.54 (274.71, 372.84) | 61.94 (49.00, 69.87) | 61.64 (48.76, 69.54) | 61.35 (48.53, 69.20) |
| Azerbaijan | 648.40 (484.59, 837.03) | 669.48 (500.34, 864.23) | 691.24 (516.60, 892.32) | 80.83 (57.11, 106.02) | 81.82 (57.81, 107.31) | 82.82 (58.51, 108.62) |
| Bahamas | 911.65 (732.03, 1124.30) | 919.40 (738.26, 1133.86) | 927.21 (744.53, 1143.50) | 153.91 (126.61, 184.58) | 150.43 (123.75, 180.41) | 147.03 (120.96, 176.33) |
| Bahrain | 2384.00 (2019.23, 2660.62) | 2437.17 (2064.26, 2719.95) | 2491.52 (2110.30, 2780.60) | 405.29 (333.22, 471.50) | 403.79 (331.99, 469.76) | 402.29 (330.76, 468.02) |
| Bangladesh | 575.47 (493.11, 667.91) | 583.52 (500.01, 677.26) | 591.69 (507.01, 686.74) | 164.22 (126.79, 206.96) | 165.59 (127.84, 208.67) | 166.96 (128.90, 210.41) |
| Barbados | 1064.89 (891.77, 1229.53) | 1066.81 (893.37, 1231.75) | 1068.73 (894.98, 1233.96) | 345.28 (282.42, 413.37) | 340.24 (278.30, 407.33) | 335.27 (274.24, 401.38) |
| Belarus | 267.77 (214.65, 326.63) | 270.45 (216.80, 329.90) | 273.15 (218.97, 333.20) | 19.07 (15.86, 22.84) | 18.56 (15.43, 22.23) | 18.06 (15.02, 21.63) |
| Belgium | 403.77 (328.41, 491.53) | 405.71 (329.99, 493.89) | 407.66 (331.57, 496.26) | 36.02 (27.22, 41.68) | 35.12 (26.53, 40.63) | 34.23 (25.87, 39.61) |
| Belize | 893.93 (724.04, 1091.29) | 899.20 (728.31, 1097.72) | 904.51 (732.61, 1104.20) | 293.21 (260.32, 330.42) | 292.45 (259.64, 329.56) | 291.69 (258.97, 328.71) |
| Benin | 795.46 (660.00, 943.21) | 809.14 (671.35, 959.43) | 823.06 (682.89, 975.93) | 153.77 (124.26, 186.07) | 155.63 (125.76, 188.32) | 157.51 (127.28, 190.60) |
| Bermuda | 598.23 (492.26, 711.15) | 600.62 (494.23, 714.00) | 603.03 (496.21, 716.85) | 91.34 (72.96, 114.98) | 88.72 (70.87, 111.68) | 86.17 (68.84, 108.47) |
| Bhutan | 466.25 (386.84, 563.93) | 474.83 (393.96, 574.30) | 483.56 (401.21, 584.87) | 158.18 (112.47, 204.55) | 160.84 (114.36, 207.99) | 163.54 (116.28, 211.48) |
| Bolivia (Plurinational State of) | 973.81 (801.89, 1128.48) | 989.78 (815.04, 1146.99) | 1006.02 (828.41, 1165.80) | 239.87 (189.01, 312.86) | 239.95 (189.07, 312.96) | 240.02 (189.12, 313.05) |
| Bosnia and Herzegovina | 1060.70 (860.95, 1244.93) | 1082.02 (878.26, 1269.96) | 1103.77 (895.91, 1295.48) | 187.50 (147.97, 230.58) | 194.63 (153.60, 239.34) | 202.02 (159.43, 248.44) |
| Botswana | 1089.29 (961.97, 1210.41) | 1110.42 (980.63, 1233.89) | 1131.96 (999.66, 1257.83) | 284.71 (223.85, 358.18) | 287.58 (226.11, 361.80) | 290.49 (228.39, 365.46) |
| Brazil | 694.67 (573.37, 837.99) | 697.31 (575.55, 841.17) | 699.96 (577.73, 844.37) | 133.13 (116.98, 142.83) | 132.22 (116.17, 141.84) | 131.30 (115.37, 140.87) |
| Brunei Darussalam | 1624.36 (1288.09, 1969.46) | 1625.17 (1288.73, 1970.44) | 1625.98 (1289.38, 1971.43) | 185.78 (156.13, 220.39) | 181.06 (152.16, 214.79) | 176.46 (148.30, 209.33) |
| Bulgaria | 601.59 (484.85, 717.67) | 605.26 (487.81, 722.05) | 608.95 (490.79, 726.45) | 89.84 (78.40, 102.42) | 89.48 (78.08, 102.01) | 89.12 (77.77, 101.60) |
| Burkina Faso | 612.70 (508.86, 721.79) | 620.67 (515.48, 731.17) | 628.74 (522.18, 740.68) | 107.63 (87.20, 133.75) | 107.23 (86.88, 133.26) | 106.84 (86.55, 132.77) |
| Burundi | 497.61 (430.46, 572.97) | 498.11 (430.89, 573.55) | 498.61 (431.32, 574.12) | 164.77 (112.22, 224.65) | 162.50 (110.68, 221.55) | 160.25 (109.15, 218.49) |
| Cabo Verde | 986.99 (825.34, 1161.74) | 1009.00 (843.74, 1187.65) | 1031.50 (862.56, 1214.14) | 181.14 (145.00, 221.95) | 188.33 (150.76, 230.76) | 195.80 (156.74, 239.92) |
| Cambodia | 1059.02 (899.37, 1226.59) | 1090.80 (926.35, 1263.38) | 1123.52 (954.14, 1301.29) | 144.27 (102.98, 204.65) | 144.50 (103.15, 204.97) | 144.73 (103.31, 205.30) |
| Cameroon | 774.73 (671.11, 893.00) | 790.68 (684.94, 911.39) | 806.97 (699.05, 930.17) | 189.01 (134.08, 259.89) | 191.15 (135.59, 262.82) | 193.31 (137.12, 265.79) |
| Canada | 728.25 (590.37, 878.12) | 743.40 (602.65, 896.38) | 758.86 (615.18, 915.03) | 39.68 (33.09, 44.23) | 38.67 (32.25, 43.10) | 37.68 (31.42, 42.00) |
| Central African Republic | 698.41 (556.88, 869.92) | 705.74 (562.73, 879.05) | 713.15 (568.63, 888.28) | 199.08 (143.33, 261.44) | 199.24 (143.45, 261.65) | 199.40 (143.56, 261.86) |
| Chad | 720.72 (605.67, 845.88) | 734.05 (616.88, 861.53) | 747.63 (628.29, 877.46) | 170.08 (131.05, 220.86) | 172.92 (133.24, 224.55) | 175.81 (135.47, 228.30) |
| Chile | 938.73 (745.79, 1156.70) | 962.29 (764.51, 1185.73) | 986.44 (783.70, 1215.50) | 56.47 (49.00, 61.79) | 55.99 (48.58, 61.26) | 55.50 (48.16, 60.73) |
| China | 381.68 (293.34, 478.39) | 382.78 (294.19, 479.78) | 383.89 (295.04, 481.17) | 42.91 (34.05, 52.94) | 42.72 (33.90, 52.71) | 42.53 (33.75, 52.48) |
| Colombia | 631.30 (514.34, 757.55) | 632.68 (515.47, 759.22) | 634.08 (516.60, 760.89) | 66.01 (54.86, 76.72) | 64.23 (53.38, 74.65) | 62.51 (51.95, 72.65) |
| Comoros | 646.31 (555.78, 753.98) | 650.25 (559.17, 758.58) | 654.22 (562.58, 763.21) | 171.57 (117.58, 241.61) | 171.62 (117.61, 241.68) | 171.68 (117.65, 241.76) |
| Congo | 713.03 (599.15, 846.10) | 721.87 (606.58, 856.60) | 730.82 (614.11, 867.22) | 228.15 (174.38, 291.90) | 228.10 (174.34, 291.84) | 228.06 (174.31, 291.79) |
| Cook Islands | 865.79 (663.35, 1110.30) | 859.64 (658.64, 1102.42) | 853.54 (653.96, 1094.59) | 533.03 (416.92, 672.44) | 528.50 (413.38, 666.72) | 524.01 (409.87, 661.06) |
| Costa Rica | 709.35 (557.33, 854.92) | 716.23 (562.74, 863.21) | 723.18 (568.20, 871.59) | 101.25 (86.34, 113.68) | 100.26 (85.49, 112.57) | 99.27 (84.65, 111.47) |
| Côte d'Ivoire | 830.40 (695.19, 986.04) | 847.34 (709.37, 1006.15) | 864.62 (723.84, 1026.68) | 159.25 (120.74, 209.72) | 162.98 (123.57, 214.63) | 166.79 (126.46, 219.65) |
| Croatia | 656.89 (532.89, 772.60) | 659.19 (534.76, 775.31) | 661.50 (536.63, 778.02) | 103.00 (88.02, 114.95) | 103.72 (88.64, 115.76) | 104.45 (89.26, 116.57) |
| Cuba | 621.01 (501.75, 753.39) | 624.42 (504.51, 757.54) | 627.86 (507.28, 761.70) | 59.17 (50.85, 67.57) | 57.35 (49.28, 65.49) | 55.58 (47.76, 63.48) |
| Cyprus | 864.54 (761.33, 957.81) | 848.63 (747.32, 940.19) | 833.02 (733.57, 922.89) | 142.89 (116.14, 170.36) | 138.09 (112.24, 164.63) | 133.45 (108.46, 159.10) |
| Czechia | 691.43 (551.80, 814.82) | 702.15 (560.36, 827.45) | 713.03 (569.04, 840.27) | 115.49 (98.62, 131.50) | 119.18 (101.77, 135.71) | 123.00 (105.03, 140.05) |
| Democratic People's Republic of Korea | 451.18 (355.28, 558.78) | 456.55 (359.50, 565.43) | 461.98 (363.78, 572.16) | 77.61 (58.43, 104.56) | 78.50 (59.10, 105.75) | 79.39 (59.77, 106.95) |
| Democratic Republic of the Congo | 509.35 (414.91, 605.06) | 514.95 (419.48, 611.72) | 520.62 (424.09, 618.45) | 185.88 (125.96, 248.19) | 187.05 (126.75, 249.76) | 188.23 (127.55, 251.33) |
| Denmark | 343.28 (279.14, 408.02) | 350.97 (285.39, 417.16) | 358.83 (291.79, 426.50) | 57.41 (47.02, 64.05) | 57.47 (47.08, 64.13) | 57.54 (47.14, 64.21) |
| Djibouti | 558.88 (486.43, 632.62) | 567.15 (493.63, 641.99) | 575.55 (500.93, 651.49) | 155.51 (105.31, 223.57) | 156.57 (106.03, 225.09) | 157.63 (106.75, 226.62) |
| Dominica | 1199.47 (972.62, 1436.09) | 1207.51 (979.13, 1445.71) | 1215.60 (985.69, 1455.40) | 422.86 (369.54, 488.34) | 422.14 (368.91, 487.51) | 421.42 (368.29, 486.68) |
| Dominican Republic | 804.55 (657.69, 976.47) | 815.74 (666.83, 990.04) | 827.07 (676.10, 1003.80) | 150.98 (122.28, 187.52) | 152.65 (123.64, 189.60) | 154.35 (125.01, 191.71) |
| Ecuador | 1002.06 (841.39, 1203.25) | 1020.59 (856.96, 1225.51) | 1039.47 (872.81, 1248.19) | 148.23 (121.79, 180.81) | 149.09 (122.50, 181.86) | 149.95 (123.21, 182.91) |
| Egypt | 1094.04 (867.86, 1362.22) | 1136.60 (901.62, 1415.22) | 1180.81 (936.69, 1470.27) | 246.13 (201.21, 299.05) | 253.25 (207.03, 307.69) | 260.57 (213.01, 316.59) |
| El Salvador | 592.92 (480.92, 703.21) | 602.47 (488.66, 714.53) | 612.17 (496.53, 726.04) | 242.12 (194.78, 305.35) | 248.05 (199.55, 312.83) | 254.13 (204.44, 320.49) |
| Equatorial Guinea | 819.29 (695.10, 953.30) | 839.85 (712.55, 977.23) | 860.93 (730.44, 1001.75) | 188.61 (120.55, 278.35) | 189.23 (120.95, 279.27) | 189.85 (121.34, 280.19) |
| Eritrea | 645.43 (562.74, 737.07) | 654.15 (570.33, 747.02) | 662.98 (578.03, 757.10) | 204.96 (148.28, 264.59) | 206.52 (149.41, 266.60) | 208.09 (150.54, 268.63) |
| Estonia | 354.04 (291.15, 439.45) | 358.68 (294.96, 445.20) | 363.38 (298.82, 451.04) | 60.73 (51.11, 69.03) | 62.19 (52.34, 70.70) | 63.69 (53.60, 72.40) |
| Eswatini | 1555.57 (1395.48, 1719.98) | 1593.68 (1429.67, 1762.12) | 1632.73 (1464.70, 1805.29) | 520.91 (360.51, 711.84) | 529.67 (366.56, 723.80) | 538.56 (372.72, 735.96) |
| Ethiopia | 546.16 (471.80, 635.60) | 544.79 (470.62, 634.01) | 543.43 (469.44, 632.43) | 138.83 (114.61, 167.21) | 135.33 (111.72, 163.00) | 131.92 (108.91, 158.89) |
| Fiji | 1419.69 (1048.93, 1753.37) | 1425.22 (1053.02, 1760.21) | 1430.78 (1057.13, 1767.08) | 1107.87 (871.36, 1391.85) | 1122.05 (882.51, 1409.66) | 1136.41 (893.81, 1427.71) |
| Finland | 425.89 (355.35, 510.08) | 428.66 (357.66, 513.39) | 431.45 (359.99, 516.73) | 23.22 (18.35, 26.56) | 22.62 (17.88, 25.87) | 22.03 (17.41, 25.19) |
| France | 271.77 (221.04, 327.07) | 272.15 (221.35, 327.53) | 272.53 (221.66, 327.99) | 53.46 (42.51, 60.16) | 53.26 (42.35, 59.93) | 53.06 (42.18, 59.70) |
| Gabon | 793.08 (686.62, 925.52) | 805.06 (696.99, 939.50) | 817.22 (707.51, 953.68) | 217.38 (149.93, 285.51) | 217.54 (150.03, 285.71) | 217.69 (150.14, 285.91) |
| Gambia | 749.48 (629.40, 891.87) | 764.02 (641.62, 909.17) | 778.84 (654.06, 926.81) | 205.05 (152.89, 272.34) | 209.36 (156.10, 278.06) | 213.76 (159.38, 283.90) |
| Georgia | 413.28 (322.93, 507.96) | 420.48 (328.54, 516.80) | 427.79 (334.26, 525.79) | 87.57 (75.93, 99.15) | 90.46 (78.44, 102.43) | 93.44 (81.02, 105.81) |
| Germany | 423.14 (357.52, 516.60) | 425.98 (359.92, 520.06) | 428.83 (362.33, 523.55) | 60.92 (47.16, 69.15) | 59.67 (46.20, 67.73) | 58.45 (45.25, 66.34) |
| Ghana | 735.61 (621.83, 855.36) | 748.48 (632.72, 870.33) | 761.58 (643.79, 885.56) | 161.27 (123.16, 214.19) | 163.43 (124.81, 217.06) | 165.62 (126.49, 219.97) |
| Greece | 328.66 (255.11, 398.01) | 326.98 (253.81, 395.98) | 325.32 (252.52, 393.96) | 44.09 (36.65, 49.48) | 43.91 (36.50, 49.28) | 43.73 (36.35, 49.08) |
| Greenland | 465.90 (394.55, 552.00) | 475.73 (402.87, 563.65) | 485.77 (411.38, 575.54) | 25.47 (20.71, 31.63) | 24.61 (20.01, 30.56) | 23.78 (19.33, 29.52) |
| Grenada | 1107.44 (881.37, 1332.60) | 1120.40 (891.68, 1348.19) | 1133.51 (902.11, 1363.97) | 407.25 (361.37, 448.28) | 403.42 (357.97, 444.07) | 399.63 (354.61, 439.89) |
| Guam | 536.68 (433.25, 645.67) | 533.73 (430.86, 642.12) | 530.79 (428.49, 638.58) | 86.34 (69.90, 102.81) | 83.87 (67.90, 99.87) | 81.47 (65.96, 97.01) |
| Guatemala | 820.92 (676.42, 973.09) | 836.02 (688.87, 991.00) | 851.40 (701.54, 1009.23) | 330.53 (288.70, 375.83) | 341.90 (298.63, 388.76) | 353.66 (308.91, 402.13) |
| Guinea | 693.15 (606.77, 801.59) | 702.23 (614.72, 812.09) | 711.43 (622.77, 822.73) | 206.50 (158.36, 262.02) | 209.91 (160.98, 266.34) | 213.37 (163.63, 270.74) |
| Guinea-Bissau | 899.55 (762.55, 1064.96) | 917.91 (778.11, 1086.69) | 936.63 (793.98, 1108.86) | 213.91 (161.15, 278.29) | 217.17 (163.60, 282.52) | 220.47 (166.09, 286.82) |
| Guyana | 1059.24 (789.74, 1359.30) | 1059.98 (790.29, 1360.26) | 1060.72 (790.84, 1361.21) | 386.90 (307.46, 482.42) | 385.05 (305.98, 480.11) | 383.20 (304.51, 477.80) |
| Haiti | 1130.71 (853.27, 1436.53) | 1134.78 (856.34, 1441.70) | 1138.86 (859.43, 1446.89) | 466.81 (348.47, 626.48) | 466.11 (347.95, 625.54) | 465.41 (347.43, 624.60) |
| Honduras | 710.24 (567.07, 875.97) | 722.03 (576.48, 890.51) | 734.02 (586.05, 905.30) | 160.85 (116.06, 219.32) | 165.24 (119.23, 225.30) | 169.75 (122.49, 231.45) |
| Hungary | 583.98 (483.90, 688.10) | 587.72 (486.99, 692.51) | 591.48 (490.11, 696.94) | 76.89 (66.12, 85.51) | 77.17 (66.35, 85.82) | 77.45 (66.59, 86.13) |
| Iceland | 336.29 (268.19, 398.56) | 341.87 (272.64, 405.18) | 347.55 (277.17, 411.91) | 20.07 (15.60, 22.96) | 19.77 (15.36, 22.61) | 19.46 (15.13, 22.26) |
| India | 434.98 (358.55, 519.45) | 439.07 (361.93, 524.33) | 443.20 (365.33, 529.26) | 139.81 (121.07, 160.84) | 142.13 (123.08, 163.51) | 144.49 (125.12, 166.23) |
| Indonesia | 771.93 (640.63, 905.01) | 798.10 (662.34, 935.69) | 825.15 (684.80, 967.41) | 125.19 (97.87, 158.77) | 126.25 (98.70, 160.11) | 127.32 (99.54, 161.48) |
| Iran (Islamic Republic of) | 870.27 (703.15, 1068.03) | 894.55 (722.76, 1097.83) | 919.51 (742.93, 1128.46) | 107.70 (93.61, 120.05) | 110.40 (95.95, 123.05) | 113.16 (98.35, 126.12) |
| Iraq | 1109.78 (840.13, 1387.90) | 1129.87 (855.33, 1413.02) | 1150.32 (870.81, 1438.60) | 192.35 (149.20, 238.60) | 192.12 (149.02, 238.31) | 191.89 (148.84, 238.02) |
| Ireland | 355.86 (291.34, 426.28) | 360.13 (294.83, 431.39) | 364.45 (298.37, 436.57) | 24.92 (19.69, 28.86) | 24.29 (19.19, 28.13) | 23.67 (18.70, 27.41) |
| Israel | 697.85 (613.80, 798.53) | 704.00 (619.20, 805.56) | 710.19 (624.65, 812.65) | 115.60 (92.32, 130.52) | 114.89 (91.75, 129.71) | 114.17 (91.18, 128.91) |
| Italy | 452.50 (364.00, 551.56) | 450.87 (362.69, 549.58) | 449.25 (361.38, 547.60) | 79.64 (60.93, 89.94) | 78.66 (60.18, 88.84) | 77.69 (59.44, 87.74) |
| Jamaica | 1150.49 (1008.18, 1301.60) | 1160.39 (1016.85, 1312.79) | 1170.37 (1025.60, 1324.08) | 452.85 (366.80, 557.58) | 451.36 (365.59, 555.74) | 449.87 (364.38, 553.90) |
| Japan | 488.43 (408.48, 580.68) | 489.31 (409.21, 581.73) | 490.19 (409.95, 582.77) | 16.66 (11.79, 19.53) | 16.27 (11.51, 19.07) | 15.89 (11.24, 18.63) |
| Jordan | 1520.89 (1198.23, 1817.38) | 1523.48 (1200.26, 1820.47) | 1526.07 (1202.31, 1823.57) | 164.54 (133.59, 200.20) | 160.38 (130.21, 195.14) | 156.32 (126.91, 190.20) |
| Kazakhstan | 654.37 (498.41, 782.83) | 674.07 (513.41, 806.39) | 694.36 (528.87, 830.67) | 43.85 (35.80, 52.21) | 43.50 (35.51, 51.79) | 43.15 (35.23, 51.38) |
| Kenya | 408.70 (355.58, 466.57) | 409.97 (356.69, 468.02) | 411.24 (357.79, 469.47) | 124.77 (93.63, 159.13) | 126.16 (94.67, 160.89) | 127.56 (95.72, 162.68) |
| Kiribati | 908.38 (718.60, 1150.68) | 908.38 (718.60, 1150.68) | 908.38 (718.60, 1150.68) | 795.13 (625.87, 993.91) | 802.29 (631.50, 1002.85) | 809.51 (637.19, 1011.88) |
| Kuwait | 1601.36 (1204.20, 2057.43) | 1636.92 (1230.93, 2103.10) | 1673.25 (1258.26, 2149.79) | 83.88 (68.14, 97.52) | 82.88 (67.33, 96.35) | 81.88 (66.52, 95.20) |
| Kyrgyzstan | 368.23 (285.31, 479.76) | 377.11 (292.18, 491.32) | 386.19 (299.22, 503.16) | 37.13 (31.18, 43.84) | 37.06 (31.13, 43.77) | 37.00 (31.07, 43.69) |
| Lao People's Democratic Republic | 1329.34 (1094.97, 1553.30) | 1363.50 (1123.11, 1593.22) | 1398.54 (1151.97, 1634.17) | 188.86 (144.36, 237.58) | 188.59 (144.16, 237.24) | 188.33 (143.95, 236.91) |
| Latvia | 369.32 (287.82, 464.47) | 376.30 (293.26, 473.25) | 383.42 (298.81, 482.19) | 81.17 (70.77, 90.74) | 83.75 (73.02, 93.62) | 86.41 (75.35, 96.60) |
| Lebanon | 846.43 (674.48, 1020.56) | 855.32 (681.56, 1031.28) | 864.30 (688.72, 1042.10) | 125.29 (99.87, 153.21) | 125.13 (99.74, 153.01) | 124.96 (99.61, 152.81) |
| Lesotho | 1216.11 (1094.81, 1349.76) | 1255.52 (1130.29, 1393.50) | 1296.19 (1166.91, 1438.65) | 473.78 (336.10, 632.44) | 492.31 (349.24, 657.17) | 511.56 (362.90, 682.86) |
| Liberia | 728.65 (614.49, 858.62) | 740.38 (624.39, 872.44) | 752.30 (634.44, 886.49) | 195.18 (144.84, 257.92) | 197.94 (146.88, 261.56) | 200.73 (148.95, 265.24) |
| Libya | 963.26 (715.47, 1230.62) | 987.92 (733.78, 1262.13) | 1013.21 (752.57, 1294.44) | 119.61 (89.10, 161.87) | 123.10 (91.70, 166.59) | 126.69 (94.37, 171.46) |
| Lithuania | 296.29 (239.41, 374.36) | 299.58 (242.07, 378.51) | 302.91 (244.76, 382.71) | 49.15 (42.84, 55.35) | 50.25 (43.80, 56.58) | 51.37 (44.78, 57.84) |
| Luxembourg | 403.19 (332.99, 483.35) | 406.49 (335.72, 487.31) | 409.82 (338.47, 491.31) | 37.20 (30.53, 42.76) | 36.61 (30.05, 42.08) | 36.03 (29.57, 41.42) |
| Madagascar | 489.80 (423.28, 563.82) | 493.62 (426.59, 568.22) | 497.47 (429.91, 572.65) | 131.77 (96.19, 176.58) | 131.29 (95.85, 175.95) | 130.82 (95.50, 175.31) |
| Malawi | 457.15 (403.16, 512.42) | 458.24 (404.12, 513.65) | 459.34 (405.09, 514.88) | 159.30 (123.36, 203.54) | 158.86 (123.01, 202.97) | 158.41 (122.67, 202.41) |
| Malaysia | 1382.97 (1183.07, 1656.79) | 1407.44 (1204.01, 1686.12) | 1432.36 (1225.32, 1715.96) | 94.16 (80.28, 110.61) | 92.26 (78.67, 108.38) | 90.41 (77.09, 106.21) |
| Maldives | 1063.75 (890.73, 1256.03) | 1079.07 (903.56, 1274.12) | 1094.61 (916.57, 1292.47) | 83.67 (66.83, 102.96) | 81.51 (65.10, 100.31) | 79.41 (63.42, 97.72) |
| Mali | 1195.74 (962.23, 1429.86) | 1219.78 (981.57, 1458.60) | 1244.29 (1001.30, 1487.92) | 202.48 (154.93, 258.97) | 205.13 (156.96, 262.37) | 207.82 (159.01, 265.80) |
| Malta | 570.50 (466.72, 671.41) | 574.49 (469.99, 676.11) | 578.51 (473.28, 680.84) | 100.16 (80.70, 115.13) | 98.28 (79.19, 112.97) | 96.43 (77.70, 110.84) |
| Marshall Islands | 818.48 (606.05, 1085.03) | 817.50 (605.32, 1083.72) | 816.52 (604.59, 1082.42) | 894.97 (563.50, 1235.91) | 905.44 (570.09, 1250.37) | 916.03 (576.76, 1265.00) |
| Mauritania | 591.19 (522.27, 669.59) | 597.34 (527.70, 676.55) | 603.55 (533.19, 683.59) | 171.96 (123.83, 233.83) | 172.65 (124.33, 234.77) | 173.34 (124.83, 235.71) |
| Mauritius | 1950.71 (1702.04, 2211.02) | 2004.94 (1749.35, 2272.48) | 2060.68 (1797.99, 2335.66) | 454.35 (414.37, 483.67) | 471.07 (429.62, 501.47) | 488.40 (445.43, 519.92) |
| Mexico | 862.63 (705.01, 1024.94) | 855.13 (698.88, 1016.03) | 847.69 (692.80, 1007.19) | 319.24 (275.27, 362.68) | 316.69 (273.06, 359.77) | 314.15 (270.88, 356.90) |
| Micronesia (Federated States of) | 1099.46 (876.64, 1355.66) | 1108.25 (883.65, 1366.51) | 1117.12 (890.72, 1377.44) | 515.43 (395.42, 668.86) | 518.68 (397.91, 673.07) | 521.95 (400.42, 677.31) |
| Monaco | 285.91 (232.85, 348.19) | 291.11 (237.09, 354.52) | 296.41 (241.41, 360.98) | 22.08 (15.43, 28.77) | 22.01 (15.38, 28.67) | 21.93 (15.32, 28.57) |
| Mongolia | 291.84 (231.90, 358.53) | 298.43 (237.14, 366.63) | 305.18 (242.50, 374.91) | 32.01 (26.11, 38.26) | 32.36 (26.39, 38.67) | 32.71 (26.68, 39.09) |
| Montenegro | 827.77 (678.94, 973.50) | 834.89 (684.78, 981.87) | 842.07 (690.67, 990.32) | 92.74 (78.01, 110.53) | 94.34 (79.35, 112.43) | 95.96 (80.72, 114.37) |
| Morocco | 1065.96 (804.49, 1366.61) | 1088.67 (821.63, 1395.72) | 1111.86 (839.13, 1425.45) | 127.57 (91.12, 161.07) | 131.10 (93.64, 165.54) | 134.74 (96.24, 170.12) |
| Mozambique | 520.10 (451.08, 601.19) | 525.71 (455.95, 607.68) | 531.39 (460.88, 614.25) | 150.40 (106.54, 197.46) | 151.74 (107.49, 199.22) | 153.09 (108.45, 200.99) |
| Myanmar | 1476.37 (1250.11, 1732.72) | 1509.15 (1277.86, 1771.18) | 1542.65 (1306.23, 1810.50) | 246.87 (197.80, 311.54) | 245.74 (196.89, 310.11) | 244.60 (195.99, 308.68) |
| Namibia | 855.91 (732.71, 978.35) | 863.53 (739.23, 987.06) | 871.21 (745.81, 995.84) | 333.48 (238.36, 437.84) | 333.58 (238.43, 437.97) | 333.68 (238.51, 438.10) |
| Nauru | 1061.86 (813.98, 1365.18) | 1066.01 (817.16, 1370.50) | 1070.16 (820.34, 1375.85) | 644.28 (489.68, 849.33) | 649.75 (493.84, 856.55) | 655.28 (498.04, 863.83) |
| Nepal | 605.49 (475.72, 732.92) | 618.57 (486.00, 748.75) | 631.93 (496.50, 764.92) | 133.90 (99.38, 177.57) | 135.73 (100.74, 180.00) | 137.59 (102.12, 182.47) |
| Netherlands | 348.71 (292.20, 416.24) | 346.37 (290.25, 413.45) | 344.05 (288.30, 410.68) | 50.99 (41.19, 57.69) | 49.48 (39.98, 55.98) | 48.01 (38.79, 54.33) |
| New Zealand | 487.18 (432.33, 546.83) | 497.46 (441.45, 558.37) | 507.96 (450.77, 570.15) | 35.94 (29.68, 40.38) | 35.19 (29.06, 39.55) | 34.46 (28.46, 38.73) |
| Nicaragua | 860.82 (678.51, 1047.15) | 870.80 (686.38, 1059.30) | 880.90 (694.34, 1071.59) | 155.54 (127.06, 189.07) | 156.68 (127.98, 190.45) | 157.82 (128.92, 191.84) |
| Niger | 746.38 (614.47, 926.17) | 760.79 (626.33, 944.05) | 775.47 (638.41, 962.27) | 110.73 (76.25, 156.61) | 111.94 (77.09, 158.33) | 113.17 (77.94, 160.07) |
| Nigeria | 629.64 (536.51, 727.06) | 636.95 (542.74, 735.50) | 644.34 (549.03, 744.03) | 133.47 (99.85, 179.20) | 132.71 (99.28, 178.18) | 131.95 (98.72, 177.16) |
| Niue | 822.69 (622.27, 1072.41) | 820.96 (620.96, 1070.16) | 819.24 (619.66, 1067.91) | 663.48 (476.09, 836.72) | 671.31 (481.70, 846.59) | 679.23 (487.39, 856.58) |
| North Macedonia | 1147.78 (929.68, 1366.83) | 1165.46 (944.00, 1387.88) | 1183.41 (958.54, 1409.25) | 170.18 (136.21, 208.65) | 173.06 (138.51, 212.17) | 175.98 (140.85, 215.76) |
| Northern Mariana Islands | 733.78 (588.55, 889.33) | 740.24 (593.73, 897.15) | 746.75 (598.95, 905.05) | 219.72 (185.94, 249.10) | 217.41 (183.99, 246.49) | 215.13 (182.06, 243.90) |
| Norway | 348.79 (285.01, 420.41) | 348.89 (285.10, 420.54) | 349.00 (285.18, 420.66) | 33.81 (27.10, 37.52) | 33.52 (26.87, 37.20) | 33.23 (26.63, 36.87) |
| Oman | 976.91 (790.24, 1190.06) | 999.57 (808.57, 1217.67) | 1022.76 (827.33, 1245.92) | 171.04 (136.71, 209.97) | 170.37 (136.18, 209.15) | 169.71 (135.64, 208.34) |
| Pakistan | 852.99 (709.81, 1005.87) | 883.78 (735.43, 1042.18) | 915.68 (761.98, 1079.80) | 229.46 (169.90, 300.81) | 232.60 (172.23, 304.93) | 235.79 (174.59, 309.11) |
| Palau | 1051.59 (808.33, 1355.79) | 1053.38 (809.71, 1358.09) | 1055.17 (811.08, 1360.40) | 459.47 (352.28, 574.52) | 462.41 (354.53, 578.19) | 465.37 (356.80, 581.90) |
| Palestine | 1353.64 (1097.27, 1598.41) | 1377.74 (1116.80, 1626.86) | 1402.26 (1136.68, 1655.82) | 234.90 (196.45, 277.29) | 235.77 (197.17, 278.32) | 236.64 (197.90, 279.34) |
| Panama | 721.94 (579.80, 900.75) | 728.72 (585.25, 909.21) | 735.57 (590.75, 917.76) | 157.13 (123.58, 187.61) | 157.96 (124.24, 188.60) | 158.80 (124.90, 189.60) |
| Papua New Guinea | 765.21 (613.95, 935.63) | 776.61 (623.10, 949.57) | 788.18 (632.39, 963.72) | 279.98 (217.20, 351.28) | 281.02 (218.00, 352.58) | 282.06 (218.81, 353.88) |
| Paraguay | 907.84 (726.00, 1079.55) | 925.08 (739.79, 1100.06) | 942.66 (753.85, 1120.97) | 299.31 (228.88, 378.53) | 307.24 (234.95, 388.57) | 315.38 (241.17, 398.86) |
| Peru | 604.56 (524.77, 697.89) | 616.16 (534.84, 711.29) | 627.99 (545.11, 724.95) | 92.88 (70.92, 122.24) | 93.42 (71.33, 122.95) | 93.96 (71.75, 123.66) |
| Philippines | 1003.73 (863.35, 1162.80) | 1009.45 (868.28, 1169.43) | 1015.20 (873.22, 1176.09) | 175.36 (142.60, 213.29) | 177.62 (144.44, 216.05) | 179.91 (146.30, 218.83) |
| Poland | 654.12 (548.75, 785.10) | 661.39 (554.84, 793.81) | 668.73 (561.00, 802.62) | 75.08 (64.33, 83.19) | 75.19 (64.42, 83.31) | 75.29 (64.51, 83.43) |
| Portugal | 684.51 (564.24, 838.20) | 692.79 (571.06, 848.34) | 701.18 (577.97, 858.61) | 103.00 (80.84, 117.31) | 101.96 (80.02, 116.12) | 100.93 (79.21, 114.95) |
| Puerto Rico | 867.36 (681.20, 1071.63) | 864.41 (678.89, 1067.99) | 861.47 (676.58, 1064.36) | 227.15 (185.76, 267.99) | 224.36 (183.47, 264.69) | 221.60 (181.22, 261.44) |
| Qatar | 2725.04 (2097.64, 3129.25) | 2762.10 (2126.17, 3171.81) | 2799.67 (2155.09, 3214.95) | 238.24 (191.96, 298.16) | 238.10 (191.84, 297.99) | 237.96 (191.73, 297.81) |
| Republic of Korea | 962.70 (807.76, 1108.92) | 984.07 (825.69, 1133.54) | 1005.91 (844.02, 1158.70) | 56.32 (42.69, 67.39) | 55.34 (41.95, 66.21) | 54.37 (41.21, 65.06) |
| Republic of Moldova | 446.85 (346.51, 551.32) | 452.44 (350.84, 558.21) | 458.09 (355.22, 565.19) | 43.41 (37.91, 49.11) | 43.18 (37.70, 48.84) | 42.95 (37.50, 48.58) |
| Romania | 367.64 (292.36, 450.27) | 370.47 (294.61, 453.73) | 373.32 (296.88, 457.23) | 40.29 (35.66, 45.72) | 40.42 (35.77, 45.86) | 40.54 (35.88, 46.00) |
| Russian Federation | 448.99 (373.25, 539.23) | 457.47 (380.31, 549.42) | 466.12 (387.50, 559.80) | 103.01 (92.52, 112.20) | 106.65 (95.79, 116.16) | 110.41 (99.17, 120.26) |
| Rwanda | 470.39 (415.18, 535.65) | 471.01 (415.72, 536.34) | 471.62 (416.26, 537.04) | 138.20 (81.90, 212.53) | 135.05 (80.04, 207.69) | 131.97 (78.21, 202.95) |
| Saint Kitts and Nevis | 1060.35 (850.46, 1298.47) | 1071.38 (859.30, 1311.97) | 1082.52 (868.24, 1325.62) | 202.07 (171.01, 234.79) | 197.33 (166.99, 229.28) | 192.69 (163.07, 223.89) |
| Saint Lucia | 1115.21 (917.48, 1327.99) | 1107.74 (911.33, 1319.09) | 1100.32 (905.23, 1310.25) | 321.35 (269.22, 376.89) | 311.10 (260.63, 364.87) | 301.17 (252.32, 353.23) |
| Saint Vincent and the Grenadines | 1154.01 (918.75, 1427.89) | 1149.62 (915.26, 1422.46) | 1145.25 (911.79, 1417.06) | 363.56 (322.13, 410.20) | 355.74 (315.21, 401.38) | 348.09 (308.43, 392.75) |
| Samoa | 813.36 (630.02, 1039.00) | 814.66 (631.03, 1040.67) | 815.96 (632.04, 1042.33) | 485.11 (370.18, 627.07) | 492.83 (376.06, 637.04) | 500.66 (382.04, 647.17) |
| San Marino | 366.86 (301.84, 449.37) | 371.92 (306.01, 455.58) | 377.06 (310.23, 461.86) | 30.07 (19.14, 41.80) | 29.68 (18.89, 41.26) | 29.29 (18.65, 40.72) |
| Sao Tome and Principe | 723.67 (586.72, 871.86) | 736.26 (596.93, 887.03) | 749.07 (607.31, 902.47) | 61.29 (47.68, 75.42) | 61.69 (47.99, 75.91) | 62.09 (48.30, 76.40) |
| Saudi Arabia | 1067.51 (842.63, 1321.29) | 1087.26 (858.22, 1345.73) | 1107.37 (874.09, 1370.63) | 101.73 (80.58, 127.97) | 101.11 (80.09, 127.19) | 100.49 (79.60, 126.41) |
| Senegal | 1144.51 (954.50, 1323.14) | 1170.83 (976.45, 1353.57) | 1197.76 (998.91, 1384.71) | 220.84 (163.14, 288.89) | 224.86 (166.11, 294.15) | 228.95 (169.13, 299.51) |
| Serbia | 844.23 (698.77, 999.01) | 849.29 (702.97, 1005.01) | 854.39 (707.18, 1011.04) | 132.20 (110.14, 158.08) | 133.44 (111.17, 159.57) | 134.69 (112.22, 161.07) |
| Seychelles | 1801.09 (1546.28, 2071.68) | 1858.54 (1595.60, 2137.77) | 1917.83 (1646.50, 2205.96) | 129.25 (109.57, 150.01) | 131.48 (111.47, 152.60) | 133.76 (113.40, 155.24) |
| Sierra Leone | 744.43 (619.09, 895.40) | 759.02 (631.22, 912.95) | 773.90 (643.59, 930.85) | 161.37 (123.99, 208.83) | 164.54 (126.42, 212.92) | 167.76 (128.90, 217.10) |
| Singapore | 692.99 (540.95, 833.68) | 699.09 (545.71, 841.02) | 705.24 (550.51, 848.42) | 10.19 (8.30, 11.54) | 9.44 (7.69, 10.70) | 8.75 (7.13, 9.91) |
| Slovakia | 516.71 (422.16, 614.64) | 520.63 (425.37, 619.32) | 524.59 (428.60, 624.02) | 47.73 (37.52, 58.18) | 47.19 (37.09, 57.52) | 46.66 (36.67, 56.87) |
| Slovenia | 502.25 (407.39, 591.63) | 498.68 (404.50, 587.43) | 495.14 (401.63, 583.26) | 50.18 (41.03, 58.20) | 48.86 (39.96, 56.67) | 47.58 (38.91, 55.19) |
| Solomon Islands | 971.97 (841.10, 1112.92) | 994.23 (860.36, 1138.40) | 1016.99 (880.06, 1164.47) | 446.74 (340.81, 572.12) | 452.01 (344.83, 578.88) | 457.35 (348.90, 585.71) |
| Somalia | 577.13 (496.98, 649.00) | 583.82 (502.74, 656.53) | 590.60 (508.57, 664.14) | 199.41 (144.15, 260.55) | 200.23 (144.74, 261.62) | 201.05 (145.34, 262.69) |
| South Africa | 1273.38 (1120.06, 1443.51) | 1303.82 (1146.83, 1478.01) | 1334.98 (1174.24, 1513.34) | 385.28 (353.38, 415.05) | 394.52 (361.86, 425.02) | 403.99 (370.55, 435.22) |
| South Sudan | 485.05 (424.70, 555.24) | 487.14 (426.53, 557.62) | 489.23 (428.36, 560.02) | 183.80 (130.21, 239.08) | 183.62 (130.08, 238.84) | 183.44 (129.95, 238.60) |
| Spain | 674.20 (572.11, 770.38) | 678.58 (575.83, 775.38) | 682.99 (579.57, 780.42) | 60.08 (45.50, 69.67) | 58.80 (44.52, 68.18) | 57.54 (43.57, 66.72) |
| Sri Lanka | 1953.55 (1676.36, 2256.52) | 1991.45 (1708.88, 2300.30) | 2030.09 (1742.03, 2344.93) | 235.31 (163.00, 311.93) | 240.21 (166.39, 318.42) | 245.20 (169.85, 325.04) |
| Sudan | 819.88 (643.96, 998.77) | 836.77 (657.22, 1019.35) | 854.01 (670.76, 1040.34) | 95.59 (72.45, 129.74) | 96.90 (73.44, 131.52) | 98.23 (74.45, 133.32) |
| Suriname | 900.97 (698.13, 1130.12) | 908.72 (704.13, 1139.84) | 916.53 (710.19, 1149.64) | 201.09 (161.00, 247.75) | 201.83 (161.59, 248.67) | 202.58 (162.19, 249.59) |
| Sweden | 395.00 (315.77, 482.96) | 398.79 (318.80, 487.59) | 402.62 (321.86, 492.27) | 53.16 (41.90, 61.82) | 53.06 (41.82, 61.70) | 52.96 (41.75, 61.59) |
| Switzerland | 577.44 (477.22, 674.79) | 582.00 (480.99, 680.12) | 586.60 (484.79, 685.50) | 36.83 (27.85, 42.71) | 35.53 (26.87, 41.21) | 34.28 (25.92, 39.76) |
| Syrian Arab Republic | 1044.30 (830.15, 1278.35) | 1064.66 (846.34, 1303.28) | 1085.42 (862.85, 1328.69) | 84.75 (63.85, 110.52) | 84.01 (63.29, 109.56) | 83.28 (62.74, 108.61) |
| Taiwan (Province of China) | 748.13 (605.03, 906.12) | 751.27 (607.57, 909.93) | 754.43 (610.12, 913.75) | 121.54 (100.45, 136.31) | 118.14 (97.64, 132.49) | 114.83 (94.90, 128.79) |
| Tajikistan | 443.56 (344.24, 548.39) | 458.46 (355.81, 566.82) | 473.87 (367.76, 585.86) | 66.05 (49.19, 83.56) | 66.35 (49.42, 83.94) | 66.65 (49.65, 84.33) |
| Thailand | 991.27 (816.13, 1184.74) | 1009.91 (831.47, 1207.01) | 1028.89 (847.11, 1229.70) | 122.11 (92.67, 156.27) | 121.07 (91.89, 154.94) | 120.04 (91.10, 153.62) |
| Timor-Leste | 1212.78 (1016.20, 1427.38) | 1257.29 (1053.50, 1479.77) | 1303.43 (1092.16, 1534.08) | 131.26 (101.93, 175.07) | 132.53 (102.92, 176.77) | 133.82 (103.92, 178.48) |
| Togo | 608.43 (522.72, 702.90) | 619.68 (532.39, 715.90) | 631.15 (542.24, 729.14) | 131.75 (97.34, 175.51) | 133.33 (98.51, 177.62) | 134.93 (99.69, 179.75) |
| Tokelau | 768.41 (608.82, 956.00) | 765.65 (606.62, 952.56) | 762.89 (604.44, 949.13) | 506.72 (380.06, 667.50) | 509.71 (382.30, 671.44) | 512.72 (384.56, 675.40) |
| Tonga | 815.84 (637.44, 1030.52) | 812.17 (634.57, 1025.88) | 808.51 (631.71, 1021.27) | 637.80 (483.25, 812.24) | 645.45 (489.05, 821.99) | 653.20 (494.92, 831.85) |
| Trinidad and Tobago | 1171.05 (898.09, 1443.97) | 1176.79 (902.49, 1451.04) | 1182.55 (906.91, 1458.15) | 451.26 (354.26, 556.87) | 443.82 (348.41, 547.69) | 436.50 (342.66, 538.65) |
| Tunisia | 965.65 (768.19, 1187.44) | 988.72 (786.55, 1215.82) | 1012.36 (805.35, 1244.88) | 77.11 (56.83, 101.91) | 78.63 (57.95, 103.92) | 80.18 (59.10, 105.96) |
| Türkiye | 786.70 (649.09, 948.58) | 794.73 (655.71, 958.25) | 802.83 (662.40, 968.03) | 136.75 (108.07, 168.12) | 136.67 (108.00, 168.02) | 136.59 (107.94, 167.92) |
| Turkmenistan | 321.71 (232.93, 398.86) | 328.98 (238.20, 407.88) | 336.41 (243.58, 417.10) | 98.97 (76.60, 128.13) | 100.63 (77.89, 130.28) | 102.32 (79.20, 132.47) |
| Tuvalu | 1000.51 (815.57, 1184.40) | 1009.51 (822.91, 1195.06) | 1018.60 (830.31, 1205.82) | 524.03 (406.67, 681.45) | 527.54 (409.40, 686.01) | 531.08 (412.14, 690.61) |
| Uganda | 472.25 (416.76, 530.91) | 474.80 (419.01, 533.78) | 477.37 (421.27, 536.66) | 162.10 (108.01, 225.83) | 162.22 (108.08, 225.98) | 162.33 (108.16, 226.14) |
| Ukraine | 301.73 (239.16, 374.98) | 305.65 (242.27, 379.85) | 309.63 (245.42, 384.79) | 15.23 (10.48, 21.09) | 14.91 (10.26, 20.64) | 14.59 (10.04, 20.20) |
| United Arab Emirates | 1720.26 (1384.68, 2081.87) | 1731.10 (1393.41, 2094.98) | 1742.01 (1402.19, 2108.18) | 141.32 (112.06, 176.81) | 141.22 (111.98, 176.69) | 141.12 (111.90, 176.56) |
| United Kingdom | 406.31 (334.83, 484.36) | 409.31 (337.31, 487.95) | 412.34 (339.81, 491.56) | 27.35 (22.59, 29.73) | 26.79 (22.13, 29.12) | 26.24 (21.68, 28.53) |
| United Republic of Tanzania | 512.53 (456.11, 581.48) | 519.19 (462.04, 589.04) | 525.94 (468.05, 596.69) | 154.77 (122.39, 200.83) | 155.57 (123.03, 201.87) | 156.38 (123.67, 202.92) |
| United States of America | 918.27 (806.58, 1036.77) | 956.01 (839.73, 1079.38) | 995.30 (874.24, 1123.74) | 53.58 (45.96, 57.95) | 52.36 (44.91, 56.63) | 51.16 (43.88, 55.34) |
| United States Virgin Islands | 1017.18 (807.62, 1238.69) | 1018.00 (808.27, 1239.69) | 1018.81 (808.91, 1240.68) | 136.62 (106.78, 171.12) | 134.01 (104.74, 167.86) | 131.45 (102.74, 164.65) |
| Uruguay | 570.38 (461.88, 686.29) | 580.76 (470.29, 698.78) | 591.33 (478.85, 711.50) | 94.99 (80.82, 104.79) | 94.93 (80.77, 104.72) | 94.88 (80.73, 104.66) |
| Uzbekistan | 668.93 (482.56, 805.87) | 699.83 (504.85, 843.10) | 732.17 (528.18, 882.05) | 120.08 (100.55, 140.57) | 124.06 (103.89, 145.23) | 128.18 (107.34, 150.06) |
| Vanuatu | 854.11 (694.53, 1049.17) | 867.01 (705.02, 1065.01) | 880.10 (715.67, 1081.09) | 407.03 (320.43, 494.39) | 409.92 (322.71, 497.90) | 412.83 (325.00, 501.43) |
| Venezuela (Bolivarian Republic of) | 717.91 (579.49, 882.11) | 724.59 (584.88, 890.31) | 731.33 (590.32, 898.59) | 201.41 (160.85, 250.77) | 201.45 (160.88, 250.82) | 201.49 (160.91, 250.87) |
| Viet Nam | 1089.47 (963.54, 1247.55) | 1109.51 (981.27, 1270.50) | 1129.93 (999.33, 1293.88) | 182.55 (144.99, 226.39) | 183.35 (145.63, 227.39) | 184.16 (146.27, 228.39) |
| Yemen | 677.25 (550.55, 833.88) | 700.14 (569.16, 862.07) | 723.80 (588.39, 891.20) | 75.68 (52.84, 114.85) | 76.30 (53.27, 115.78) | 76.91 (53.70, 116.72) |
| Zambia | 653.39 (552.97, 762.37) | 657.70 (556.62, 767.40) | 662.04 (560.29, 772.46) | 150.61 (111.12, 201.00) | 149.84 (110.56, 199.98) | 149.08 (109.99, 198.96) |
| Zimbabwe | 1024.85 (902.84, 1169.52) | 1047.50 (922.79, 1195.36) | 1070.65 (943.19, 1221.78) | 369.11 (276.35, 482.56) | 379.92 (284.45, 496.70) | 391.05 (292.79, 511.26) |

**Supplementary Table 5. Comparison of EAPCs in death and mortality rates of diabetes in postmenopausal women from 1990-2021, and 2023 estimates.**

|  | **EAPC of mortality rates of T2DM** | | | **EAPCs of incidence rates of T2DM** | | |
| --- | --- | --- | --- | --- | --- | --- |
| **Location** | **EAPC 1990-2021** | **EAPC 1990-2022** | **EAPC 1990-2023** | **EAPC 1990-2021** | **EAPC 1990-2022** | **EAPC 1990-2023** |
| Afghanistan | 2.21***  (2.1 to 2.32) | 2.27***  (2.17 to 2.37) | 2.31***  (2.21 to 2.4) | 1.59***  (1.47 to 1.71) | 1.58***  (1.46 to 1.71) | 1.59***  (1.45 to 1.72) |
| Albania | 0.49**  (0.18 to 0.8) | 0.47**  (0.14 to 0.8) | 0.45*  (0.1 to 0.81) | 0.93***  (0.83 to 1.04) | 0.96***  (0.85 to 1.07) | 0.98***  (0.87 to 1.09) |
| Algeria | 2.32***  (2.18 to 2.47) | 2.38***  (2.24 to 2.52) | 2.43***  (2.3 to 2.56) | 1.9***  (1.85 to 1.95) | 1.91***  (1.86 to 1.96) | 1.91***  (1.85 to 1.96) |
| American Samoa | 1.24***  (0.81 to 1.67) | 1.33***  (0.88 to 1.77) | 1.39***  (0.92 to 1.86) | -0.28***  (-0.41 to -0.16) | -0.3***  (-0.43 to -0.17) | -0.34***  (-0.47 to -0.21) |
| Andorra | 0.64***  (0.33 to 0.96) | 0.8***  (0.53 to 1.07) | 0.99***  (0.81 to 1.17) | 1.74***  (1.62 to 1.86) | 1.7***  (1.58 to 1.82) | 1.66***  (1.54 to 1.77) |
| Angola | -0.11*  (-0.21 to -0.02) | -0.13*  (-0.23 to -0.04) | -0.16**  (-0.25 to -0.06) | 1.12***  (1.06 to 1.19) | 1.13***  (1.07 to 1.2) | 1.17***  (1.11 to 1.23) |
| Antigua and Barbuda | -1.77***  (-2.05 to -1.49) | -1.74***  (-2.04 to -1.44) | -1.68***  (-2 to -1.37) | 1.2***  (1.09 to 1.31) | 1.22***  (1.11 to 1.34) | 1.28***  (1.18 to 1.38) |
| Argentina | -1.34***  (-1.62 to -1.07) | -1.28***  (-1.56 to -0.99) | -1.23***  (-1.52 to -0.93) | 0.87***  (0.79 to 0.96) | 0.82***  (0.76 to 0.89) | 0.8***  (0.74 to 0.86) |
| Armenia | -1.16  (-2.51 to 0.2) | -0.79  (-2.16 to 0.6) | -0.36  (-1.73 to 1.04) | 0.28***  (0.15 to 0.42) | 0.31***  (0.17 to 0.45) | 0.35***  (0.21 to 0.49) |
| Australia | -0.98***  (-1.36 to -0.6) | -0.93***  (-1.33 to -0.53) | -0.84***  (-1.25 to -0.42) | 1.47***  (1.42 to 1.52) | 1.47***  (1.42 to 1.53) | 1.49***  (1.43 to 1.54) |
| Austria | -0.57*  (-1.09 to -0.05) | -0.48  (-1.02 to 0.07) | -0.4  (-0.98 to 0.18) | 0.61***  (0.53 to 0.69) | 0.62***  (0.54 to 0.71) | 0.64***  (0.56 to 0.73) |
| Azerbaijan | 1.03*  (0.26 to 1.81) | 1.22**  (0.43 to 2.03) | 1.44**  (0.62 to 2.26) | 3.24***  (3.1 to 3.39) | 3.25***  (3.09 to 3.41) | 3.25***  (3.09 to 3.42) |
| Bahamas | -2.29***  (-2.58 to -1.99) | -2.26***  (-2.57 to -1.94) | -2.22***  (-2.55 to -1.89) | 0.86***  (0.83 to 0.9) | 0.85***  (0.81 to 0.88) | 0.86***  (0.82 to 0.89) |
| Bahrain | -0.43  (-1 to 0.13) | -0.37  (-0.97 to 0.23) | -0.29  (-0.92 to 0.34) | 2.12***  (1.88 to 2.37) | 2.23***  (2.01 to 2.46) | 2.35***  (2.16 to 2.54) |
| Bangladesh | 0.85***  (0.6 to 1.1) | 0.83***  (0.56 to 1.09) | 0.79***  (0.51 to 1.07) | 1.35***  (1.23 to 1.48) | 1.4***  (1.28 to 1.52) | 1.45***  (1.33 to 1.56) |
| Barbados | -1.5***  (-1.82 to -1.18) | -1.46***  (-1.8 to -1.12) | -1.42***  (-1.78 to -1.06) | 0.15***  (0.07 to 0.22) | 0.18***  (0.11 to 0.25) | 0.23***  (0.19 to 0.27) |
| Belarus | -2.41***  (-3.37 to -1.44) | -2.68***  (-3.65 to -1.71) | -3***  (-3.95 to -2.03) | 1.01***  (0.89 to 1.12) | 1***  (0.88 to 1.12) | 0.99***  (0.86 to 1.12) |
| Belgium | -2.57***  (-2.8 to -2.33) | -2.51***  (-2.76 to -2.27) | -2.5***  (-2.76 to -2.24) | 0.55***  (0.4 to 0.7) | 0.48***  (0.34 to 0.62) | 0.42***  (0.29 to 0.55) |
| Belize | -0.35  (-0.95 to 0.24) | -0.26  (-0.89 to 0.36) | -0.15  (-0.81 to 0.51) | 0.59***  (0.56 to 0.62) | 0.59***  (0.56 to 0.63) | 0.6***  (0.56 to 0.63) |
| Benin | 1.14***  (0.95 to 1.33) | 1.21***  (1.02 to 1.4) | 1.28***  (1.09 to 1.46) | 1.71***  (1.67 to 1.75) | 1.72***  (1.69 to 1.76) | 1.72***  (1.68 to 1.76) |
| Bermuda | -2.76***  (-3 to -2.52) | -2.87***  (-3.09 to -2.66) | -2.95***  (-3.16 to -2.75) | 0.43***  (0.36 to 0.49) | 0.4***  (0.34 to 0.46) | 0.39***  (0.33 to 0.45) |
| Bhutan | 1.67***  (1.62 to 1.71) | 1.68***  (1.64 to 1.73) | 1.69***  (1.64 to 1.74) | 1.83***  (1.7 to 1.97) | 1.84***  (1.7 to 1.99) | 1.86***  (1.71 to 2.01) |
| Bolivia (Plurinational State of) | 0  (-0.07 to 0.07) | 0.03  (-0.04 to 0.09) | 0.05  (-0.02 to 0.11) | 1.63***  (1.59 to 1.68) | 1.64***  (1.59 to 1.69) | 1.64***  (1.59 to 1.69) |
| Bosnia and Herzegovina | 3.64***  (3.19 to 4.08) | 3.8***  (3.37 to 4.24) | 3.93***  (3.5 to 4.37) | 1.95***  (1.81 to 2.09) | 2.01***  (1.88 to 2.14) | 2.06***  (1.93 to 2.19) |
| Botswana | 0.89**  (0.37 to 1.42) | 1.01***  (0.47 to 1.55) | 1.13***  (0.57 to 1.69) | 1.91***  (1.85 to 1.97) | 1.94***  (1.89 to 2) | 1.98***  (1.94 to 2.02) |
| Brazil | -0.73***  (-0.89 to -0.56) | -0.69***  (-0.86 to -0.52) | -0.65***  (-0.83 to -0.48) | 0.41***  (0.29 to 0.53) | 0.38***  (0.26 to 0.49) | 0.38***  (0.26 to 0.51) |
| Brunei Darussalam | -2.58***  (-2.81 to -2.35) | -2.54***  (-2.79 to -2.3) | -2.47***  (-2.71 to -2.22) | 0.1  (-0.05 to 0.25) | 0.05  (-0.1 to 0.19) | 0.01  (-0.14 to 0.16) |
| Bulgaria | -0.4*  (-0.78 to -0.02) | -0.4  (-0.81 to 0) | -0.39  (-0.82 to 0.05) | 0.56***  (0.31 to 0.8) | 0.61***  (0.37 to 0.86) | 0.7***  (0.45 to 0.94) |
| Burkina Faso | -0.38***  (-0.5 to -0.26) | -0.37***  (-0.5 to -0.24) | -0.36***  (-0.5 to -0.22) | 1.34***  (1.26 to 1.42) | 1.3***  (1.23 to 1.38) | 1.27***  (1.2 to 1.34) |
| Burundi | -1.35***  (-1.58 to -1.12) | -1.38***  (-1.62 to -1.13) | -1.39***  (-1.65 to -1.13) | 0.11***  (0.08 to 0.13) | 0.1***  (0.07 to 0.13) | 0.1***  (0.07 to 0.13) |
| Cabo Verde | 3.73***  (2.9 to 4.57) | 3.97***  (3.12 to 4.82) | 4.2***  (3.34 to 5.07) | 2.25***  (2.16 to 2.35) | 2.23***  (2.13 to 2.33) | 2.2***  (2.1 to 2.29) |
| Cambodia | 0.21*  (0.05 to 0.36) | 0.16  (0 to 0.32) | 0.11  (-0.05 to 0.26) | 2.99***  (2.94 to 3.03) | 3***  (2.95 to 3.05) | 3***  (2.95 to 3.05) |
| Cameroon | 1.04***  (0.74 to 1.33) | 1.13***  (0.83 to 1.43) | 1.22***  (0.93 to 1.52) | 2.02***  (1.9 to 2.13) | 2.06***  (1.95 to 2.17) | 2.1***  (1.99 to 2.21) |
| Canada | -2.63***  (-3.3 to -1.96) | -2.56***  (-3.27 to -1.85) | -2.5***  (-3.25 to -1.73) | 2.11***  (2.03 to 2.18) | 2.08***  (2.01 to 2.15) | 2.05***  (1.98 to 2.11) |
| Central African Republic | 0.04  (-0.13 to 0.21) | 0.08  (-0.09 to 0.26) | 0.13  (-0.04 to 0.31) | 1.02***  (0.94 to 1.11) | 1.05***  (0.96 to 1.13) | 1.09***  (1.01 to 1.16) |
| Chad | 1.57***  (1.25 to 1.9) | 1.67***  (1.35 to 1.99) | 1.76***  (1.44 to 2.09) | 1.8***  (1.65 to 1.95) | 1.85***  (1.7 to 2) | 1.9***  (1.75 to 2.05) |
| Chile | -1***  (-1.43 to -0.56) | -0.86***  (-1.29 to -0.42) | -0.68**  (-1.09 to -0.27) | 2.51***  (2.34 to 2.68) | 2.51***  (2.33 to 2.69) | 2.59***  (2.41 to 2.76) |
| China | -0.48**  (-0.81 to -0.15) | -0.44*  (-0.79 to -0.09) | -0.39*  (-0.76 to -0.03) | 0.22  (-0.01 to 0.46) | 0.29*  (0.04 to 0.53) | 0.35*  (0.1 to 0.6) |
| Colombia | -2.66***  (-3.04 to -2.29) | -2.69***  (-3.09 to -2.29) | -2.71***  (-3.13 to -2.28) | 0.27***  (0.16 to 0.38) | 0.22***  (0.11 to 0.32) | 0.16**  (0.07 to 0.24) |
| Comoros | 0.04  (-0.02 to 0.09) | 0.03  (-0.03 to 0.08) | 0.01  (-0.05 to 0.07) | 0.6***  (0.52 to 0.68) | 0.61***  (0.53 to 0.69) | 0.63***  (0.55 to 0.71) |
| Congo | -0.07  (-0.31 to 0.16) | -0.02  (-0.26 to 0.23) | 0.04  (-0.21 to 0.29) | 1.22***  (1.16 to 1.28) | 1.24***  (1.18 to 1.3) | 1.26***  (1.2 to 1.32) |
| Cook Islands | -0.83***  (-0.91 to -0.75) | -0.85***  (-0.94 to -0.77) | -0.87***  (-0.96 to -0.79) | -0.72***  (-0.8 to -0.64) | -0.71***  (-0.79 to -0.62) | -0.72***  (-0.81 to -0.63) |
| Costa Rica | -0.74  (-1.54 to 0.08) | -0.98*  (-1.79 to -0.16) | -1.23**  (-2.04 to -0.42) | 0.94***  (0.86 to 1.02) | 0.97***  (0.89 to 1.05) | 0.98***  (0.89 to 1.06) |
| Côte d'Ivoire | 2.22***  (1.85 to 2.59) | 2.34***  (1.98 to 2.71) | 2.47***  (2.11 to 2.83) | 2.02***  (1.93 to 2.1) | 2.04***  (1.95 to 2.13) | 2.06***  (1.98 to 2.15) |
| Croatia | 0.78**  (0.29 to 1.28) | 0.7*  (0.18 to 1.22) | 0.6*  (0.06 to 1.14) | 0.35***  (0.27 to 0.43) | 0.35***  (0.26 to 0.43) | 0.36***  (0.27 to 0.45) |
| Cuba | -2.99***  (-3.66 to -2.32) | -3.08***  (-3.79 to -2.37) | -3.18***  (-3.92 to -2.43) | 0.59***  (0.5 to 0.67) | 0.55***  (0.47 to 0.62) | 0.54***  (0.46 to 0.62) |
| Cyprus | -3.33***  (-3.55 to -3.12) | -3.36***  (-3.59 to -3.14) | -3.4***  (-3.64 to -3.17) | -1.83***  (-1.88 to -1.77) | -1.84***  (-1.89 to -1.78) | -1.84***  (-1.9 to -1.78) |
| Czechia | 3.27***  (2.29 to 4.27) | 3.2***  (2.15 to 4.25) | 3.06***  (1.96 to 4.18) | 1.52***  (1.39 to 1.66) | 1.55***  (1.41 to 1.69) | 1.6***  (1.47 to 1.74) |
| Democratic People's Republic of Korea | 1.04***  (0.77 to 1.31) | 1.14***  (0.88 to 1.4) | 1.24***  (1 to 1.49) | 1.22***  (1.12 to 1.31) | 1.19***  (1.1 to 1.29) | 1.17***  (1.07 to 1.26) |
| Democratic Republic of the Congo | 0.59***  (0.47 to 0.72) | 0.63***  (0.51 to 0.75) | 0.67***  (0.54 to 0.79) | 1.1***  (1.06 to 1.15) | 1.1***  (1.06 to 1.15) | 1.11***  (1.07 to 1.16) |
| Denmark | 0.05  (-0.44 to 0.55) | 0.12  (-0.4 to 0.65) | 0.21  (-0.34 to 0.76) | 2.24***  (2.17 to 2.32) | 2.24***  (2.16 to 2.33) | 2.24***  (2.15 to 2.33) |
| Djibouti | 0.66***  (0.57 to 0.75) | 0.68***  (0.59 to 0.77) | 0.7***  (0.61 to 0.8) | 1.45***  (1.34 to 1.57) | 1.48***  (1.37 to 1.6) | 1.51***  (1.39 to 1.63) |
| Dominica | -0.2*  (-0.38 to -0.03) | -0.17  (-0.35 to 0.01) | -0.14  (-0.32 to 0.05) | 0.69***  (0.6 to 0.78) | 0.67***  (0.58 to 0.77) | 0.66***  (0.56 to 0.77) |
| Dominican Republic | 1.09***  (0.86 to 1.31) | 1.11***  (0.88 to 1.35) | 1.13***  (0.88 to 1.38) | 1.39***  (1.3 to 1.47) | 1.39***  (1.3 to 1.48) | 1.4***  (1.3 to 1.5) |
| Ecuador | 0.43  (-0.03 to 0.91) | 0.58*  (0.12 to 1.05) | 0.76**  (0.3 to 1.21) | 1.8***  (1.63 to 1.97) | 1.85***  (1.67 to 2.02) | 1.9***  (1.73 to 2.08) |
| Egypt | 2.75***  (2.36 to 3.15) | 2.89***  (2.5 to 3.28) | 3.03***  (2.65 to 3.41) | 3.86***  (3.77 to 3.94) | 3.89***  (3.8 to 3.97) | 3.9***  (3.81 to 3.99) |
| El Salvador | 2.39***  (2.2 to 2.58) | 2.45***  (2.26 to 2.64) | 2.49***  (2.29 to 2.69) | 1.6***  (1.51 to 1.7) | 1.61***  (1.51 to 1.71) | 1.6***  (1.5 to 1.71) |
| Equatorial Guinea | 0.31***  (0.19 to 0.44) | 0.33***  (0.2 to 0.47) | 0.34***  (0.2 to 0.49) | 2.46***  (2.3 to 2.61) | 2.51***  (2.36 to 2.66) | 2.57***  (2.42 to 2.71) |
| Eritrea | 0.74***  (0.66 to 0.83) | 0.76***  (0.67 to 0.85) | 0.77***  (0.68 to 0.87) | 1.33***  (1.22 to 1.43) | 1.35***  (1.24 to 1.46) | 1.39***  (1.28 to 1.5) |
| Estonia | 2.6***  (1.69 to 3.52) | 2.41***  (1.47 to 3.36) | 2.18***  (1.21 to 3.15) | 1.27***  (1.19 to 1.35) | 1.31***  (1.24 to 1.38) | 1.36***  (1.31 to 1.41) |
| Eswatini | 1.52***  (0.91 to 2.14) | 1.68***  (1.05 to 2.31) | 1.85***  (1.21 to 2.49) | 2.41***  (2.25 to 2.56) | 2.45***  (2.29 to 2.61) | 2.49***  (2.33 to 2.65) |
| Ethiopia | -2.43***  (-2.7 to -2.16) | -2.52***  (-2.79 to -2.26) | -2.62***  (-2.87 to -2.36) | -0.24***  (-0.29 to -0.19) | -0.25***  (-0.3 to -0.2) | -0.25***  (-0.31 to -0.2) |
| Fiji | 1.21***  (0.96 to 1.47) | 1.28***  (1.02 to 1.54) | 1.36***  (1.1 to 1.62) | 0.27  (-0.05 to 0.58) | 0.39*  (0.1 to 0.69) | 0.49**  (0.2 to 0.79) |
| Finland | -2.44***  (-2.99 to -1.88) | -2.6***  (-3.15 to -2.03) | -2.78***  (-3.33 to -2.22) | 0.67***  (0.59 to 0.75) | 0.65***  (0.57 to 0.73) | 0.63***  (0.55 to 0.71) |
| France | -0.44  (-0.89 to 0.02) | -0.38  (-0.86 to 0.1) | -0.33  (-0.84 to 0.18) | 0.16***  (0.12 to 0.2) | 0.14***  (0.1 to 0.18) | 0.12***  (0.09 to 0.15) |
| Gabon | -0.01  (-0.38 to 0.36) | 0.07  (-0.31 to 0.45) | 0.15  (-0.25 to 0.54) | 1.46***  (1.33 to 1.6) | 1.51***  (1.38 to 1.64) | 1.56***  (1.43 to 1.68) |
| Gambia | 2.04***  (1.84 to 2.24) | 2.1***  (1.89 to 2.3) | 2.15***  (1.94 to 2.36) | 1.93***  (1.87 to 1.99) | 1.94***  (1.88 to 2) | 1.95***  (1.89 to 2.02) |
| Georgia | 3.1***  (2.28 to 3.93) | 3.3***  (2.45 to 4.15) | 3.49***  (2.61 to 4.37) | 1.73***  (1.63 to 1.83) | 1.74***  (1.63 to 1.84) | 1.74***  (1.62 to 1.85) |
| Germany | -2.03***  (-2.25 to -1.81) | -2.05***  (-2.28 to -1.82) | -2.07***  (-2.32 to -1.83) | 0.74***  (0.57 to 0.92) | 0.67***  (0.5 to 0.84) | 0.59***  (0.44 to 0.74) |
| Ghana | 1.27***  (1.1 to 1.43) | 1.34***  (1.19 to 1.49) | 1.4***  (1.26 to 1.55) | 1.68***  (1.46 to 1.9) | 1.75***  (1.53 to 1.97) | 1.83***  (1.62 to 2.04) |
| Greece | -0.3  (-0.8 to 0.21) | -0.41  (-0.92 to 0.11) | -0.52  (-1.06 to 0.01) | -0.5***  (-0.61 to -0.38) | -0.51***  (-0.63 to -0.39) | -0.5***  (-0.63 to -0.37) |
| Greenland | -3.32***  (-3.54 to -3.1) | -3.38***  (-3.61 to -3.16) | -3.48***  (-3.69 to -3.28) | 2.22***  (1.92 to 2.53) | 2.11***  (1.82 to 2.41) | 2.01***  (1.72 to 2.3) |
| Grenada | -0.9***  (-1.19 to -0.61) | -0.94***  (-1.25 to -0.63) | -1***  (-1.32 to -0.68) | 1.14***  (1.02 to 1.25) | 1.17***  (1.06 to 1.29) | 1.22***  (1.11 to 1.32) |
| Guam | -2.83***  (-3.07 to -2.58) | -2.86***  (-3.12 to -2.6) | -2.86***  (-3.14 to -2.58) | -0.47***  (-0.7 to -0.25) | -0.55***  (-0.77 to -0.33) | -0.64***  (-0.85 to -0.42) |
| Guatemala | 3.29***  (2.71 to 3.88) | 3.44***  (2.85 to 4.04) | 3.6***  (2.99 to 4.21) | 1.74***  (1.39 to 2.1) | 1.84***  (1.48 to 2.2) | 1.93***  (1.56 to 2.3) |
| Guinea | 1.54***  (1.23 to 1.85) | 1.65***  (1.35 to 1.96) | 1.77***  (1.47 to 2.06) | 1.26***  (1.07 to 1.45) | 1.31***  (1.12 to 1.5) | 1.35***  (1.15 to 1.55) |
| Guinea-Bissau | 1.43***  (1.18 to 1.68) | 1.52***  (1.27 to 1.76) | 1.6***  (1.36 to 1.84) | 2.02***  (1.95 to 2.1) | 2.04***  (1.97 to 2.12) | 2.06***  (1.98 to 2.14) |
| Guyana | -0.55*  (-1.08 to -0.03) | -0.48  (-1.04 to 0.07) | -0.41  (-1 to 0.18) | 0.05  (-0.06 to 0.16) | 0.07  (-0.05 to 0.18) | 0.11  (-0.01 to 0.22) |
| Haiti | -0.17**  (-0.28 to -0.07) | -0.15*  (-0.26 to -0.04) | -0.13*  (-0.25 to -0.01) | 0.37***  (0.29 to 0.45) | 0.36***  (0.28 to 0.44) | 0.37***  (0.28 to 0.46) |
| Honduras | 2.64***  (2.29 to 2.99) | 2.73***  (2.37 to 3.09) | 2.82***  (2.45 to 3.19) | 1.62***  (1.48 to 1.76) | 1.66***  (1.53 to 1.8) | 1.72***  (1.59 to 1.85) |
| Hungary | 0.36  (0 to 0.72) | 0.36  (-0.03 to 0.74) | 0.32  (-0.09 to 0.73) | 0.57***  (0.32 to 0.83) | 0.64***  (0.38 to 0.9) | 0.74***  (0.49 to 0.99) |
| Iceland | -1.54***  (-1.77 to -1.32) | -1.53***  (-1.77 to -1.29) | -1.42***  (-1.65 to -1.2) | 1.67***  (1.62 to 1.72) | 1.66***  (1.61 to 1.7) | 1.64***  (1.6 to 1.69) |
| India | 1.67***  (1.44 to 1.9) | 1.66***  (1.42 to 1.91) | 1.62***  (1.37 to 1.88) | 0.94***  (0.85 to 1.03) | 0.94***  (0.85 to 1.04) | 0.95***  (0.85 to 1.05) |
| Indonesia | 0.8***  (0.58 to 1.01) | 0.85***  (0.63 to 1.07) | 0.91***  (0.68 to 1.13) | 3.19***  (2.8 to 3.58) | 3.39***  (3.05 to 3.73) | 3.61***  (3.35 to 3.86) |
| Iran (Islamic Republic of) | 2.41***  (2.24 to 2.59) | 2.5***  (2.35 to 2.65) | 2.57***  (2.43 to 2.71) | 2.74***  (2.59 to 2.88) | 2.79***  (2.65 to 2.94) | 2.8***  (2.65 to 2.95) |
| Iraq | -0.06  (-0.2 to 0.08) | -0.12  (-0.25 to 0.01) | -0.21***  (-0.3 to -0.12) | 1.78***  (1.7 to 1.86) | 1.81***  (1.74 to 1.89) | 1.82***  (1.74 to 1.9) |
| Ireland | -2.6***  (-2.82 to -2.38) | -2.55***  (-2.77 to -2.33) | -2.51***  (-2.75 to -2.28) | 1.21***  (1.14 to 1.27) | 1.2***  (1.13 to 1.26) | 1.18***  (1.11 to 1.24) |
| Israel | -0.73  (-1.64 to 0.19) | -0.62  (-1.58 to 0.35) | -0.49  (-1.51 to 0.53) | 0.78***  (0.5 to 1.07) | 0.88***  (0.6 to 1.17) | 0.99***  (0.72 to 1.26) |
| Italy | -1.25***  (-1.38 to -1.11) | -1.23***  (-1.37 to -1.09) | -1.22***  (-1.38 to -1.07) | -0.32***  (-0.42 to -0.21) | -0.36***  (-0.46 to -0.27) | -0.4***  (-0.49 to -0.31) |
| Jamaica | -0.34*  (-0.61 to -0.07) | -0.33*  (-0.62 to -0.04) | -0.32  (-0.62 to -0.01) | 0.84***  (0.78 to 0.9) | 0.86***  (0.8 to 0.93) | 0.89***  (0.83 to 0.95) |
| Japan | -2.16***  (-2.95 to -1.36) | -2.34***  (-3.15 to -1.51) | -2.5***  (-3.34 to -1.64) | 0.23*  (0.02 to 0.45) | 0.18  (-0.04 to 0.4) | 0.12  (-0.1 to 0.34) |
| Jordan | -2.57***  (-3.24 to -1.91) | -2.53***  (-3.24 to -1.82) | -2.46***  (-3.21 to -1.7) | 0.15*  (0.01 to 0.28) | 0.17*  (0.03 to 0.31) | 0.19*  (0.04 to 0.34) |
| Kazakhstan | -0.82**  (-1.3 to -0.34) | -0.8**  (-1.31 to -0.28) | -0.78**  (-1.32 to -0.23) | 2.98***  (2.89 to 3.07) | 3.01***  (2.92 to 3.1) | 3.04***  (2.95 to 3.13) |
| Kenya | 1.06***  (0.86 to 1.26) | 1.11***  (0.9 to 1.32) | 1.15***  (0.94 to 1.37) | 0.32***  (0.21 to 0.44) | 0.31***  (0.18 to 0.43) | 0.3***  (0.17 to 0.43) |
| Kiribati | 0.86***  (0.69 to 1.03) | 0.9***  (0.72 to 1.07) | 0.94***  (0.76 to 1.12) | -0.03  (-0.11 to 0.06) | 0  (-0.09 to 0.08) | 0.01  (-0.08 to 0.1) |
| Kuwait | -1.27***  (-1.85 to -0.68) | -1.2***  (-1.82 to -0.58) | -1.1**  (-1.75 to -0.44) | 2.21***  (2.06 to 2.35) | 2.22***  (2.07 to 2.38) | 2.23***  (2.06 to 2.39) |
| Kyrgyzstan | -0.19  (-0.68 to 0.3) | -0.17  (-0.69 to 0.35) | -0.16  (-0.71 to 0.4) | 2.38***  (2.28 to 2.47) | 2.41***  (2.31 to 2.5) | 2.43***  (2.34 to 2.53) |
| Lao People's Democratic Republic | -0.16*  (-0.31 to -0.01) | -0.14  (-0.3 to 0.02) | -0.12  (-0.28 to 0.05) | 2.56***  (2.54 to 2.59) | 2.57***  (2.54 to 2.6) | 2.58***  (2.55 to 2.61) |
| Latvia | 3.23***  (2.71 to 3.75) | 3.18***  (2.63 to 3.73) | 3.1***  (2.52 to 3.68) | 1.85***  (1.75 to 1.94) | 1.89***  (1.8 to 1.98) | 1.92***  (1.83 to 2.01) |
| Lebanon | -0.13*  (-0.23 to -0.03) | -0.13*  (-0.23 to -0.02) | -0.12*  (-0.23 to -0.01) | 1.07***  (1.02 to 1.12) | 1.05***  (1.01 to 1.1) | 1.05***  (1 to 1.1) |
| Lesotho | 3.72***  (3.08 to 4.37) | 3.91***  (3.26 to 4.57) | 4.1***  (3.44 to 4.76) | 3.18***  (3.01 to 3.35) | 3.24***  (3.07 to 3.41) | 3.29***  (3.12 to 3.46) |
| Liberia | 1.3***  (0.99 to 1.6) | 1.41***  (1.11 to 1.7) | 1.53***  (1.25 to 1.81) | 1.61***  (1.56 to 1.66) | 1.61***  (1.56 to 1.66) | 1.6***  (1.55 to 1.65) |
| Libya | 2.77***  (2.39 to 3.16) | 2.92***  (2.54 to 3.29) | 3.05***  (2.69 to 3.42) | 2.58***  (2.49 to 2.66) | 2.56***  (2.48 to 2.64) | 2.55***  (2.46 to 2.64) |
| Lithuania | 2.49***  (1.57 to 3.42) | 2.23***  (1.3 to 3.17) | 1.92***  (1 to 2.85) | 1.11***  (1 to 1.21) | 1.11***  (0.99 to 1.22) | 1.11***  (0.99 to 1.23) |
| Luxembourg | -1.67***  (-1.92 to -1.42) | -1.59***  (-1.83 to -1.34) | -1.52***  (-1.77 to -1.27) | 0.86***  (0.75 to 0.98) | 0.82***  (0.71 to 0.93) | 0.78***  (0.67 to 0.89) |
| Madagascar | -0.35***  (-0.42 to -0.28) | -0.36***  (-0.43 to -0.28) | -0.36***  (-0.44 to -0.28) | 0.77***  (0.72 to 0.82) | 0.78***  (0.72 to 0.83) | 0.8***  (0.76 to 0.85) |
| Malawi | -0.3*  (-0.58 to -0.02) | -0.28  (-0.58 to 0.02) | -0.27  (-0.58 to 0.05) | 0.24***  (0.19 to 0.29) | 0.24***  (0.18 to 0.29) | 0.24***  (0.18 to 0.3) |
| Malaysia | -1.91***  (-2.2 to -1.62) | -2.01***  (-2.29 to -1.73) | -2.04***  (-2.34 to -1.74) | 1.87***  (1.56 to 2.19) | 1.77***  (1.46 to 2.09) | 1.67***  (1.36 to 1.98) |
| Maldives | -2.55***  (-2.74 to -2.35) | -2.58***  (-2.78 to -2.38) | -2.59***  (-2.8 to -2.37) | 1.45***  (1.35 to 1.55) | 1.44***  (1.34 to 1.55) | 1.44***  (1.32 to 1.55) |
| Mali | 1.26***  (1.14 to 1.38) | 1.31***  (1.2 to 1.42) | 1.35***  (1.25 to 1.46) | 2***  (1.96 to 2.04) | 2.01***  (1.97 to 2.05) | 2.02***  (1.97 to 2.06) |
| Malta | -1.72***  (-2.14 to -1.3) | -1.88***  (-2.28 to -1.48) | -1.96***  (-2.38 to -1.54) | 0.76***  (0.45 to 1.07) | 0.7***  (0.39 to 1.02) | 0.64***  (0.31 to 0.97) |
| Marshall Islands | 1.12***  (0.78 to 1.46) | 1.17***  (0.81 to 1.54) | 1.24***  (0.86 to 1.61) | -0.11**  (-0.19 to -0.04) | -0.12**  (-0.2 to -0.04) | -0.15***  (-0.22 to -0.08) |
| Mauritania | 0.39***  (0.2 to 0.58) | 0.4***  (0.2 to 0.6) | 0.42***  (0.2 to 0.63) | 1.02***  (0.87 to 1.17) | 1.04***  (0.88 to 1.2) | 1.06***  (0.89 to 1.23) |
| Mauritius | 3.48***  (2.52 to 4.46) | 3.68***  (2.67 to 4.69) | 3.94***  (2.91 to 4.98) | 2.72***  (2.57 to 2.86) | 2.78***  (2.65 to 2.92) | 2.84***  (2.72 to 2.97) |
| Mexico | -0.75***  (-1 to -0.49) | -0.8***  (-1.07 to -0.54) | -0.86***  (-1.13 to -0.59) | -0.82***  (-1.01 to -0.63) | -0.87***  (-1.06 to -0.67) | -0.91***  (-1.11 to -0.71) |
| Micronesia (Federated States of) | 0.58**  (0.21 to 0.94) | 0.63**  (0.24 to 1.02) | 0.69**  (0.28 to 1.1) | 0.79***  (0.73 to 0.85) | 0.8***  (0.74 to 0.86) | 0.76***  (0.72 to 0.81) |
| Monaco | -0.34***  (-0.43 to -0.25) | -0.35***  (-0.44 to -0.25) | -0.35***  (-0.45 to -0.26) | 1.84***  (1.79 to 1.89) | 1.82***  (1.77 to 1.87) | 1.81***  (1.75 to 1.86) |
| Mongolia | 0.99***  (0.8 to 1.19) | 1.08***  (0.9 to 1.26) | 1.17***  (1.01 to 1.33) | 2.25***  (2.1 to 2.4) | 2.26***  (2.1 to 2.42) | 2.25***  (2.08 to 2.42) |
| Montenegro | 1.66***  (1.41 to 1.92) | 1.72***  (1.46 to 1.99) | 1.77***  (1.5 to 2.05) | 0.87***  (0.8 to 0.95) | 0.86***  (0.78 to 0.95) | 0.88***  (0.79 to 0.96) |
| Morocco | 2.69***  (2.48 to 2.91) | 2.77***  (2.55 to 2.98) | 2.83***  (2.62 to 3.05) | 2.09***  (2.03 to 2.16) | 2.13***  (2.08 to 2.18) | 2.13***  (2.08 to 2.19) |
| Mozambique | 0.85***  (0.69 to 1) | 0.89***  (0.73 to 1.05) | 0.92***  (0.76 to 1.09) | 1.06***  (0.99 to 1.14) | 1.08***  (0.99 to 1.16) | 1.09***  (1.01 to 1.18) |
| Myanmar | -0.45***  (-0.63 to -0.28) | -0.46***  (-0.64 to -0.28) | -0.47***  (-0.66 to -0.27) | 2.23***  (2.16 to 2.3) | 2.22***  (2.15 to 2.3) | 2.2***  (2.13 to 2.27) |
| Namibia | -0.01  (-0.35 to 0.34) | 0.03  (-0.34 to 0.4) | 0.07  (-0.32 to 0.46) | 0.87***  (0.8 to 0.94) | 0.89***  (0.82 to 0.95) | 0.89***  (0.82 to 0.97) |
| Nauru | 0.82***  (0.61 to 1.02) | 0.85***  (0.63 to 1.06) | 0.88***  (0.65 to 1.11) | 0.36***  (0.26 to 0.46) | 0.39***  (0.29 to 0.49) | 0.38***  (0.27 to 0.49) |
| Nepal | 1.4***  (1.2 to 1.6) | 1.37***  (1.16 to 1.58) | 1.32***  (1.1 to 1.54) | 2.11***  (2 to 2.21) | 2.16***  (2.07 to 2.26) | 2.22***  (2.15 to 2.29) |
| Netherlands | -2.95***  (-3.29 to -2.6) | -2.96***  (-3.33 to -2.59) | -2.97***  (-3.36 to -2.57) | -0.65***  (-0.74 to -0.56) | -0.67***  (-0.77 to -0.57) | -0.68***  (-0.78 to -0.58) |
| New Zealand | -2.07***  (-2.59 to -1.54) | -2.07***  (-2.63 to -1.51) | -2.01***  (-2.61 to -1.41) | 2.11***  (2 to 2.22) | 2.11***  (2 to 2.22) | 2.1***  (1.98 to 2.23) |
| Nicaragua | 0.63***  (0.35 to 0.91) | 0.73***  (0.46 to 1) | 0.83***  (0.57 to 1.1) | 1.16***  (1.11 to 1.2) | 1.16***  (1.12 to 1.21) | 1.17***  (1.12 to 1.22) |
| Niger | 1.08***  (1 to 1.17) | 1.1***  (1.01 to 1.19) | 1.11***  (1.02 to 1.2) | 1.92***  (1.84 to 1.99) | 1.93***  (1.85 to 2.01) | 1.95***  (1.87 to 2.04) |
| Nigeria | -0.6***  (-0.73 to -0.47) | -0.57***  (-0.71 to -0.44) | -0.54***  (-0.68 to -0.41) | 1.16***  (1.09 to 1.23) | 1.16***  (1.08 to 1.23) | 1.15***  (1.08 to 1.23) |
| Niue | 1.13***  (1.01 to 1.25) | 1.18***  (1.08 to 1.29) | 1.23***  (1.14 to 1.33) | -0.21**  (-0.34 to -0.09) | -0.21**  (-0.34 to -0.08) | -0.22**  (-0.36 to -0.08) |
| North Macedonia | 1.56***  (1.17 to 1.96) | 1.69***  (1.3 to 2.08) | 1.82***  (1.43 to 2.21) | 1.5***  (1.35 to 1.65) | 1.54***  (1.38 to 1.69) | 1.59***  (1.43 to 1.74) |
| Northern Mariana Islands | -1.06***  (-1.34 to -0.77) | -1.05***  (-1.35 to -0.75) | -1.03***  (-1.35 to -0.71) | 0.91***  (0.82 to 1) | 0.88***  (0.8 to 0.97) | 0.85***  (0.77 to 0.93) |
| Norway | -0.99**  (-1.64 to -0.35) | -0.87*  (-1.55 to -0.2) | -0.71  (-1.4 to -0.01) | 0.09  (-0.14 to 0.32) | 0.03  (-0.2 to 0.26) | -0.06  (-0.28 to 0.17) |
| Oman | -0.45***  (-0.63 to -0.27) | -0.39***  (-0.56 to -0.21) | -0.36***  (-0.54 to -0.17) | 2.2***  (1.97 to 2.44) | 2.32***  (2.11 to 2.53) | 2.42***  (2.23 to 2.61) |
| Pakistan | 1.31***  (1.01 to 1.61) | 1.37***  (1.06 to 1.68) | 1.43***  (1.11 to 1.76) | 3.51***  (3.3 to 3.71) | 3.61***  (3.43 to 3.79) | 3.71***  (3.57 to 3.86) |
| Palau | 0.58***  (0.39 to 0.77) | 0.64***  (0.45 to 0.82) | 0.71***  (0.53 to 0.89) | 0.13*  (0.01 to 0.25) | 0.17**  (0.05 to 0.29) | 0.2**  (0.07 to 0.32) |
| Palestine | 0.28  (0.01 to 0.56) | 0.37*  (0.1 to 0.65) | 0.48**  (0.22 to 0.75) | 1.76***  (1.69 to 1.83) | 1.78***  (1.71 to 1.85) | 1.78***  (1.71 to 1.86) |
| Panama | 0.47**  (0.18 to 0.77) | 0.53**  (0.22 to 0.84) | 0.59**  (0.27 to 0.91) | 0.93***  (0.85 to 1) | 0.94***  (0.86 to 1.01) | 0.93***  (0.85 to 1.02) |
| Papua New Guinea | 0.35***  (0.23 to 0.47) | 0.37***  (0.25 to 0.5) | 0.39***  (0.25 to 0.52) | 1.49***  (1.41 to 1.57) | 1.49***  (1.41 to 1.58) | 1.48***  (1.39 to 1.56) |
| Paraguay | 2.49***  (2.06 to 2.93) | 2.65***  (2.22 to 3.07) | 2.82***  (2.42 to 3.23) | 1.87***  (1.73 to 2) | 1.9***  (1.76 to 2.04) | 1.99***  (1.9 to 2.09) |
| Peru | 0.59***  (0.3 to 0.89) | 0.58***  (0.27 to 0.89) | 0.53**  (0.2 to 0.85) | 1.88***  (1.72 to 2.05) | 1.92***  (1.75 to 2.09) | 1.97***  (1.8 to 2.14) |
| Philippines | 1.28***  (1.2 to 1.35) | 1.29***  (1.21 to 1.37) | 1.3***  (1.21 to 1.38) | 0.57***  (0.51 to 0.63) | 0.57***  (0.51 to 0.64) | 0.57***  (0.5 to 0.64) |
| Poland | 0.23  (-0.17 to 0.62) | 0.14  (-0.27 to 0.55) | 0.04  (-0.38 to 0.46) | 1.1***  (0.93 to 1.28) | 1.11***  (0.92 to 1.29) | 1.15***  (0.95 to 1.34) |
| Portugal | -1.05***  (-1.34 to -0.75) | -1.01***  (-1.32 to -0.7) | -0.98***  (-1.31 to -0.65) | 1.21***  (1.13 to 1.28) | 1.21***  (1.13 to 1.29) | 1.22***  (1.13 to 1.31) |
| Puerto Rico | -1.23***  (-1.44 to -1.01) | -1.23***  (-1.46 to -1) | -1.18***  (-1.43 to -0.94) | -0.33***  (-0.41 to -0.25) | -0.34***  (-0.43 to -0.26) | -0.31***  (-0.4 to -0.23) |
| Qatar | -0.26  (-1.21 to 0.69) | -0.06  (-1.04 to 0.93) | 0.16  (-0.86 to 1.19) | 1.23***  (0.77 to 1.69) | 1.36***  (0.9 to 1.83) | 1.51***  (1.05 to 1.97) |
| Republic of Korea | -1.82***  (-2.52 to -1.13) | -1.75***  (-2.48 to -1.01) | -1.6***  (-2.37 to -0.83) | 2.22***  (2.13 to 2.31) | 2.22***  (2.13 to 2.32) | 2.24***  (2.14 to 2.34) |
| Republic of Moldova | -0.36  (-1.15 to 0.43) | -0.54  (-1.35 to 0.28) | -0.74  (-1.57 to 0.1) | 1.26***  (1.07 to 1.44) | 1.25***  (1.05 to 1.45) | 1.24***  (1.03 to 1.45) |
| Romania | 0.4*  (0.05 to 0.75) | 0.31  (-0.05 to 0.67) | 0.24  (-0.14 to 0.61) | 0.72***  (0.62 to 0.82) | 0.77***  (0.68 to 0.86) | 0.83***  (0.76 to 0.89) |
| Russian Federation | 3.86***  (2.32 to 5.42) | 3.53***  (1.95 to 5.15) | 3.15***  (1.53 to 4.81) | 1.85***  (1.78 to 1.93) | 1.89***  (1.83 to 1.95) | 1.91***  (1.84 to 1.97) |
| Rwanda | -2.18***  (-2.55 to -1.81) | -2.28***  (-2.65 to -1.91) | -2.38***  (-2.76 to -2.01) | 0.15***  (0.1 to 0.2) | 0.13***  (0.08 to 0.18) | 0.13***  (0.07 to 0.18) |
| Saint Kitts and Nevis | -2.41***  (-2.95 to -1.86) | -2.35***  (-2.93 to -1.77) | -2.27***  (-2.88 to -1.65) | 1.05***  (0.8 to 1.3) | 1.04***  (0.78 to 1.31) | 1.02***  (0.74 to 1.3) |
| Saint Lucia | -3.01***  (-3.42 to -2.61) | -3.19***  (-3.57 to -2.81) | -3.32***  (-3.69 to -2.95) | -0.64***  (-0.72 to -0.55) | -0.67***  (-0.75 to -0.6) | -0.69***  (-0.77 to -0.62) |
| Saint Vincent and the Grenadines | -2.15***  (-2.55 to -1.76) | -2.15***  (-2.57 to -1.73) | -2.16***  (-2.61 to -1.7) | -0.35***  (-0.42 to -0.27) | -0.38***  (-0.44 to -0.31) | -0.41***  (-0.47 to -0.35) |
| Samoa | 1.53***  (1.38 to 1.69) | 1.59***  (1.44 to 1.74) | 1.64***  (1.49 to 1.79) | 0.19**  (0.07 to 0.31) | 0.16*  (0.04 to 0.29) | 0.11  (-0.01 to 0.23) |
| San Marino | -1.41***  (-1.75 to -1.08) | -1.3***  (-1.63 to -0.97) | -1.19***  (-1.51 to -0.86) | 1.41***  (1.32 to 1.51) | 1.38***  (1.28 to 1.47) | 1.33***  (1.25 to 1.42) |
| Sao Tome and Principe | 0.55**  (0.21 to 0.89) | 0.65***  (0.31 to 1) | 0.75***  (0.4 to 1.1) | 1.75***  (1.71 to 1.79) | 1.74***  (1.7 to 1.78) | 1.72***  (1.68 to 1.75) |
| Saudi Arabia | -0.64***  (-0.77 to -0.51) | -0.61***  (-0.74 to -0.48) | -0.58***  (-0.71 to -0.44) | 1.87***  (1.83 to 1.9) | 1.85***  (1.82 to 1.89) | 1.84***  (1.81 to 1.87) |
| Senegal | 1.75***  (1.51 to 1.99) | 1.82***  (1.58 to 2.06) | 1.9***  (1.66 to 2.14) | 2.28***  (2.17 to 2.4) | 2.3***  (2.18 to 2.42) | 2.33***  (2.21 to 2.46) |
| Serbia | 0.89***  (0.63 to 1.15) | 0.94***  (0.66 to 1.21) | 0.99***  (0.7 to 1.28) | 0.58***  (0.47 to 0.68) | 0.6***  (0.48 to 0.71) | 0.61***  (0.49 to 0.73) |
| Seychelles | 1.6***  (1.25 to 1.94) | 1.73***  (1.41 to 2.06) | 1.94***  (1.69 to 2.19) | 3.15***  (3.06 to 3.25) | 3.19***  (3.09 to 3.28) | 3.21***  (3.1 to 3.31) |
| Sierra Leone | 1.85***  (1.48 to 2.21) | 1.96***  (1.59 to 2.32) | 2.08***  (1.71 to 2.44) | 1.95***  (1.88 to 2.01) | 1.96***  (1.9 to 2.03) | 1.98***  (1.91 to 2.04) |
| Singapore | -7.42***  (-8.53 to -6.29) | -7.34***  (-8.52 to -6.14) | -7.17***  (-8.41 to -5.9) | 0.89***  (0.76 to 1.03) | 0.88***  (0.74 to 1.02) | 0.88***  (0.73 to 1.04) |
| Slovakia | -1.09***  (-1.2 to -0.97) | -1.13***  (-1.23 to -1.02) | -1.16***  (-1.27 to -1.05) | 0.73***  (0.64 to 0.83) | 0.76***  (0.66 to 0.86) | 0.78***  (0.68 to 0.88) |
| Slovenia | -2.56***  (-3.27 to -1.85) | -2.62***  (-3.37 to -1.86) | -2.72***  (-3.51 to -1.92) | -0.7***  (-0.84 to -0.55) | -0.71***  (-0.86 to -0.55) | -0.69***  (-0.85 to -0.52) |
| Solomon Islands | 1.18***  (1.04 to 1.32) | 1.18***  (1.03 to 1.33) | 1.16***  (1 to 1.32) | 2.2***  (2 to 2.4) | 2.29***  (2.1 to 2.48) | 2.36***  (2.18 to 2.54) |
| Somalia | 0.41***  (0.37 to 0.45) | 0.41***  (0.37 to 0.45) | 0.41***  (0.36 to 0.45) | 1.14***  (1.05 to 1.23) | 1.16***  (1.07 to 1.25) | 1.19***  (1.09 to 1.28) |
| South Africa | 2.22***  (1.67 to 2.78) | 2.4***  (1.85 to 2.95) | 2.58***  (2.03 to 3.12) | 2.34***  (2.23 to 2.45) | 2.39***  (2.29 to 2.5) | 2.44***  (2.35 to 2.54) |
| South Sudan | -0.06  (-0.18 to 0.06) | -0.1  (-0.22 to 0.02) | -0.14*  (-0.26 to -0.02) | 0.44***  (0.33 to 0.54) | 0.43***  (0.32 to 0.54) | 0.43***  (0.31 to 0.55) |
| Spain | -2.16***  (-2.32 to -2) | -2.14***  (-2.31 to -1.97) | -2.14***  (-2.32 to -1.96) | 0.6***  (0.47 to 0.73) | 0.65***  (0.51 to 0.78) | 0.69***  (0.56 to 0.82) |
| Sri Lanka | 2***  (1.76 to 2.24) | 2.08***  (1.84 to 2.31) | 2.21***  (2.01 to 2.4) | 1.9***  (1.8 to 1.99) | 1.94***  (1.86 to 2.03) | 1.98***  (1.91 to 2.06) |
| Sudan | 1.34***  (1.23 to 1.46) | 1.37***  (1.25 to 1.49) | 1.38***  (1.25 to 1.51) | 2.06***  (2 to 2.11) | 2.06***  (2 to 2.11) | 2.04***  (1.99 to 2.1) |
| Suriname | 0.32**  (0.12 to 0.52) | 0.37**  (0.17 to 0.58) | 0.38**  (0.16 to 0.6) | 0.87***  (0.82 to 0.92) | 0.86***  (0.81 to 0.92) | 0.86***  (0.8 to 0.92) |
| Sweden | -0.23  (-0.52 to 0.05) | -0.19  (-0.49 to 0.11) | -0.14  (-0.46 to 0.17) | 0.94***  (0.89 to 1) | 0.96***  (0.9 to 1.01) | 0.97***  (0.92 to 1.03) |
| Switzerland | -3.54***  (-3.81 to -3.27) | -3.52***  (-3.81 to -3.24) | -3.53***  (-3.84 to -3.23) | 0.8***  (0.7 to 0.9) | 0.79***  (0.69 to 0.89) | 0.74***  (0.65 to 0.83) |
| Syrian Arab Republic | -0.84***  (-1.11 to -0.57) | -0.87***  (-1.15 to -0.59) | -0.91***  (-1.21 to -0.61) | 1.92***  (1.84 to 2.01) | 1.95***  (1.86 to 2.04) | 1.98***  (1.89 to 2.06) |
| Taiwan (Province of China) | -2.81***  (-3.41 to -2.21) | -2.8***  (-3.44 to -2.16) | -2.74***  (-3.41 to -2.06) | 0.41***  (0.27 to 0.54) | 0.42***  (0.28 to 0.57) | 0.43***  (0.28 to 0.59) |
| Tajikistan | 0.34  (-0.19 to 0.88) | 0.46  (-0.08 to 1.01) | 0.6*  (0.03 to 1.17) | 3.31***  (3.16 to 3.46) | 3.36***  (3.21 to 3.5) | 3.39***  (3.25 to 3.54) |
| Thailand | -0.81***  (-1.12 to -0.51) | -0.85***  (-1.17 to -0.53) | -0.89***  (-1.23 to -0.55) | 1.9***  (1.86 to 1.94) | 1.88***  (1.85 to 1.92) | 1.87***  (1.84 to 1.9) |
| Timor-Leste | 1.02***  (0.79 to 1.24) | 0.97***  (0.74 to 1.21) | 0.92***  (0.67 to 1.16) | 3.63***  (3.55 to 3.7) | 3.67***  (3.61 to 3.73) | 3.7***  (3.64 to 3.75) |
| Togo | 1.16***  (1.07 to 1.26) | 1.2***  (1.1 to 1.29) | 1.23***  (1.14 to 1.32) | 1.83***  (1.76 to 1.9) | 1.85***  (1.77 to 1.92) | 1.87***  (1.79 to 1.94) |
| Tokelau | 0.55***  (0.38 to 0.72) | 0.59***  (0.41 to 0.76) | 0.63***  (0.45 to 0.81) | -0.33***  (-0.47 to -0.18) | -0.36***  (-0.51 to -0.21) | -0.4***  (-0.55 to -0.25) |
| Tonga | 1.16***  (1.07 to 1.26) | 1.2***  (1.11 to 1.29) | 1.24***  (1.15 to 1.32) | -0.43***  (-0.54 to -0.31) | -0.45***  (-0.57 to -0.33) | -0.48***  (-0.61 to -0.36) |
| Trinidad and Tobago | -1.6***  (-1.79 to -1.41) | -1.65***  (-1.85 to -1.45) | -1.7***  (-1.9 to -1.49) | 0.45***  (0.31 to 0.58) | 0.49***  (0.35 to 0.62) | 0.55***  (0.43 to 0.67) |
| Tunisia | 1.91***  (1.73 to 2.08) | 1.97***  (1.8 to 2.15) | 2.04***  (1.87 to 2.2) | 2.34***  (2.22 to 2.47) | 2.39***  (2.28 to 2.51) | 2.44***  (2.33 to 2.56) |
| Türkiye | -0.09  (-0.53 to 0.35) | -0.06  (-0.52 to 0.41) | -0.04  (-0.53 to 0.46) | 1.02***  (0.96 to 1.08) | 1.02***  (0.96 to 1.08) | 1.01***  (0.94 to 1.07) |
| Turkmenistan | 1.66***  (1.26 to 2.07) | 1.68***  (1.25 to 2.11) | 1.68***  (1.22 to 2.14) | 2.25***  (2.16 to 2.34) | 2.26***  (2.16 to 2.36) | 2.29***  (2.19 to 2.39) |
| Tuvalu | 0.64***  (0.52 to 0.77) | 0.67***  (0.54 to 0.8) | 0.69***  (0.55 to 0.83) | 0.83***  (0.68 to 0.97) | 0.9***  (0.77 to 1.03) | 0.95***  (0.83 to 1.07) |
| Uganda | 0.05  (-0.23 to 0.33) | 0.07  (-0.22 to 0.37) | 0.1  (-0.21 to 0.42) | 0.53***  (0.45 to 0.62) | 0.54***  (0.45 to 0.63) | 0.55***  (0.46 to 0.65) |
| Ukraine | -1.98***  (-2.53 to -1.44) | -2.14***  (-2.69 to -1.6) | -2.32***  (-2.86 to -1.77) | 1.31***  (1.16 to 1.45) | 1.3***  (1.14 to 1.46) | 1.29***  (1.12 to 1.46) |
| United Arab Emirates | -0.34  (-1.12 to 0.45) | -0.07  (-0.84 to 0.71) | 0.24  (-0.5 to 0.99) | 0.57***  (0.28 to 0.85) | 0.63***  (0.34 to 0.93) | 0.69***  (0.38 to 1) |
| United Kingdom | -2***  (-2.26 to -1.75) | -2.05***  (-2.32 to -1.78) | -2.11***  (-2.39 to -1.83) | 0.76***  (0.67 to 0.85) | 0.74***  (0.64 to 0.83) | 0.72***  (0.62 to 0.82) |
| United Republic of Tanzania | 0.48***  (0.39 to 0.58) | 0.52***  (0.43 to 0.61) | 0.56***  (0.47 to 0.64) | 1.27***  (1.15 to 1.38) | 1.3***  (1.18 to 1.41) | 1.33***  (1.22 to 1.45) |
| United States of America | -2.29***  (-2.85 to -1.73) | -2.28***  (-2.87 to -1.68) | -2.23***  (-2.86 to -1.59) | 4.02***  (3.71 to 4.34) | 4.11***  (3.79 to 4.43) | 4.2***  (3.88 to 4.52) |
| United States Virgin Islands | -1.9***  (-2.18 to -1.62) | -1.91***  (-2.21 to -1.61) | -1.89***  (-2.2 to -1.57) | 0.06  (-0.11 to 0.22) | 0.08  (-0.1 to 0.25) | 0.14  (-0.03 to 0.31) |
| Uruguay | -0.05  (-0.13 to 0.03) | -0.06  (-0.14 to 0.03) | -0.03  (-0.11 to 0.06) | 1.88***  (1.73 to 2.02) | 1.82***  (1.68 to 1.97) | 1.8***  (1.65 to 1.95) |
| Uzbekistan | 3.16***  (2.62 to 3.71) | 3.32***  (2.78 to 3.87) | 3.49***  (2.94 to 4.04) | 4.53***  (4.34 to 4.71) | 4.62***  (4.45 to 4.79) | 4.68***  (4.51 to 4.84) |
| Vanuatu | 0.71***  (0.62 to 0.8) | 0.71***  (0.62 to 0.8) | 0.7***  (0.6 to 0.8) | 1.49***  (1.43 to 1.55) | 1.51***  (1.46 to 1.57) | 1.52***  (1.47 to 1.58) |
| Venezuela (Bolivarian Republic of) | 0.12  (-0.26 to 0.51) | 0.02  (-0.37 to 0.42) | -0.09  (-0.49 to 0.31) | 0.94***  (0.86 to 1.02) | 0.93***  (0.84 to 1.01) | 0.93***  (0.83 to 1.02) |
| Viet Nam | 0.4***  (0.24 to 0.55) | 0.44***  (0.28 to 0.59) | 0.48***  (0.32 to 0.64) | 1.85***  (1.76 to 1.95) | 1.84***  (1.74 to 1.93) | 1.83***  (1.72 to 1.93) |
| Yemen | 0.82***  (0.73 to 0.91) | 0.81***  (0.71 to 0.9) | 0.79***  (0.69 to 0.89) | 3.15***  (2.74 to 3.56) | 3.38***  (3.04 to 3.71) | 3.64***  (3.46 to 3.81) |
| Zambia | -0.51***  (-0.67 to -0.35) | -0.51***  (-0.68 to -0.35) | -0.53***  (-0.7 to -0.35) | 0.63***  (0.54 to 0.72) | 0.66***  (0.58 to 0.75) | 0.71***  (0.63 to 0.79) |
| Zimbabwe | 2.76***  (2 to 3.53) | 2.93***  (2.14 to 3.73) | 3.11***  (2.29 to 3.93) | 2.18***  (2.04 to 2.33) | 2.21***  (2.06 to 2.36) | 2.23***  (2.08 to 2.39) |

**Supplementary Figure 1: (A): The elbow method for optimal-K in K-means clustering; (B): The silhouette method for optimal-K in K-means clustering**

**Supplementary Figure 2: (A) The elbow method for optimal-K in hierarchical clustering; (B): The silhouette method for optimal-K in hierarchical clustering; (C): Dendrogram of hierarchical clusters; (D): The principal component visualization of hierarchical clusters across 204 countries**

**Supplementary Figure 3: Age-specific incidence rates of T2DM in postmenopausal women globally and across SDI regions.**

**Supplementary Figure 4: Age-specific death rates of T2DM in postmenopausal women globally and across SDI regions.**

**Supplementary Figure 5. Diagnostic performance plots for Random Forest and XGBoost models predicting incident T2DM burden among postmenopausal women globally. (A) Observed versus predicted incidence rates. (B) Residuals plotted against predicted values. (C) Density distribution of model residuals. (D) Absolute error plotted against predicted values with loess smoother.**

**Supplementary Figure 6: Comparison of incidence and mortality rate estimates in 2021, 2022 and 2023.**

**Supplementary Figure 7: Comparison of EAPC estimates from 1990 to 2021, 2022 and 2023.**
